# Proceedings from the 9^th^ annual conference on the science of dissemination and implementation: Washington, DC, USA. 14-15 December 2016

**DOI:** 10.1186/s13012-017-0575-y

**Published:** 2017-04-20

**Authors:** David Chambers, Lisa Simpson, Gila Neta, Ulrica von Thiele Schwarz, Antoinette Percy-Laurry, Gregory A. Aarons, Gila Neta, Ross Brownson, Amanda Vogel, Shannon Wiltsey Stirman, Kenneth Sherr, Rachel Sturke, Wynne E. Norton, Allyson Varley, David Chambers, Cynthia Vinson, Lisa Klesges, Suzanne Heurtin-Roberts, M. Rashad Massoud, Leighann Kimble, Arne Beck, Claire Neely, Jennifer Boggs, Carmel Nichols, Wen Wan, Erin Staab, Neda Laiteerapong, Nathalie Moise, Ravi Shah, Susan Essock, Margaret Handley, Amy Jones, Jay Carruthers, Karina Davidson, Lauren Peccoralo, Lloyd Sederer, Todd Molfenter, Ashley Scudder, Sarah Taber-Thomas, Kristen Schaffner, Amy Herschell, Eva Woodward, Jeffery Pitcock, Mona Ritchie, JoAnn Kirchner, Julia E. Moore, Sobia Khan, Shusmita Rashid, Jamie Park, Melissa Courvoisier, Sharon Straus, Daniel Blonigen, Allison Rodriguez, Luisa Manfredi, Andrea Nevedal, Joel Rosenthal, David Smelson, Christine Timko, Nicole Stadnick, Jennifer Regan, Miya Barnett, Anna Lau, Lauren Brookman-Frazee, Erick Guerrero, Karissa Fenwick, Yinfei Kong, Gregory Aarons, Rebecca Lengnick-Hall, Karissa Fenwick, Benjamin Henwood, Nina Sayer, Craig Rosen, Robert Orazem, Brandy Smith, Craig Rosen, Lindsey Zimmerman, David Lounsbury, Craig Rosen, Rachel Kimerling, Jodie A. Trafton, Steven Lindley, Rahul Bhargava, Hal Roberts, Laura Gibson, Gabriel J. Escobar, Vincent Liu, Benjamin Turk, Arona Ragins, Patricia Kipnis, Ashley Ketterer Gruszkowski, Michael W. Kennedy, Emily Rentschler Drobek, Lior Turgeman, Aleksandra Sasha Milicevic, Terrence L. Hubert, Larissa Myaskovsky, Youxu C. Tjader, Robert J. Monte, Kathryn G. Sapnas, Edmond Ramly, Diane R Lauver, Christie M Bartels, Shereef Elnahal, Andrea Ippolito, Hillary Peabody, Carolyn Clancy, Randall Cebul, Thomas Love, Douglas Einstadter, Shari Bolen, Brook Watts, Vera Yakovchenko, Angela Park, William Lukesh, Donald R. Miller, David Thornton, Mari-Lynn Drainoni, Allen L. Gifford, Shawna Smith, Julia Kyle, Mark S Bauer, Daniel Eisenberg, Celeste Liebrecht, Michelle Barbaresso, Amy Kilbourne, Elyse Park, Giselle Perez, Jamie Ostroff, Sarah Greene, Michael Parchman, Brian Austin, Eric Larson, Stefanie Ferreri, Chris Shea, Megan Smith, Kea Turner, Jennifer Bacci, Kyle Bigham, Geoffrey Curran, Stefanie Ferreri, Caity Frail, Cory Hamata, Terry Jankowski, Wendy Lantaff, Melissa Somma McGivney, Margie Snyder, Megan McCullough, Chris Gillespie, Beth Ann Petrakis, Ellen Jones, Angela Park, Carol VanDeusen Lukas, Adam Rose, Sarah J. Shoemaker, Geoffrey Curran, Jeremy Thomas, Benjamin Teeter, Holly Swan, Benjamin Teeter, Jeremy Thomas, Geoffrey Curran, Appathurai Balamurugan, Meghan Lane-Fall, Rinad Beidas, Laura Di Taranti, Sruthi Buddai, Enrique Torres Hernandez, Jerome Watts, Lee Fleisher, Frances Barg, Isomi Miake-Lye, Tanya Olmos, Emmeline Chuang, Hector Rodriguez, Gerald Kominski, Becky Yano, Stephen Shortell, Mary Hook, Linda Fleisher, Alexander Fiks, Katie Halkyard, Rachel Gruver, Emily Sykes, Kimberly Vesco, Kate Beadle, Joanna Bulkley, Ashley Stoneburner, Michael Leo, Amanda Clark, Joan Smith, Christopher Smyser, Maggie Wolf, Shamik Trivedi, Brian Hackett, Rakesh Rao, F. Sessions Cole, Rose McGonigle, Ann Donze, Enola Proctor, Amit Mathur, Kenneth Sherr, Emmanuela Gakidou, Stephen Gloyd, Carolyn Audet, Jose Salato, Sten Vermund, Rivet Amico, Stephanie Smith, Beatha Nyirandagijimana, Hildegarde Mukasakindi, Christian Rusangwa, Molly Franke, Giuseppe Raviola, Matthew Cummings, Elijah Goldberg, Savio Mwaka, Olive Kabajaasi, Adithya Cattamanchi, Achilles Katamba, Shevin Jacob, Nathan Kenya-Mugisha, J. Lucian Davis, Julie Reed, Rohit Ramaswamy, Gareth Parry, Sylvia Sax, Heather Kaplan, Keng-yen Huang, Sabrina Cheng, Susan Yee, Kimberly Hoagwood, Mary McKay, Donna Shelley, Gbenga Ogedegbe, Laurie Miller Brotman, Roman Kislov, John Humphreys, Gill Harvey, Paul Wilson, Robert Lieberthal, Colleen Payton, Mona Sarfaty, George Valko, Rendelle Bolton, Carol VanDeusen Lukas, Christine Hartmann, Nora Mueller, Sally K. Holmes, Barbara Bokhour, Sarah Ono, Benjamin Crabtree, Leah Gordon, William Miller, Bijal Balasubramanian, Leif Solberg, Deborah Cohen, Kate McGraw, Andrew Blatt, Demietrice Pittman, Megan McCullough, Christine Hartmann, Helen Kales, Dan Berlowitz, Teresa Hudson, Chris Gillespie, Christian Helfrich, Erin Finley, Ashley Garcia, Kristen Rosen, Claudina Tami, Don McGeary, Mary Jo Pugh, Jennifer Sharpe Potter, Christian Helfrich, Krysttel Stryczek, David Au, Steven Zeliadt, George Sayre, Chris Gillespie, Jennifer Leeman, Allison Myers, Jennifer Grant, Mary Wangen, Tara Queen, Alexandra Morshed, Elizabeth Dodson, Rachel Tabak, Ross C. Brownson, R. Chris Sheldrick, Thomas Mackie, Justeen Hyde, Laurel Leslie, Itzhak Yanovitzky, Matthew Weber, Nicole Gesualdo, Teis Kristensen, Cameo Stanick, Heather Halko, Caitlin Dorsey, Byron Powell, Bryan Weiner, Cara Lewis, Byron Powell, Bryan Weiner, Cameo Stanick, Heather Halko, Caitlin Dorsey, Cara Lewis, Bryan Weiner, Caitlin Dorsey, Cameo Stanick, Heather Halko, Byron Powell, Cara Lewis, Shannon Wiltsey Stirman, Patricia Carreno, Kera Mallard, Tasoula Masina, Candice Monson, Taren Swindle, Geoffrey Curran, Zachary Patterson, Leanne Whiteside-Mansell, Rochelle Hanson, Benjamin Saunders, Sonja Schoenwald, Angela Moreland, Sarah Birken, Byron Powell, Justin Presseau, Isomi Miake-Lye, David Ganz, Brian Mittman, Deborah Delevan, Erin Finley, Jennifer N. Hill, Sara Locatelli, Barbara Bokhour, Gemmae Fix, Jeffrey Solomon, Nora Mueller, Sherri L. Lavela, Victoria Scott, Jonathan Scaccia, Kassy Alia, Brittany Skiles, Abraham Wandersman, Paul Wilson, Anne Sales, Megan Roberts, Amy Kennedy, David Chambers, Muin J. Khoury, Nina Sperber, Lori Orlando, Janet Carpenter, Larisa Cavallari, Joshua Denny, Amanda Elsey, Fern Fitzhenry, Yue Guan, Carol Horowitz, Julie Johnson, Ebony Madden, Toni Pollin, Victoria Pratt, Tejinder Rakhra-Burris, Marc Rosenman, Corrine Voils, Kristin Weitzel, Ryanne Wu, Laura Damschroder, Christine Lu, Rachel Ceccarelli, Kathleen M. Mazor, Ann Wu, Alanna Kulchak Rahm, Adam H. Buchanan, Marci Schwartz, Cara McCormick, Kandamurugu Manickam, Marc S. Williams, Michael F. Murray, Ngoc-Cam Escoffery, Erin Lebow-Skelley, Hallie Udelson, Elaine Böing, Maria E. Fernandez, Richard J. Wood, Patricia Dolan Mullen, Jenita Parekh, Valerie Caldas, Elizabeth A. Stuart, Shalynn Howard, Gilo Thomas, Jacky M. Jennings, Jennifer Torres, Christine Markham, Ross Shegog, Melissa Peskin, Stephanie Craig Rushing, Amanda Gaston, Gwenda Gorman, Cornelia Jessen, Jennifer Williamson, Dianne Ward, Amber Vaughn, Ellie Morris, Stephanie Mazzucca, Regan Burney, Shoba Ramanadhan, Sara Minsky, Vilma Martinez-Dominguez, Kasisomayajula Viswanath, Megan Barker, Myra Fahim, Arezoo Ebnahmady, Rosa Dragonetti, Peter Selby, Margaret Farrell, Jordan Tompkins, Wynne Norton, Kaelin Rapport, Margaret Hargreaves, Rebekka Lee, Shoba Ramanadhan, Gina Kruse, Charles Deutsch, Emily Lanier, Ashley Gray, Aaron Leppin, Lori Christiansen, Karen Schaepe, Jason Egginton, Megan Branda, Charlene Gaw, Sara Dick, Victor Montori, Nilay Shah, Ariella Korn, Peter Hovmand, Karen Fullerton, Nancy Zoellner, Erin Hennessy, Alison Tovar, Ross Hammond, Christina Economos, Christi Kay, Julie Gazmararian, Emily Vall, Patricia Cheung, Padra Franks, Shannon Barrett-Williams, Paul Weiss, Christi Kay, Julie Gazmararian, Erica Hamilton, Patricia Cheung, Christi Kay, Emily Vall, Julie Gazmararian, Luana Marques, Louise Dixon, Emily Ahles, Sarah Valentine, Candice Monson, Derri Shtasel, Shannon Wiltsey Stirman, Ruben Parra-Cardona, Mary Northridge, Rucha Kavathe, Jennifer Zanowiak, Laura Wyatt, Hardayal Singh, Nadia Islam, Madalena Monteban, Darcy Freedman, Kimberly Bess, Colleen Walsh, Kristen Matlack, Susan Flocke, Heather Baily, Samantha Harden, NithyaPriya Ramalingam, VCE Physical Activity Leadership Team, Kassy Alia, Jonathan Scaccia, Victoria Scott, Rohit Ramaswamy, Abraham Wandersman, Rachel Gold, Erika Cottrell, Celine Hollombe, Katie Dambrun, Arwen Bunce, Mary Middendorf, Marla Dearing, Stuart Cowburn, Ned Mossman, Gerry Melgar, Suellen Hopfer, Michael Hecht, Anne Ray, Michelle Miller-Day, Rhonda BeLue, Greg Zimet, Eve-Lynn Nelson, Sandy Kuhlman, Gary Doolittle, Hope Krebill, Ashley Spaulding, Theodore Levin, Michael Sanchez, Molly Landau, Patricia Escobar, Nadia Minian, Peter Selby, Aliya Noormohamed, Laurie Zawertailo, Dolly Baliunas, Norman Giesbrecht, Bernard Le Foll, Andriy Samokhvalov, Zachary Meisel, Daniel Polsky, Bruce Schackman, Julia Mitchell, Kaitlyn Sevarino, Sarah Gimbel, Moses Mwanza, Marie Paul Nisingizwe, Catherine Michel, Lisa Hirschhorn, Meghan Lane-Fall, Rinad Beidas, Laura Di Taranti, Mahrukh Choudhary, Della Thonduparambil, Lee Fleisher, Frances Barg, Paul Meissner, Hilary Pinnock, Melanie Barwick, Christopher Carpenter, Sandra Eldridge, Gonzalo Grandes-Odriozola, Chris Griffiths, Jo Rycroft-Malone, Elizabeth Murray, Anita Patel, Aziz Sheikh, Stephanie J. C. Taylor, Brian Mittman, Martin Guilliford, Gemma Pearce, Diane Korngiebel, Kathleen West, Wylie Burke, Peggy Hannon, Jeffrey Harris, Kristen Hammerback, Marlana Kohn, Gary K. C. Chan, Riki Mafune, Amanda Parrish, Christian Helfrich, Shirley Beresford, K. Joanne Pike, Rachel Shelton, Lina Jandorf, Deborah Erwin, Thana-Ashley Charles, Michael Parchman, Laura-Mae Baldwin, Brooke Ike, Jacqueline Fickel, Jason Lind, Diane Cowper, Marguerite Fleming, Amy Sadler, Melinda Dye, Judith Katzburg, Michael Ong, Sarah Tubbesing, Megan McCullough, Molly Simmons, Vera Yakovchenko, Autumn Harnish, Sonya Gabrielian, Keith McInnes, Jeffrey Smith, David Smelson, John Ferrand, Elisa Torres, Amy Green, Gregory Aarons, Angela R. Bradbury, Linda J. Patrick-Miller, Brian L. Egleston, Susan M. Domchek, Olufunmilayo I. Olopade, Michael J. Hall, Mary B. Daly, Linda Fleisher, Generosa Grana, Pamela Ganschow, Dominique Fetzer, Amanda Brandt, Rachelle Chambers, Dana F. Clark, Andrea Forman, Rikki S. Gaber, Cassandra Gulden, Janice Horte, Jessica Long, Terra Lucas, Shreshtha Madaan, Kristin Mattie, Danielle McKenna, Susan Montgomery, Sarah Nielsen, Jacquelyn Powers, Kim Rainey, Christina Rybak, Christina Seelaus, Jessica Stoll, Jill Stopfer, Xinxin Shirley Yao, Michelle Savage, Edward Miech, Teresa Damush, Nicholas Rattray, Jennifer Myers, Barbara Homoya, Kate Winseck, Carrie Klabunde, Deb Langer, Avi Aggarwal, Elizabeth Neilson, Lara Gunderson, Gabriel J. Escobar, Marla Gardner, Liam O’Sulleabhain, Candyce Kroenke, Vincent Liu, Patricia Kipnis

**Affiliations:** 1 Division of Cancer Control and Population Sciences, National Cancer Institute, Rockville, MD 20850 USA; 2 AcademyHealth, Washington, DC 20009 USA; 3 Department of Learning, Informatics, Management, and Ethics, Karolinska Institutet, Stockholm, 17177 Sweden; 4 Division of Cancer Control and Population Sciences, National Cancer Institute, Rockville, MD 20850 USA; 5 Department of Psychiatry, University of California San Diego, San Diego, CA 92093 USA; 6 Division of Cancer Control and Population Sciences, National Cancer Institute, Rockville, MD 20850 USA; 7 George Warren Brown School of Social Work, Washington University in St. Louis, St. Louis, MO 63130 USA; 8 Center for Global Health, National Cancer Institute, Rockville, MD 20850 USA; 9 Dissemination and Training Division, VA National Center for PTSD, Palo Alto, CA 94305 USA; 10 Department of Global Health, University of Washington, Seattle, WA 98105 USA; 11 Center for Global Health Studies, Fogarty International Center, National Institutes of Health, Bethesda, MD 20912 USA; 12 Division of Cancer Control and Population Sciences, National Cancer Institute, Rockville, MD 20850 USA; 13 School of Public Health, University of Alabama at Birmingham, Birmingham, AL 35243 USA; 14 Division of Cancer Control and Population Sciences, National Cancer Institute, Rockville, MD 20850 USA; 15 School of Public Health, University of Memphis, Memphis, TN 38152 USA; 16 USAID Applying Science to Strengthen and Improve Systems (ASSIST) Project, Bethesda, Maryland 20814 USA; 17 Institute for Health Research, Kaiser Permanente, Denver, CO 80231 USA; 18 ICSI, Institute for Clinical Systems Improvement, Bloomington, MN 55425 USA; 19 Medicine, University of Chicago, Chicago, IL 60637 USA; 20 Medicine, Columbia University Medical Center, New York, NY 10032 USA; 21 Psychiatry, Columbia University, New York, NY 10032 USA; 22 General Internal Medicine, Epidemiology and Biostatistics, University of California San Francisco, San Francisco, CA 94110 USA; 23 New York State, Office of Mental Health, New York, NY 10001 USA; 24 Medicine, Icahn School of Medicine at Mount Sinai, New York, NY 10029 USA; 25 Center for Health Enhancement System Studies, University of Wisconsin, Madison, WI 53706 USA; 26 Department of Psychiatry, University of Pittsburgh, Pittsburgh, PA 15213 USA; 27 Psychology, University at Buffalo, Buffalo, NY 14260 USA; 28 Department of Psychology, West Virginia University, Morgantown, WV 26506 USA; 29 MIRECC, VA, North Little Rock, AR 72114 USA; 30 QUERI for Team-Based Behavioral Health, Central Arkansas Veterans Healthcare System, North Little Rock, AR 72114 USA; 31 Department of Psychiatry and Behavioral Sciences, University of Arkansas for Medical Sciences, Little Rock, AR 72205 USA; 32 Knowledge Translation (KT) Program, Li Ka Shing Knowledge Institute, St. Michael’s Hospital, Toronto, ON M5B 1 T8 Canada; 33 Geriatric Medicine, University of Toronto, Toronto, ON M5G 1 V7 Canada; 34 HSR&D Center for Innovation to Implementation, Department of Veterans Affairs Palo Alto Health Care System, Menlo Park, CA 94025 USA; 35 Clinical Psychology PhD Program, Palo Alto University, Palo Alto, CA 94304 USA; 36 VA Palo Alto Health Care System, National Center for PTSD, Menlo Park, CA 94025 USA; 37 Health Services Research and Development, Veterans Health Administration, Menlo Park, CA 94025 USA; 38 Office of Homelessness/Veterans Justice Programs, Department of Veterans Affairs, Menlo Park, CA 94025 USA; 39 Center for Healthcare Organization and Implementation Research (CHOIR), VA Health Services Research & Development, Bedford/Boston, MA 01730 USA; 40 Department of Psychiatry, University of Massachusetts Medical School, Worcester, MA 01655 USA; 41 Psychiatry, University of California, San Diego, La Jolla, CA 92093 USA; 42 Clinical Training and Evidence-Based Practice, Hathaway-Sycamores Child and Family Services, Pasadena, CA 91105 USA; 43 Counseling, Clinical, & School Psychology, University of California, Santa Barbara, Santa Barbara, CA 93106-9490 USA; 44 Psychology, University of California Los Angeles, Los Angeles, CA 90095 USA; 45 Communities, Organizations and Business Innovation, School of Social Work, and Marshall School of Business, University of Southern California, Los Angeles, CA 90015 USA; 46 School of Social Work, University of Southern California, Los Angeles, CA 90015 USA; 47 Mihaylo College of Business and Economics, California State University, Fullerton, CA 90089 USA; 48 Psychiatry, UC San Diego, La Jolla, CA 92083-0812 USA; 49 School of Social Work, University of Southern California, Los Angeles, CA 90015 USA; 50 Center for Chronic Disease Outcomes Research, Minneapolis VA Health Care System, Minneapolis, MN 55417 USA; 51 Medicine and Psychiatry, University of Minnesota, Minneapolis, MN 55454 USA; 52 VA Palo Alto Health Care System, National Center for PTSD Dissemination & Training Division, Menlo Park, CA 94025 USA; 53 Department of Psychiatry and Behavioral Sciences, Stanford University School of Medicine, Stanford, CA 94304 USA; 54 VA Palo Alto Health Care System, Center for Innovation to Implementation, Menlo Park, CA 94403 USA; 55 VA Palo Alto Health Care System, National Center for PTSD, Dissemination and Training Division, Menlo Park, CA 94025 USA; 56 Psychiatry and Behavioral Sciences, Stanford University School of Medicine, Menlo Park, CA 94025 USA; 57 Veterans Affairs Palo Alto Healthcare System, Center for Innovation to Implementation, Menlo Park, CA 94025 USA; 58 VA Palo Alto Health Care System, National Center for PTSD, Dissemination and Training Division, Menlo Park, CA 94025 USA; 59 Psychiatry and Behavioral Sciences, University of Washington School of Medicine, Seattle, WA 98195 USA; 60 Epidemiology and Population Health, Albert Einstein College of Medicine, Bronx, NY 10467 USA; 61 Division of Community Collaboration and Implementation Science, Albert Einstein College of Medicine of Yeshiva University, Bronx, NY 10467 USA; 62 Psychiatry and Behavioral Sciences, Stanford University School of Medicine, Menlo Park, CA 94025 USA; 63 Veterans Affairs Palo Alto Healthcare System, Center for Innovation to Implementation, Menlo Park, CA 94025 USA; 64 VA Program Evaluation and Resource Center, VA Office of Mental Health Operations, Menlo Park, CA 94025 USA; 65 Outpatient Mental Health, Veterans Affairs Palo Alto Healthcare System, Menlo Park, CA 94025 USA; 66 Media Lab, Massachusetts Institute of Technology, Cambridge, MA 02139 USA; 67 Berkman Klein Center for Internet & Society, Harvard Law School, Cambridge, MA 02138 USA; 68 Berkman Klein Center for Internet and Society, Harvard University, Cambridge, MA 02138 USA; 69 Tobacco Center of Regulatory Science, University of Pennsylvania School for Communication, Philadelphia, PA 19104 USA; 70 Division of Research, Kaiser Permanente, Oakland, CA 94612 USA; 71 Health Care Effectiveness, Kaiser Permanente Division of Research, Oakland, CA 94612 USA; 72 Decision Support, Kaiser Permanente Northern California, Oakland, CA 94612 USA; 73 Veterans Engineering Resource Center, VA Pittsburgh Healthcare System, Pittsburgh, PA 15125 USA; 74 Joseph M. Katz Graduate School of Business, University of Pittsburgh, Pittsburgh, PA 15260 USA; 75 Center for Health Equity Research and Promotion, VA Pittsburgh Healthcare System, Pittsburgh, PA 15240 USA; 76 University of Pittsburgh School of Medicine, University of Pittsburgh, Pittsburgh, PA 15240 USA; 77 Office of the Assistant Deputy Under Secretary of Health for Patient Care Services, VA Central Office, Washington, DC 20420 USA; 78 Industrial and Systems Engineering, University of Wisconsin-Madison College of Engineering, Madison, WI 53704 USA; 79 Nursing, University of Wisconsin School of Nursing, Madison, WI 53704 USA; 80 Medicine, Rheumatology Division, University of Wisconsin School of Medicine and Public Health, Madison, WI 53705 USA; 81 Office of the Under Secretary for Health, Organizational Excellence, US Department of Veterans Affairs, Washington, DC 20005 USA; 82 Office of Under Secretary for Health, Veterans Health Administration, Washington, DC 20005 USA; 83 Healthcare Performance Improvement and Innovation, Atlas Research, Washington, DC 20005 USA; 84 Center for Health Care Research and Policy, Case Western Reserve University School of Medicine, Cleveland, OH 44109 USA; 85 Medicine and Epidemiology and Biostatistics, Better Health Partnership, Cleveland, OH 44109 USA; 86 MetroHealth Medical Center, Cleveland, OH 44109 USA; 87 Medicine, Better Health Partnership, Cleveland, OH 44109 USA; 88 Informatics and Analytics, Louis Stokes Cleveland VA Medical Center, Cleveland, OH 44106 USA; 89 Department of Medicine, Case Western Reserve University, Cleveland, OH 44106 USA; 90 Department of Health Law, Policy and Management, Boston University School of Public Health, Boston, MA 02118 USA; 91 Department of Veterans Affairs, VA HSR&D Center for Healthcare Organization and Implementation Research (CHOIR), Bedford, MA 01730 USA; 92 Boston VA Healthcare System, New England Veterans Engineering Resource Center, Boston, MA 02132 USA; 93 VA Boston Healthcare System, Boston, MA 02139 USA; 94 Department of Medicine, Boston University School of Medicine, Boston, MA 02118 USA; 95 Internal Medicine, Boston University School of Medicine, Boston, MA 02215 USA; 96 Internal Medicine, Division of General Medicine, University of Michigan, Ann Arbor, MI 48109 USA; 97 Psychiatry, University of Michigan Medical School, Ann Arbor, MI 48109 USA; 98 Center for Healthcare Organization and Implementation Research, VA HSR&D, Bedford/Boston, MA 02130 USA; 99 Department of Psychiatry, Harvard Medical School, Boston, MA 02115 USA; 100 Health Management and Policy, University of Michigan School of Public Health, Ann Arbor, MI 48109 USA; 101 Center for Clinical Management Research, VA Ann Arbor Health System, Ann Arbor, MI 48109 USA; 102 Dept of Veterans Affairs, VA Quality Enhancement Research Initiative (QUERI), Washington, DC 20420 USA; 103 VA Health Services Research and Development, VA Quality Enhancement Research Initiative, Washington, DC 20420 USA; 104 MIHP, MGH, Boston, MA 02114 USA; 105 Psychiatry, Memorial Sloan Kettering Cancer Center, New York, NY 10022 USA; 106 Research, Health Care Systems Research Network, Seattle, WA 98115 USA; 107 MacColl Center for Health Care Innovation and Senior Investigator, Group Health Research Institute, Seattle, WA 98101 USA; 108 Division of Practice Advancement and Clinical Education, University of North Carolina Eshelman School of Pharmacy, Chapel Hill, NC 27599 USA; 109 Health Policy and Management, The University of North Carolina at Chapel Hill, Chapel Hill, NC 27599 USA; 110 Pharmacy Practice, University of Arkansas for Medical Sciences College of Pharmacy, Little Rock, AR 72205 USA; 111 Health Policy and Management, University of North Carolina Gillings School of Global Public Health, Chapel Hill, NC 27599 USA; 112 Pharmacy Practice, University of Washington School of Pharmacy, Seattle, WA 98195 USA; 113 Department of Pharmacy Practice, University of Arkansas for Medical Sciences, Little Rock, AR 72205 USA; 114 Division of Practice Advancement and Clinical Education, University of North Carolina Eshelman School of Pharmacy, Chapel Hill, NC 27599 USA; 115 Pharmacy Practice, University of Minnesota College of Pharmacy, Minneapolis, MN 55455 USA; 116 Library Science, University of Washington, Seattle, WA 98195 USA; 117 Practice, Purdue University College of Pharmacy, Indianapolis, IN 46202 USA; 118 Pharmacy Practice, University of Pittsburg School of Pharmacy, Pittsburg, PA 15261 USA; 119 Center for Healthcare Organization and Implementation Research (CHOIR), VA Health Services Research & Development, Bedford, MA 01730 USA; 120 Department of Health Law, Policy and Management, Boston University School of Public Health, Boston, MA 02118 USA; 121 Pharmacy, Central Western Massachusetts VA Healthcare System, Leeds, MA 01053 USA; 122 Boston VA Healthcare System, New England Veterans Engineering Resource Center, Boston, MA 02130 USA; 123 Research, VA Boston Healthcare System, Center for Healthcare Organization and Implementation Research, Boston, MA 02130 USA; 124 Health Policy and Management, Boston University School of Public Health, Boston, MA 02118 USA; 125 Internal Medicine, Boston University Medical Center, Boston, MA 02119 USA; 126 US Health, Abt Associates, Cambridge, MA 02138 USA; 127 Department of Pharmacy Practice, University of Arkansas for Medical Sciences, Little Rock, AR 72205 USA; 128 Department of Pharmacy Practice, University of Arkansas for Medical Sciences, Little Rock, AR 72205 USA; 129 Center for Health Advancement, Arkansas Department of Health, Little Rock, AR 72205 USA; 130 Anesthesiology and Critical Care, Perelman School of Medicine, University of Pennsylvania, Philadelphia, PA 19104-4865 USA; 131 Leonard Davis Institute of Health Economics, University of Pennsylvania, Philadelphia, PA 19104 USA; 132 Center for Healthcare Improvement and Patient Safety, Perelman School of Medicine, University of Pennsylvania, Philadelphia, PA 19104 USA; 133 Psychiatry, University of Pennsylvania, Philadelphia, PA 19104 USA; 134 School of Science, Engineering and Technology, St. Mary’s University, San Antonio, TX 78228 USA; 135 Haverford College, Haverford, PA 19041 USA; 136 Family Medicine and Community Health, University of Pennsylvania, Philadelphia, PA 19104 USA; 137 Health Policy and Management, UCLA Fielding School of Public Health, Los Angeles, CA 90024 USA; 138 Health Policy and Management, University of California, Los Angeles, San Fernando, CA 91340 USA; 139 Health Policy and Management, University of California - Berkeley, School of Public Health, Berkeley, CA 94720 USA; 140 Department of Health Policy and Management, UCLA Center for Health Policy Research, Los Angeles, CA 90272 USA; 141 HSR&D Center for the Study of Healthcare Innovation, Implementation & Policy (CSHIIP), VA Greater Los Angeles Healthcare System, Sepulveda, CA 91343 USA; 142 School of Public Health, University of California, Berkeley, Berkeley, CA 94720 USA; 143 Center for Nursing Research and Practice, Aurora Health Care, Milwaukee, WI 53233 USA; 144 Research Institute, Children’s Hospital of Philadelphia, Philadelphia, PA 19104 USA; 145 Population Science, Fox Chase Cancer Center, Philadelpia, PA 19015 USA; 146 General Pediatrics, University of Pennsylvania, Philadelphia, PA 19066 USA; 147 Science Programs Department, Kaiser Permanente Center for Health Research, Northwest, Portland, OR 97227 USA; 148 Obstetrics & Gynecology, Northwest Permanente, Clackamas, OR 97015 USA; 149 Obstetrics & Gynecology, Kaiser Permanente, Northwest, Portland, OR 97227 USA; 150 Nursing, St. Louis Children’s Hospital, St. Louis, MO 63110 USA; 151 Neurology, Washington University School of Medicine, St. Louis, MO 63110 USA; 152 Neonatal-Perinatal Medicine, Washington University School of Medicine, St. Louis, MO 63110 USA; 153 Pediatrics and Division of Newborn Medicine, Washington University School of Medicine, St. Louis, MO 63110 USA; 154 Newborn Intensive Care Unit, St. Louis Children’s Hospital, St. Louis, MO 63110 USA; 155 Nursing/Newborn Intensive Care Unit, St. Louis Children’s Hospital, St. Louis, MO 63110 USA; 156 George Warren Brown School of Social Work, Washington University in St. Louis, St. Louis, MO 63130 USA; 157 Pediatrics and Newborn Medicine, Washington University School of Medicine, St. Louis, MO 63110 USA; 158 Global Health, University of Washington, Seattle, WA 98105 USA; 159 Health Policy, Vanderbilt University Medical Center, Nashville, TN 37209 USA; 160 Community Health, Friends in Global Health, Quelimane, 00000 Mozambique; 161 Department of Pediatrics, Vanderbilt University Medical Center, Nashville, TN 37203 USA; 162 Health Behavior Health Education, University of Michigan, Ann Arbor, MI 48109 USA; 163 Mental Health, Partners In Health, Boston, MA 02199 USA; 164 Psychiatry, Brigham and Women’s Hospital, Boston, MA 02115 USA; 165 Mental Health, Inshuti Mu Buzima, Butaro, Rwanda; 166 Health Systems Strengthening, Inshuti Mu Buzima, Butaro, Rwanda; 167 Department of Global Health and Social Medicine, Harvard Medical School, Boston, MA 02115 USA; 168 Medicine, New York Presbyterian, Columbia University Medical Center, New York, NY 10032 USA; 169 Implementation, Walimu, Kampala, Uganda; 170 Medicine, University of California San Francisco, San Francisco, CA 94110 USA; 171 Medicine, Makerere College of Health Sciences, Kampala, Uganda; 172 Medicine, University of Washington, Seattle, WA 98104 USA; 173 Epidemiology (Microbial Diseases), Yale School of Public Health, New Haven, CT 06520-8034 USA; 174 Public Health and Primary Care, NIHR CLAHRC NWL, London, SW10 9NH UK; 175 Gillings School of Public Health, University of North Carolina, Chapel Hill, NC 27599 USA; 176 Institute for Healthcare Improvement, Boston, MA 02138 USA; 177 Independent Global Consultant, Heidelberg, 69118 Germany; 178 Divisions of Neonatology, Cincinnati Children’s Hospital Medical Center, Cincinnati, OH 45229 USA; 179 Populaiton Health, Population Health at NYU Langone Medical Center, New York, NY 10016 USA; 180 Child & Adolescent Psychiatry, The Child Study Center at NYU Langone Medical Center, New York, NY 10016 USA; 181 Social Work, McSilver Institute for Poverty Policy and Research, New York, NY 10013 USA; 182 Social Work, New York University, New York, NY 10013 USA; 183 Population Health and Medicine and Center for Healthful Behavior Change, New York University, New York, NY 10016 USA; 184 Alliance Manchester Business School, University of Manchester, Manchester, M15 6 PB UK; 185 NIHR CLAHRC Greater Manchester, Salford Royal NHS Foundation Trust, Salford, M6 8HD UK; 186 School of Nursing, The University of Adelaide, Adelaide, SA 5005 Australia; 187 Jefferson School of Population Health, Thomas Jefferson University, Philadelphia, PA 19107 USA; 188 Sidney Kimmel Cancer Center, Thomas Jefferson University, Philadelphia, PA 19107 USA; 189 Department of Public Health, University of Tennessee Knoxville, Knoxville, TN 37996 USA; 190 Department of Family and Community Medicine, Thomas Jefferson University, Philadelphia, PA 19107 USA; 191 ENRM Veterans Hospital, VA HSR&D Center for Healthcare Organization and Implementation Research (CHOIR), Bedford, MA 01730 USA; 192 Center for Organization, Leadership & Mgmt. Rsch., Department of Veterans Affairs/Boston University-School of Public Health, Boston, MA 02130 USA; 193 Department of Veterans Affairs, Center for Healthcare Organization and Implementation Research (CHOIR), Bedford, MA 01730 USA; 194 Health Law, Policy and Management, Boston University School of Public Health, Boston, MA 02118 USA; 195 Center for Healthcare Organization and Implementation Research, VA HSR&D Center for Healthcare Organization and Implementation Research (CHOIR), Bedford, MA 01730 USA; 196 Research, VA HSR&D Center for Healthcare Organization and Implementation Research, Bedford/Boston, MA 02130 USA; 197 Department of Family Medicine, Oregon Health & Science University, Portland, OR 97239 USA; 198 VA Portland Health Care System, Department of Veterans Affairs, Portland, OR 97239 USA; 199 Department of Family Medicine and Community Health, Rutgers Robert Wood Johnson Medical School, New Brunswick, NJ 08901 USA; 200 Family Medicine, Lehigh Valley Hospital, Allentown, PA 18105-7017 USA; 201 Epidemiology, University of Texas Health Science Center at Houston, Houston, TX 77030 USA; 202 Institute for Education and Research, HealthPartners, Bloomington, MN 55425 USA; 203 DHCC, Defense Centers of Excellence for Psychological Health and Traumatic Brain Injury, Silver Spring, MD 20910 USA; 204 DHCC/PHCC, Defense Centers of Excellence for Psychological Health & TBI, Silver Spring, MD 20901 USA; 205 Implementation Science Team, Defense Centers of Excellence for Psychological Health and Traumatic Brain Injury (DCoE), Silver Spring, MD 20910 USA; 206 Center for Healthcare Organization and Implementation Research (CHOIR), VA Health Services Research & Development, Bedford, MA 01730 USA; 207 Department of Health Law, Policy and Management, Boston University School of Public Health, Boston, MA 02118 USA; 208 Deparment of Veterans Affairs, Center for Practice Management Outcomes Research, Ann Arbor, MI 48105 USA; 209 Department of Psychiatry, University of Michigan, Ann Arbor, MI 48105 USA; 210 Psychiatric Research Institute, University of Arkansas for Medical Sciences, Little Rock, AR 72205 USA; 211 Department of Veterans Affairs, VA Central Arkansas Veterans Healthcare System, North Little Rock, AR 72114 USA; 212 Health Services Research and Development, VA Puget Sound Health Care System, Seattle, WA 98101 USA; 213 Department of Health Services, University of Washington, School of Public Health, Seattle, WA 98195 USA; 214 School of Medicine, University of Texas Health Science Center at San Antonio, San Antonio, TX 78229 USA; 215 Veterans Evidence-based Research Dissemination and Implementation Center, South Texas Veterans Health Care System, San Antonio, TX 78229 USA; 216 Psychiatry, University of Texas Health Science Center at San Antonio, San Antonio, TX 78229 USA; 217 Health Services Research and Development, VA Puget Sound Health Care System, Seattle, WA 98101 USA; 218 Department of Health Services, University of Washington, School of Public Health, Seattle, WA 98195 USA; 219 Center of Innovation for Veteran-Centered and Value-Driven Care, Department of Veterans Affairs, Seattle, WA 98108 USA; 220 Center for Healthcare Organization and Implementation Research (CHOIR), Department of Veterans Affairs, Bedford, MA 01730 USA; 221 School of Nursing, University of North Carolina at Chapel Hill, Chapel Hill, NC 27599 USA; 222 Counter Tools, Carrboro, NC 27510 USA; 223 Health Behavior, Gillings School of Global Public Health, Chapel Hill, NC 27599 USA; 224 Lineberger Comprehensive Cancer Center, University of North Carolina at Chapel Hill, Chapel Hill, NC 27599 USA; 225 Brown School, Washington University in St. Louis, Saint Louis, MO 63130 USA; 226 Prevention Research Center, Washington University in St. Louis, St. Louis, MO 63130 USA; 227 Institute for Public Health, Washington University, St. Louis, MO 63112 USA; 228 Department of Pediatrics, Tufts Medical Center, Boston, MA 02111 USA; 229 Institute for Health, Health Care Policy, and Aging Research, Rutgers University, New Brunswick, NJ 08901 USA; 230 Institute for Community Health, Malden, MA 02148 USA; 231 Center for Healthcare Organization and Implementation Research (CHOIR), US Department of Veterans Affairs, Bedford, MA 01730 USA; 232 Institute for Clinical Research and Health Policy Studies, Tufts Medical Center, Boston, MA 02111 USA; 233 Communication, Rutgers University, New Brunswick, NJ 08901 USA; 234 Hathaway-Sycamores Child and Family Services, Pasadena, CA 91105 USA; 235 Department of Psychology, University of Montana, Missoula, MT 59812 USA; 236 Department of Psychological and Brain Sciences, Indiana University, Bloomington, IN 47405 USA; 237 Health Policy & Management, University of North Carolina at Chapel Hill, Chapel Hill, NC 27599-7411 USA; 238 Department of Global Health, University of Washington, Seattle, WA 98195 USA; 239 Group Health Research Institute, Seattle, WA 98101 USA; 240 Health Policy & Management, University of North Carolina at Chapel Hill, Chapel Hill, NC 27599-7411 USA; 241 Department of Global Health, University of Washington, Seattle, WA 98195 USA; 242 Hathaway-Sycamores Child and Family Services, Pasadena, CA 91105 USA; 243 Department of Psychology, University of Montana, Missoula, MT 59812 USA; 244 Department of Psychological and Brain Sciences, Indiana University, Bloomington, IN 47405 USA; 245 Group Health Research Institute, Seattle, WA 98101 USA; 246 Department of Global Health, University of Washington, Seattle, WA 98195 USA; 247 Department of Psychological and Brain Sciences, Indiana University, Bloomington, IN 47405 USA; 248 Hathaway-Sycamores Child and Family Services, Pasadena, CA 91105 USA; 249 Department of Psychology, University of Montana, Missoula, MT 59812 USA; 250 Health Policy & Management, University of North Carolina at Chapel Hill, Chapel Hill, NC 27599-7411 USA; 251 Group Health Research Institute, Seattle, WA 98101 USA; 252 Stanford University, Menlo Park, CA 94025 USA; 253 Dissemination and Training Division, National Center for PTSD, Menlo Park, CA 94025 USA; 254 Department of Psychology, Ryerson University, Toronto, ON M4C1B5 Canada; 255 Family and Preventive Medicine, University of Arkansas for Medical Sciences, Little Rock, AR 72205 USA; 256 Department of Pharmacy Practice, University of Arkansas for Medical Sciences, Little Rock, AR 72205 USA; 257 Psychiatry & Behavioral Sciences, Medical University of South Carolina, Charleston, SC 29425 USA; 258 Psychiatry & Behavioral Sciences, National Crime Victims Research & Treatment Center, Charleston, SC 29425 USA; 259 Health Policy & Management, University of North Carolina at Chapel Hill, Gillings School of Global Public Health, Chapel Hill, NC 27599-7411 USA; 260 Centre for Practice-Changing Research, Ottawa Hospital Research Institute, Ottawa, ON K1H 8 L6 Canada; 261 Institute of Health & Society, Newcastle University, Newcastle, NE24AX UK; 262 School of Epidemiology, Public Health, and Preventive Medicine, University of Ottawa, Ottawa, ON K1H 8 L6 Canada; 263 QUERI, Department of Veterans Affairs, Greater Los Angeles Healthcare System, Los Angeles, CA 90073 USA; 264 Health Policy and Management, UCLA School of Public Health, Los Angeles, CA 90024 USA; 265 Care Coordination QUERI, VA Greater Los Angeles Healthcare System, Los Angeles, CA 90073 USA; 266 David Geffen School of Medicine, University of California Los Angeles, Los Angeles, CA 90024 USA; 267 Research and Evaluation, Kaiser Permanente Southern California, Pasadena, CA 91101 USA; 268 HSR&D, South Texas Veterans Health Care System, San Antonio, TX 78229 USA; 269 Hospital Medicine & Psychiatry, University of Texas Health Science Center at San Antonio, San Antonio, TX 78207 USA; 270 Center of Innovation for Complex Chronic Healthcare (CINCCH), Department of Veterans Affairs, Hines, IL 60141 USA; 271 ENRM Veterans Hospital, VA HSR&D Center for Healthcare Organization and Implementation Research (CHOIR), Bedford, MA 01730 USA; 272 Health Law Policy and Management, Boston University School of Public Health, Boston, MA 02215 USA; 273 Evaluating Patient-Centered Care (EPCC), VA HSR&D Center for Healthcare Organization and Implementation Research (CHOIR), Bedford, MA 01730 USA; 274 Center for Healthcare Organization and Implementation Research, VA HSR&D Center for Healthcare Organization and Implementation Research (CHOIR), Bedford, MA 01730 USA; 275 Department of Physical Medicine and Rehabilitation, Feinberg School of Medicine, Northwestern University, Chicago, IL 60611 USA; 276 Department of Preventive Medicine and Center for Healthcare Studies, Institute for Public Health and Medicine (IPHAM), Northwestern University, Chicago, IL 60611 USA; 277 Psychology, University of North Carolina Charlotte, Charlotte, NC 28223 USA; 278 Institute for Healthcare Improvement, Institute for Healthcare Improvement, Reading, PA 19606 USA; 279 Psychology, University of South Carolina, Columbia, SC 29208 USA; 280 Alliance Manchester Business School, University of Manchester, Manchester, M15 6 PB UK; 281 Learning Health Sciences, University of Michigan, Ann Arbor, MI 48109-5403 USA; 282 Center for Clinical Management Research, VA Ann Arbor Healthcare System, Ann Arbor, MI 48109 USA; 283 Division of Cancer Control and Population Sciences, National Cancer Institute, Bethesda, MD 20850 USA; 284 Office of Public Health Genomics, Centers for Disease Control and Prevention, Atlanta, GA 30333 USA; 285 Durham VA Medical Center, Health Services Research and Development, Durham, NC 27701 USA; 286 Department of Medicine, Duke University, Durham, NC 27705 USA; 287 Duke University, Durham, NC 27708 USA; 288 School of Nursing, Indiana University, Indianapolis, IN 46202 USA; 289 College of Pharmacy, University of Florida, Gainesville, FL 32610-0486 USA; 290 Department of Biomedical Informatics, Vanderbilt University, Nashville, TN 37203 USA; 291 School of Medicine, University of Maryland, Baltimore, MD 21201 USA; 292 Population Health Science and Policy, Icahn School of Medicine at Mount Sinai, New York, NY 10029 USA; 293 National Human Genome Research Institute NIH, Bethesda, MD 20852 USA; 294 Department of Medical and Molecular Genetics, Indiana University, Indianapolis, IN 46202 USA; 295 Center for Applied Genomics and Precision Medicine, Duke University, Durham, NC 27708 USA; 296 School of Medicine, Indiana University, Indianapolis, IN 46202 USA; 297 Health Services Research & Development, Durham VAMC, Durham, NC 27705 USA; 298 Center for Clinical Management Research, Veterans Affairs, Ann Arbor, MI 48113-0170 USA; 299 Department of Population Medicine, Harvard Medical School and Harvard Pilgrim Health Care Institute, Boston, MA 02215 USA; 300 Department of Population Medicine, Harvard Pilgrim Health Care, Boston, MA 02215 USA; 301 Medicine, University of Massachusetts Medical School, Worcester, MA 01605 USA; 302 Genomic Medicine Institute, Geisinger Health System, Danville, PA 17822 USA; 303 Behavioral Sciences and Health Education, Rollins School of Public Health, Emory University, Atlanta, GA 30322, USA; 304 Health Promotion and Behavioral Sciences, University of Texas School of Public Health, Houston, TX 77578 USA; 305 Center for Health Promotion and Prevention Research, University of Texas School of Public Health, Houston, TX 77030 USA; 306 Center for Child Community Health Research, Johns Hopkins School of Medicine, Baltimore, MD 21224 USA; 307 Department of Population, Family, and Reproductive Health, Johns Hopkins Bloomberg School of Public Health, Baltimore, MD 21224 USA; 308 Department of Mental Health, Johns Hopkins Bloomberg School of Public Health, Baltimore, MD 21205 USA; 309 Medicine, Johns Hopkins University, Baltimore, MD 21224 USA; 310 Division of Family Health Services, New Jersey Department of Health, Trenton, NJ 08625 USA; 311 Health Promotion and Behavioral Sciences, The University of Texas Health Science Center School of Public Health, Houston, TX 77030 USA; 312 NW Tribal Epidemiology Center, Northwest Portland Area Indian Health Board, Portland, OR 97201 USA; 313 Project Red Talon, Northwest Portland Area Indian Health Board, Portland, OR 97201 USA; 314 Health and Human Services, Inter Tribal Council of Arizona, Inc., Phoenix, AZ 85004 USA; 315 HIV/STD Prevention Program, Alaska Native Tribal Health Consortium, Anchorage, AK 99508 USA; 316 Nutrition, University of North Carolina, Chapel Hill, NC 27599 USA; 317 Center for Health Promotion and Disease Prevention, University of North Carolina, Chapel Hill, NC 27599-7426 USA; 318 Center for Community-based Research, Dana-Farber Cancer Institute, Boston, MA 02215 USA; 319 Population Health Research Program, Harvard Catalyst: The Harvard Clinical and Translational Science Center, Boston, MA 02115 USA; 320 City of Lawrence Mayor’s Health Task Force, Lawrence, MA 01840 USA; 321 Social and Behavioral Sciences, Harvard T.H. Chan School of Public Health, Boston, MA 02215 USA; 322 Nicotine Dependence Service, Centre for Addiction and Mental Health, Toronto, ON M5T1P7 Canada; 323 Dalla Lana School of Public Health, University of Toronto, Toronto, ON M5T3M7 Canada; 324 School of Social Work, University of Windsor, Windsor, ON N9A 0C5 Canada; 325 Addictions Program, Centre for Addiction and Mental Health, Toronto, ON M5T1P7 Canada; 326 Family and Community Medicine, University of Toronto, Toronto, ON M5G1V7 Canada; 327 Psychiatry, University of Toronto, Toronto, ON M5T1R8 Canada; 328 Implementation Science Team, Division of Cancer Control and Population Sciences, National Cancer Institute, Rockville, MD 20850 USA; 329 Public Health, Community Science, Acton, MA 01720 USA; 330 Social and Behavioral Sciences, Harvard T.H. Chan School of Public Health, Boston, MA 02115 USA; 331 Center for Community-based Research, Dana-Farber Cancer Institute, Boston, MA 02215 USA; 332 Division of General Internal Medicine, Massachusetts General Hospital, Boston, MA 02114 USA; 333 Population Health Research Program, Harvard Catalyst: The Harvard Clinical and Translational Science Center, Boston, MA 02115 USA; 334 Institute for Medicaid Innovation, Institute for Medicaid Innovation, Washington, DC 20036 USA; 335 Knowledge and Evaluation Research Unit, Mayo Clinic, Rochester, MN 55905 USA; 336 Health Sciences Research, Mayo Clinic, Rochester, MN 55905 USA; 337 SE MN Area Agency on Aging, Rochester, MN 55902 USA; 338 Mayo Medical School, Rochester, MN 55905 USA; 339 Health Care and Policy Research, Mayo Clinic, Rochester, MN 55905 USA; 340 Tufts University Friedman School of Nutrition Science and Policy, Boston, MA 02111 USA; 341 Social System Design Lab, Washington University Brown School of Social Work, St. Louis, MO 63130 USA; 342 ChildObesity180, Tufts University Friedman School of Nutrition Science and Policy, Boston, MA 02111 USA; 343 Nutrition and Food Sciences, University of Rhode Island, Kingston, RI 02881 USA; 344 Center on Social Dynamics and Policy, Brookings Institution, Washington, DC 20036 USA; 345 HealthMPowers, Norcross, GA 30092 USA; 346 Epidemiology, Rollins School of Public Health, Atlanta, GA 30322 USA; 347 Georgia Department of Public Health, Atlanta, GA 30303-3186 USA; 348 Epidemiology, Rollins School of Public Health, Atlanta, GA 30322 USA; 349 HealthMPowers, Norcross, GA 30092 USA; 350 Biostatistics and Bioinformatics, Rollins School of Public Health, Atlanta, GA 30322 USA; 351 Behavioral Science and Health Education, Rollins School of Public Health, Atlanta, GA 30322 USA; 352 Epidemiology, Rollins School of Public Health, Atlanta, GA 30322 USA; 353 HealthMPowers, Norcross, GA 30092 USA; 354 Georgia Department of Public Health, Atlanta, GA 30303-3186, USA; 355 Psychiatry, Massachusetts General Hospital, Chelsea, MA 2150 USA; 356 Psychology, UCLA, Los Angeles, CA 90095-1563 USA; 357 Massachusetts General Hospital, Chelsea, MA 02150 USA; 358 Wang Ambulatory Care Center, Boston, MA 02114-3117 USA; 359 Psychology, Ryerson University, Toronto, ON MBC 1B5 Canada; 360 Dissemination and Training Division, National Center for PTSD and Stanford University, Menlo Park, CA 94025 USA; 361 Department of Human Development and Family Studies, Michigan State University, East Lansing, MI 48824 USA; 362 Epidemiology & Health Promotion, New York University College of Dentistry and College of Global Public Health, New York, NY 10010 USA; 363 Community Health Projects, UNITED SIKHS, New York, NY 10036 USA; 364 Population Health, NYU School of Medicine, New York, NY 10016 USA; 365 Director, UNITED SIKHS, New York, NY 10036 USA; 366 Epidemiology and Biostatistics, Case Western Reserve University, Cleveland, OH 44106 USA; 367 Prevention Research Center for Healthy Neighborhoods, Case Western Reserve University, Cleveland, OH 44106 USA; 368 Human and Organizational Development, Vanderbilt University, Nashville, TN 37203 USA; 369 Health Sciences, Cleveland State University, Cleveland, OH 44115 USA; 370 Anthropology, Case Western Reserve University, Cleveland, OH 44116 USA; 371 Human Nutrition Foods and Exercise, Virginia Polytechnic Institute, Blacksburg, VA 24060 USA; 372 Translational Biology, Medicine, and Health, Virginia Polytechnic Institute, Blacksburg, VA 24060 USA; 373 Virginia Cooperative Extension, Blacksburg, VA 24060 USA; 374 Psychology, University of South Carolina, Columbia, SC 29208 USA; 375 Institute for Healthcare Improvement, Reading, PA 19606 USA; 376 Psychology, University of North Carolina-Charlotte, Charlotte, NC 28223 USA; 377 Gillings School of Public Health, University of North Carolina, Chapel Hill, NC 27599 USA; 378 Science Program, Kaiser Permanente Center for Health Research, Portland, OR 97227 USA; 379 Family Medicine, Oregon Health and Science University, Portland, OR 97202 USA; 380 Epic, OCHIN, Inc., Portland, OR 97201 USA; 381 Practice Based Research Network, OCHIN, Inc., Portland, OR 97207 USA; 382 Medicine, Cowlitz Family Health Center, Long View, WA 98632 USA; 383 Public Health, University of California, Irvine (UCI), Irvine, CA 92697 USA; 384 Communication, REAL Prevention LLC, Clifton, NJ 07013 USA; 385 Human Development, REAL Prevention LLC, New Jersey, NJ 07013 USA; 386 Communication Studies, Chapman University, Orange, CA 92866 USA; 387 Health Policy and Administration, Pennsylvania State University, University Park, PA 16820 USA; 388 Pediatrics, IUPUI, Indianapolis, IN 46202 USA; 389 University of Kansas Medical Center, KU Center for Telemedicine & Telehealth, Fairway, KS 66205 USA; 390 Hospice Services and Palliative Care of Northwest Kansas, Inc., Phillipsburg, KS 67661 USA; 391 University of Kansas Cancer Center, Westwood, KS 66205 USA; 392 Midwest Cancer Alliance, Fairway, KS 66205 USA; 393 Gastroenterology, The Permanente Medical Group, Walnut Creek, CA 94596 USA; 394 Regional Health Education, The Permanente Medical Group, Oakland, CA 94612 USA; 395 Quality and Operations Support, The Permanente Medical Group, Oakland, CA 94612 USA; 396 Addictions Medicine, Centre for Addiction and Mental Health, Toronto, ON M5S 3E3 Canada; 397 Nicotine Dependence Service, Centre for Addiction and Mental Health, Toronto, ON M5T1P7 Canada; 398 Addictions Program, Centre for Addiction and Mental Health, Toronto, ON M5T1P7 Canada; 399 Family and Community Medicine, University of Toronto, Toronto, ON M5G1V7 Canada; 400 Psychiatry, University of Toronto, Toronto, ON M5T1R8 Canada; 401 Dalla Lana School of Public Health, University of Toronto, Toronto, ON M5T3M7 Canada; 402 Pharmacology and Toxicology, University of Toronto, Toronto, ON M5T1P7 Canada; 403 Social, Prevention and Health Policy Research Department, Centre for Addiction and Mental Health, Toronto, ON M5S 2S1 Canada; 404 Emergency Medicine, Perelman School of Medicine at the University of Pennsylvania, University of Pennsylvania, Philadelphia, PA 19104 USA; 405 Department of Health Economics, Leonard Davis Institute of Health Economics, University of Pennsylvania, Philadelphia, PA 19104 USA; 406 Department of Healthcare Policy and Research, Weill Cornell Medical College, New York, NY 10065 USA; 407 Research, Quality, and Scientific Affairs, American Academy of Orthopaedic Surgeons (AAOS), Rosemont, IL 60118 USA; 408 Family Child Nursing, University of Washington, Seattle, WA 98112 USA; 409 Global Health, University of Washington, Seattle, WA 98112 USA; 410 Implementation Science, Health Alliance International, Seattle, WA 98112 USA; 411 Primary Health Care, Centre of Infectious Diseases in Zambia, Lusaka, 00000 Zambia; 412 Monitoring and Evaluation, Partners in Health, Butaro, 0000 Rwanda; 413 Mozambique, Health Alliance International, Maputo, 0000 Mozambique; 414 Implementation and Improvement Science Platform, Ariadne Labs, Boston, MA 02215 USA; 415 Global Health and Social Medicine, Harvard Medical School, Boston, MA 02115 USA; 416 Anesthesiology and Critical Care, Perelman School of Medicine, University of Pennsylvania, Philadelphia, PA 19104-4865 USA; 417 Psychiatry, University of Pennsylvania, Philadelphia, PA 19104 USA; 418 Leonard Davis Institute of Health Economics, University of Pennsylvania, Philadelphia, PA 19104 USA; 419 Family Medicine and Community Health, University of Pennsylvania, Philadelphia, PA 19104 USA; 420 Family and Social Medicine, Montefiore Medical Center, Bronx, NY 10467 USA; 421 Asthma UK Centre for Applied Research, Allergy and Respiratory Research Group, Usher Institute of Population Health Sciences and Informatics, University of Edinburgh, Edinburgh, EH8 9AG UK; 422 Research Institute, The Hospital for Sick Children, Toronto, ON M5G 1X8 Canada; 423 Division of Emergency Medicine, Washington University, St. Louis, MO 63110 USA; 424 Centre for Primary Care and Public Health, Queen Mary University of London, London, E1 2AT UK; 425 Primary Care Research Unit of Bizkaia, Basque Health Service, Bilbao, Spain; 426 School of Healthcare Sciences, Bangor University, Bangor, Gwynedd LL57 2EF UK; 427 Research Department of Primary Care and Population Health, University College London, London, NW3 2PF UK; 428 Centre for Primary Care and Public Health, Barts and The London School of Medicine and Dentistry, London, E1 2AT UK; 429 Centre for Population Health Sciences, Brigham and Women’s Hospital, Boston, MA 02120-1613 USA; 430 Research and Evaluation, Kaiser Permanente Southern Calif / US Dept of Veterans Affairs, Pasadena, CA 91101 USA; 431 Division of Health and Social Care Research, King’s College London, London, WC2R 2LS UK; 432 Centre for Technology Enabled Health Research, Coventry University, Coventry, CV1 5FB UK; 433 Biomedical Informatics and Medical Education, University of Washington, Seattle, WA 98195 USA; 434 Institute of Public Health Genetics, University of Washington, Seattle, WA 98195 USA; 435 Bioethics and Humanities, University of Washington, Seattle, WA 98195 USA; 436 Department of Health Services, University of Washington, Seattle, WA 98105 USA; 437 Department of Biostatistics, School of Public Health, University of Washington, Seattle, WA 98199 USA; 438 Health Promotion Research Center, University of Washington, Seattle, WA 98105 USA; 439 Health Services Research and Development, VA Puget Sound Health Care System, Seattle, WA 98101 USA; 440 VA Health Services Center of Innovation for Veteran-Centered and Value-Driven Care, Department of Veterans Affairs, Seattle, WA 98108 USA; 441 Epidemiology, University of Washington, Seattle, WA 98195 USA; 442 American Cancer Society, Preventive Health Partnership, Atlanta, GA 30303 USA; 443 Department of Sociomedical Sciences, Columbia’s Mailman School of Public Health, New York, NY 10032 USA; 444 Oncological Sciences, Mount Sinai School of Medicine, New York, NY 10029 USA; 445 Division of Cancer Prevention and Population Sciences, Roswell Park Cancer Institute, Buffalo, NY 14263 USA; 446 MacColl Center for Health Care Innovation, Group Health Research Institute, Seattle, WA 98101 USA; 447 Department of Family Medicine, University of Washington, Institute of Translational Health Sciences, Seattle, WA 98195 USA; 448 Center for the Study of Healthcare Innovation, Implementation & Policy, VA Greater Los Angeles Healthcare System, Sepulveda, CA 91343 USA; 449 HSR&D, Dept. of Veterans Affairs, Tampa, FL 33637 USA; 450 Department of Veterans Affairs, Gainesville, FL 32608 USA; 451 OIA, VHA, Washington, DC 20240 USA; 452 OIA, Department of Veterans Affairs, Kansas City, MO 64105 USA; 453 CHE, Dept. of Veterans Affairs, Sioux City, IA 87108 USA; 454 HSR&D, VHA, Los Angeles, CA 91343 USA; 455 HSR&D Center for the Study of Healthcare Innovation, Implementation, and Policy, VA Greater Los Angeles Healthcare System, Los Angeles, CA 90073 USA; 456 GEC, Dept. of Veterans Affairs, Los Angeles, CA 90073 USA; 457 David Geffen School of Medicine at UCLA, Los Angeles, CA USA; 458 Center for Healthcare Organization and Implementation Research (CHOIR), VA Health Services Research & Development, Department of Veterans Affairs, Bedford, MA 01730 USA; 459 Department of Health Law, Policy and Management, Boston University School of Public Health, Boston, MA 02118 USA; 460 Department of Veterans Affairs, Los Angeles, CA 90073 USA; 461 Department of Psychiatry, UCLA, Los Angeles, CA 90073 USA; 462 VA Quality Enhancement Research Initiative (QUERI) for Team-Based Behavioral Health, North Little Rock, AR 72114 USA; 463 Department of Psychiatry, University of Massachusetts Medical School, Worcester, MA 01655 USA; 464 Psychiatry, UC San Diego, La Jolla, CA 92093 USA; 465 Department of Medicine, Hematology/Oncology / Department of Medical Ethics and Health Policy, The University of Pennsylvania, Philadelphia, PA 19104 USA; 466 Center for Clinical Cancer Genetics, The University of Chicago, Chicago, IL 60637 USA; 467 Biostatistics and Bioinformatics Facility, The Fox Chase Cancer Center, Philadelphia, PA 19111 USA; 468 Department of Medicine, Division of Hematology-Oncology, The University of Pennsylvania, Abramson Cancer Center, Philadelphia, PA 19104 USA; 469 Clinical Genetics / Gastrointestinal Risk Assessment, The Fox Chase Cancer Center, Philadelphia, PA 19111 USA; 470 Department of Clinical Genetics, The Fox Chase Cancer Center, Philadelphia, PA 19111 USA; 471 Research Institute, The Children’s Hospital of Philadelphia, Philadelphia, PA 19104 USA; 472 Population Science, Fox Chase Cancer Center, Philadelphia, PA 19015 USA; 473 Hematology and Medical Oncology, MD Anderson Cancer Center at Cooper, Camden, NJ 08043 USA; 474 Internal Medicine, John H. Stroger, Jr. Hospital, Chicago, IL 60612 USA; 475 Department of Medicine, The University of Chicago, Chicago, IL 60637 USA; 476 Breast and Cervical Cancer Screening Program, John H. Stroger, Jr. Hospital, Chicago, IL 60612 USA; 477 Cancer Genetics Program, MD Anderson Cancer Center at Cooper, Camden, NJ 08043 USA; 478 Cancer Screening Program, John H. Stroger, Jr. Hospital, Chicago, IL 60612 USA; 479 Comprehensive Cancer Risk and Prevention Clinic, The University of Chicago, Chicago, IL 60637 USA; 480 HSR&D, Richard L. Roudebush VA Medical Center, Indianapolis, IN 46202 USA; 481 HSR&D, VA Stroke Queri Center, Indianapolis, IN 46239 USA; 482 HSR&D/QUERI, Roudebush VA Medical Center, Indianapolis, IN 46202 USA; 483 Office of Disease Prevention, National Institutes of Health, Bethesda, MD 20892 USA; 484 Behavioral Health Research Center of the Southwest, Pacific Institute for Research and Evaluation, Albuquerque, NM 87102 USA; 485 Division of Research, Kaiser Permanente, Oakland, CA 94612 USA; 486 Health Care Effectiveness, Kaiser Permanente Division of Research, Oakland, CA 94612 USA; 487 Decision Support, Kaiser Permanente Northern California, Oakland, CA, 94612 USA

## A1 Introduction

### David Chambers^1^, Lisa Simpson^2^, Gila Neta^1^

#### ^1^Division of Cancer Control and Population Sciences, National Cancer Institute, Rockville, MD, 20850, USA; ^2^AcademyHealth, Washington, DC, 20009, USA

At a time of significant upheaval in American health policy, maintaining a focus on a “North Star” is critical. For implementation science, this star is the knowledge base on how to optimally disseminate evidence related to health and health care, how to implement interventions to improve care within the many settings where people receive health care and make health-related decisions, and how to improve the health of the global population. To that end, the end of 2016 brought over 1100 engaged and activated “disciples of D & I” to Washington, DC for the 9^th^ Annual Conference on the Science of Dissemination and Implementation in Health. Once again, the accompanying abstracts in this issue demonstrate the breadth, depth and vigor of this continually expanding and evolving subset of health research. During three dynamic plenaries with rows and rows of filled seats and packed concurrent sessions presenters and attendees shared findings, raised methodologic and other challenges, and discussed future priorities, trends, and next steps for this community of research.

For the third year in a row, we were buoyed by a strong partnership, co-led by AcademyHealth and the National Institutes of Health (NIH), with co-sponsorship from others committed to implementation science: the Agency for Healthcare Research and Quality (AHRQ), the Patient Centered Outcomes Research Institute (PCORI), the Robert Wood Johnson Foundation (RWJF), and the US Department of Veterans Affairs (VA). The multidisciplinary program planning committee informed the development of the key themes for the conference, identified the plenary sessions topics and speakers, established track leads to manage the review process for concurrent panels, papers, and posters, and convened a scientific advisory panel to advise on the overall conference, thus ensuring a robust, inclusive, and rigorous process.

Together, the opening keynote address and the three plenary panel sessions set a tone of innovation and dialogue, raised critical issues, surfaced different perspectives, and ensured that follow on lunchtime and hallway discussions delved deeper into thorny challenges facing the field. Roy Rosin, Chief Innovation Officer for the University of Pennsylvania’s Perelman School of Medicine, introduced the audience to a range of methods for rapid testing, innovation in healthcare delivery, and lessons learned from other industries to maximize potential of new practices to be scaled-up. Each of the three plenary panels presented a general discussion on a high priority challenge for dissemination and implementation (D & I) research. A panel on the balance between intervention and implementation fidelity and local adaptation touched on the very real dynamic that is playing out in communities across this country as policy and payment changes are driving providers and others to seek new ways to solve the challenges in their particular contexts. A panel on the longer-term decisions around sustainment or de-implementation of interventions could not be more timely given the “improvement fatigue” of some systems and providers and the very real limits on providers’ time and focus. Too often, the imperative is to “do more”; much more attention needs to be about stopping what is not working, particularly in light of estimates that 30 percent of care provided is either unnecessary, of low value or wasteful (Institute of Medicine, 2013). The third plenary panel brought different perspectives on the enduring and evolving challenges in the dissemination of evidence and evidence-based practices as well as the opportunities emerging from innovations in the digital health sector. The plenary sessions were complemented by facilitated lunchtime discussions on these topics, as well as additional research priorities, which enabled more in-depth discussions, additional question and answer time, and brainstorming of future directions. Synopses of the lunchtime discussions are included in this supplement.

The concurrent sessions were once again organized by tracks. Last year’s tracks—Behavioral Health, Big Data and Technology for Dissemination and Implementation Research, Clinical Care Settings, Global Dissemination and Implementation, Promoting Health Equity and Eliminating Disparities, Health Policy Dissemination and Implementation, Prevention and Public Health, and Models, Measures and Methods—were maintained, and a new track on Precision Medicine was added, built upon the significant interest that emerged from last year’s plenary and subsequent discussions at NIH, National Academy of Medicine, and beyond. The tracks again enabled conference participants to follow a consistent theme across the multiple sessions of the conference and to better group thematically the individual papers and posters submitted by the conference participants. This supplement also is organized by these track themes.

The call for abstracts, including individual paper presentations, individual posters and panel presentations, resulted in 601 submissions, spread across the nine thematic tracks. Over one hundred reviewers from multiple disciplines, sectors, settings and career stage devoted their time to ensuring a comprehensive and expert review, and reviews were conducted within each track and coordinated by the track leads. For the final program, 19 oral abstract sessions, 9 panels, and 334 posters were presented over the two-day meeting, in addition to a “poster slam”. Slides for the oral presentations and panels (with the agreement of the authors) were posted on the conference website (https://academyhealth.confex.com/academyhealth/2016di/meetingapp.cgi/Home/0) and all abstracts were included on the conference webapp (https://academyhealth.confex.com/academyhealth/2016di/meetingapp.cgi). New this year was a presentation format that combined the efficiency of posters with the benefits of oral presentation—the poster slam. Eighteen of the top scoring posters were selected for this format. This new format enabled the lead authors of the top scoring posters to present their key findings in three minutes each, forcing clarity of communication on the vital message of their research. This skill of the “elevator” pitch is often not well developed amongst researchers.

This supplement has compiled the abstracts for presented papers, panel sessions, and lunchtime discussions from the 9th Annual Meeting on the Science of Dissemination and Implementation in Health: Mapping the Complexity and Dynamism of the Field. We are pleased to have the combined proceedings from the conference together in one volume once again, and look forward to the 10th Annual meeting, scheduled for December 4-5, 2017 in Arlington, VA. We look forward to next year’s conference which will also be the 10th anniversary of this convening, marking just how much implementation science has grown over the last decade.

## D1 Balancing adaptation and fidelity: Exploring the continuum

### Ulrica von Thiele Schwarz^1^, Antoinette Percy-Laurry^2^, Gregory A. Aarons^3^

#### ^1^Department of Learning, Informatics, Management, and Ethics, Karolinska Institutet, Stockholm, 17177, Sweden; ^2^Division of Cancer Control and Population Sciences, National Cancer Institute, Rockville, MD, 20850, USA; ^3^Department of Psychiatry, University of California San Diego, San Diego, CA, 92093, USA

Efforts to implement evidence-based interventions (EBIs) must address the challenges of remaining faithful to the original design of an intervention (i.e., fidelity) while considering whether and how adapt to local populations, contexts, or delivery models. Similarly, there may also be a need for adaptation of implementation strategies and adaptation of systems and organizations in which EBIs are being implemented. It is increasingly recognized that adaptation processes go beyond simple either/or decisions in considering when, how, and why adaptations are necessary. This session provided an opportunity for participants to address the challenges of, and strategies for, studying and achieving adaptation without sacrificing meaningful fidelity. There were approximately 100 participants in this 90 minute session that included small group discussions and large group interactive discussions facilitated by the authors.

The key themes identified included the importance of determining how much adaptation indicates a significant departure from the EBI, types of EBI or contextual adaptations that might be needed for patients with comorbidities, how adaptations can be tailored within complex health systems, and optimal methods and measures for assessing fidelity and adaptation. Other central areas of focus were how to scale adaptation across health systems, identifying contextual influences on adaptation, and the tension in moving from adaptation to innovation while avoiding drift that may compromise EBI or implementation strategy effectiveness.

Recommendations included examining the degree to which adaptation is occurring, discriminating between intentional vs. unintentional adaptation, using metrics to determine when drift occurs, and having a plan to facilitate and monitor appropriate adaptation and drift.

A call was made for researchers to put more emphasis on identifying core EBI and implementation strategy components, and it was highlighted that in order to manage fidelity and adaptations, both the EBI and the clinical environment needs to be understood. This calls for collaborative approaches between researchers and practitioners. Also, there is a need to consider the outer system context and inner organizational context factors that can constrain or allow adaptation, as well as prioritizing scaling-up and spreading EBIs that are robust enough to survive some adaptation. Another recommendation was to use technology to assess fidelity in real-time and to be sure that fidelity assessment and source (e.g., clinician report, patient report, observation, etc.) is reliable and valid. Lastly, the importance of how adaptations or reinvention impact quality of care and outcomes was voiced. In conclusion, the discussion highlighted that often, the question is not a matter of either fidelity *or* adaptation, but rather fidelity *and* adaptation.

## D2 Novel directions in dissemination research

### Gila Neta^1^, Ross Brownson^2^

#### ^1^Division of Cancer Control and Population Sciences, National Cancer Institute, Rockville, MD, 20850, USA; ^2^George Warren Brown School of Social Work, Washington University in St. Louis, St. Louis, MO, 63130, USA

Within funded health-related research, far greater emphasis seems to be placed on implementation of evidence-based practices than on effective dissemination processes to get evidence and evidence-based interventions spread to target audiences. With great advances in communications technology and new sources of data, this lunch discussion forum brought together over 150 conference attendees to discuss a broad range of challenges, share experiences, and explore future directions for dissemination research.

Participants began the discussion by distinguishing features of dissemination research as opposed to implementation research (i.e., directive spread, less active, communicating vs. adopting, not setting-specific) and noting that dissemination is not spontaneous and not effective when passive. Aspects of successful dissemination were highlighted, including stakeholder engagement, team science, community based participatory research, and coalition building. Participants also explored what makes research innovative, reflecting on the keynote by Roy Rosin and highlighting the value of rethinking the problem. Other aspects of innovation included: solving a problem that has not been solved before, addressing a problem in a new context, applying technology in new ways, exploring the significance of the changing environment, engaging a new sector or partners in addressing a problem, applying a theory from an outside sector, transferring technology in new ways, and rethinking strategies needed for scale up. Strategies to identify novel topics were explored, including identifying evidence gaps, using knowledge brokers, engaging partnerships, spending time with practitioners, and concept mapping.

Examples of innovative and scalable research opportunities were discussed, including in primary prevention and the Affordable Care Act, the integration of public health, literacy, tracking behavior in real time, social determinants of health, and value-based care and how to incorporate patient perspectives. Participants also emphasized research challenges, including in finding funding for cross cutting health issues, review processes, and data credibility particularly in the eyes of providers. And future directions were highlighted, including in policy dissemination, examination of ways in which evidence is moving, longitudinal evaluation of the impact of dissemination research, and understanding mechanism through which partnerships work and how best to measure that.

The discussion concluded with brief presentations about resources in dissemination research, including from Maureen Dobbins of the National Collaborative Centre for Methods and Tools who highlighted their capacity building activities, support for change initiatives, and mechanisms to disseminate reviews. A brief overview of resources at the National Cancer Institute in dissemination research was also highlighted and can be found at https://cancercontrol.cancer.gov/is/.

## D3 Planning for the long-term: considering sustainment

### Amanda Vogel^1^, Shannon Wiltsey Stirman^2^

#### ^1^Center for Global Health, National Cancer Institute, Rockville, MD, 20850, USA; ^2^Dissemination and Training Division, VA National Center for PTSD, Palo Alto, CA, 94305, USA

The last ten years have shown increasing interest in sustainment of interventions in population health and healthcare. In this lunchtime discussion forum, participants from academia, community-based organizations, and research corporations discussed challenges, strategies, and goals in sustainment research.

A key theme of the conversation was challenges and strategies around funding for sustainment research. Participants discussed the limitations of the five-year research grant to cover both implementation and sustainment research. It was suggested that this was enough time to include both types of research only if an intervention could be implemented in full in year 1 (e.g., a minor change in EMRs). The group discussed the related challenge of being unsure at what point to shift from implementation research to sustainment research. Participants shared related strategies such as refining and implementing interventions in early waves of funding, and studying adaptation and/or sustainment in later waves of funding. A number of persons discussed the opportunities created by observational approaches to sustainment research. The importance of theory-driven research was also noted.

Another key theme of the conversation was the importance of adaptation for effective dissemination and sustainment. The group discussed a range of challenges related to effectively adapting interventions to suit a wide range of new contexts, including: balancing fidelity and adaptation in diverse contexts, adapting complex multi-part interventions to limited resource settings, and successfully implementing and sustaining an intervention in the absence of its original champions. The group highlighted related needs in D&I science, including: a need for intervention designers to identify and specify the core of the intervention versus the adaptable periphery; a need to document how peripheral aspects are adapted as context for reporting results; a need to capture multilevel influences on sustainability; the value of implementation tools and playbooks to support effective adaptation without reinventing the wheel; and the use of SMART designs, A/B designs, and observational approaches to compare the effectiveness of various adaptations in different populations/patient groups/contexts. The conversation also highlighted the need to understand how to routinize new interventions so they become part of “the way things are done” (but not to the extent that the interventions become entrenched and difficult to replace if more effective practices are identified), with an emphasis on training individuals in multiple implementing and leadership roles to facilitate routinization.

Overall, the group was highly engaged and committed to this area of D&I science. They framed sustainment research as an essential component of D&I science, and necessary to generate the data on long-term costs, population health and patient outcomes that are essential to public health and healthcare decision making. They believed these factors, along with interest in ROI and efficiency, were powerful reasons for funding agencies to invest in sustainment research.

## D4 Addressing education and training needs in implementation science

### Kenneth Sherr^1^, Rachel Sturke^2^

#### ^1^Department of Global Health, University of Washington, Seattle, WA, 98105, USA; ^2^Center for Global Health Studies, Fogarty International Center, National Institutes of Health, Bethesda, MD, 20912, USA

Over the past decade, there has been remarkable increased interest in the field of implementation science, with commensurate increases in demand for training. A number of programs and resources have been created to address this need. In this discussion forum, participants representing researchers and practitioners from academia, industry, community-based organizations, and research funders shared and discussed existing training and education opportunities in implementation science. Participants discussed barriers and facilitators to establishing and accessing training programs, and priorities for training in implementation science.

Multiple themes emerged through the discussion. Participants described an array of existing training opportunities in implementation science globally and domestically, ranging from short courses to long-term degree programs, and targeting professionals in practice and/or researchers. Despite increases in training opportunities, demand continues to outpace supply, especially for low- and middle-income country researchers. Participants identified a number of constraints in implementation science training. The field lacks a standardized taxonomy for implementation science, including its definition and methodologic components. Furthermore, training competencies are not well formed, nor differentiated for multiple end users (e.g. practitioners vs. researchers, domestic vs. global implementation scientists, undergraduate vs. graduate/doctoral trainees), which impedes assessment of training programs success. Participants noted that there is a lack of critical mass of expertise in implementation science, including insufficient mentors and support for mentors, which combined with limited funding opportunities to support training, constrains efforts to build a broad base for the field. Finally, participants noted the need to educate the value of implementation science among practitioner organizations, grant reviewers and journal reviewers.

Participants identified priorities to address implementation science training gaps. First, a crosswalk of existing training programs to catalog competencies for different end users, metrics for these competencies, and core methodologies and tools covered in these training programs could improve consensus and identify specific gaps to inform future training development. Efforts are needed to engage practitioner organizations to enhance training programs to meet their priorities, and generate support for training in implementation science; a clear understanding of return on investment for implementation science, perhaps illustrated through a set of case studies would support these efforts. Further engagement with disciplines outside of the health sciences would support infusing implementation science into other training programs. Finally, participants noted that diverse funding for implementation science training is needed to match the needs of different end users, including funding to support dedicated mentorship to build a critical mass in implementation science expertise.

## D5 De-implementation

### Wynne E. Norton^1^, Allyson Varley^2^, David Chambers^1^

#### ^1^Division of Cancer Control and Population Sciences, National Cancer Institute, Rockville, MD, 20850, USA; ^2^School of Public Health, University of Alabama at Birmingham, Birmingham, AL, 35243, USA

De-implementation—reducing or stopping the use of ineffective, harmful, low-value, and/or unproven interventions, practices, and programs—is an important but understudied area of research. To advance the field, we need to develop and test frameworks, methods, measures, outcomes and strategies that address issues specific to de-implementation issues. The purpose of the forum was to discuss these issues, raise awareness of and interest in the need for advancing the science, and identify next steps for moving in such a direction.

During the forum, researchers, funders, and practitioners with various health care and public health perspectives identified key questions and issues in de-implementation, including: terminology, definitions, multi-level factors, frameworks, challenges in conducting research on de-implementation, and priorities for advancing the field. Common questions focused on how to incentivize de-implementation among multi-level stakeholders, including patients, providers, care teams, organizations, systems, and policies. Other challenges included mapping terms and processes, identifying and testing appropriate frameworks, and utilizing diverse methodologies (e.g., direct observation) to understand de-implementation processes, and testing financial and other de-implementation strategies. Participants discussed ways to capitalize on ongoing initiatives to reduce overuse or inappropriate care (e.g., Choosing Wisely; http://www.choosingwisely.org/) to study de-implementation. Participants also noted commonly-used terms broadly used to describe the field of de-implementation (e.g., reassessment, disinvestment, de-adoption, and decreased use), and the need for standardized definitions, operationalization, and validated measures. Finally, processes involved in de-implementation were perceived as similar to but distinct from those involved in implementation, warranting novel conceptualizations, outcomes, research designs, and practical considerations.

Consistent with the need to advance this emerging discipline, recommendations from attendees to advance the field were numerous and multi-pronged. To begin, participants suggested organizing stakeholder meetings with relevant health researchers, practitioners, funders, payers, and policymakers to discuss definitions, conceptualizations, and prioritize next steps. Participants also encouraged efforts to raise awareness of the field, including publishing data-driven and perspective pieces on terminology, standards for warranting de-implementation, outcomes, and incentives for de-implementation. Finally, participants suggested ways in which the field could use natural experiments to study policy changes aimed at reducing overuse as well as collecting data on de-implementation from ongoing implementation studies.

## D6 Designing for dissemination and implementation

### Cynthia Vinson^1^, Lisa Klesges^1,2^, Suzanne Heurtin-Roberts^1^

#### ^1^Division of Cancer Control and Population Sciences, National Cancer Institute, Rockville, MD, 20850, USA; ^2^School of Public Health, University of Memphis, Memphis, TN, 38152, USA

Behavioral researchers have evidence of effective health interventions that work, but this evidence is not widely used in practice. Programs to disseminate effective interventions exist (Small Business Innovative Research Program (SBIR), NSF I-Corps program, etc.) to help move biomedical research into practice, but there is limited assistance for behavioral researchers to move their interventions into practice. This lunch discussion forum provided participants the opportunity to explore how the field can better support efforts to design interventions with dissemination and implementation in mind.

The forum began with brief statements by NCI leaders regarding the concept of designing for implementation. Several considerations in designing for implementation were discussed including giving serious attention to ease of future implementation in practice, considering the range of contexts in which an intervention is likely to be implemented, as does implementation feasibility. Researchers, practitioners and funders engaged in discussions that raised a number of issues and lessons learned from their own experience.

Concern about minimizing the intrusiveness and influence on implementation sites was raised, since the trial of an implementation is supposed to occur in environments as they exist. Significant modification of an implementation site by research activities can diminish the validity and utility of results. It can obscure findings regarding the resources generally needed and available to implement an intervention.

Hybrid designs were discussed as a means to allow the development of an intervention in the same study that implementation research questions were asked. Further discussion focused on which designs might be appropriate at which stage of intervention development.

The importance of researcher, practitioner, and other stakeholder collaboration was emphasized. The utility of a participatory approach to the design of an implementable intervention was noted.

Career issues were discussed, with special concern for early researchers. Developing effective practices doesn’t necessarily build academic careers in the traditional model. Novel efficacious interventions are rewarded in publications and further research funding which are necessary for success in academic careers. The need for publishable intervention outcomes as well as successful implementation outcomes and strategies are challenging for those seeking tenure and career advancement.

The discussion was an interactive and supportive one. As participants asked questions and raised issues, other participants engaged to respond to questions with accountings of previous experiences and lessons learned.

## D7 Learning from improvement

### M. Rashad Massoud, Leighann Kimble

#### USAID Applying Science to Strengthen and Improve Systems (ASSIST) Project, Bethesda, Maryland 20814, USA

The purpose for the lunch discussion on “Learning from Improvement” was to report out on and discuss insights from the Salzburg, Global Seminar Session 565, “Better Health Care: How do we learn about improvement?”, which was convened from July 10th – 15th, 2016. During the lunch discussion a panel of participants from the seminar shared insights from the session as well as next steps.

The session is designed to discuss the need for research methods, such as mixed methods in order to address the complex issues posed by health care delivery. Understanding that the contextual nature of health care delivery in the real-world setting does not easily allow for replicability in research, the Salzburg Global Seminar discussed issues such as:

- Can we attribute the improvements we are measuring to the changes we are testing and implementing?
- How do we know that no other factors are influencing the results?
- If other factors are also affecting the results, how do we know what part is attributable to the changes we are making?
- Why did the changes which yielded improvements work, and how?
- How can we incorporate the effects of local context into improvements?
- How should we design improvement efforts to answer different learning objectives?
- How do we optimize data collection that simultaneously serves to drive quality improvement, inform evaluation efforts, and fulfil performance reporting requirements?

As a result of deliberating on these questions during the seminar, a framework was created to tackle these issues and encourage a “marriage” between implementation and research and evaluation. The framework describes that all improvement and evaluation must start with an aim specific detailing what is to be done and for whom. The aim is interwoven with a “theory of change”. The theory of change itself is closely related to “what” is to be done and “how” it is to be done in the improvement. Each of these factors, “what”, “how”, and the “theory of change” are related to context. In consideration of all of these complex factors, evaluation design and methods must be selected and integrated. In this way, evaluation design must occur alongside improvement, not after an improvement initiative is conducted. Such a change will allow for both better improvement design to allow for better learning and better evaluation design to inform implementation.

After insights from the seminar were shared regarding these issues, interaction, questions, and comments from participants was encouraged through group discussion.

## Behavioral Health

### S1 Large-scale implementation of collaborative care management for depression and diabetes and/or cardiovascular disease

#### Arne Beck^1^, Claire Neely^2^, Jennifer Boggs^1^

##### ^1^ Institute for Health Research, Kaiser Permanente, Denver, CO, 80231, USA; ^2^ICSI, Institute for Clinical Systems Improvement, Bloomington, MN, 55425, USA

###### **Correspondence:** Claire Neely

**Background**

The effectiveness of collaborative care management for patients with depression and comorbid chronic medical conditions is influenced by numerous implementation factors. The objective of this study was to describe the variation in implementation across medical groups of the Care of Mental, Physical, and Substance-use Syndromes (COMPASS) initiative, a large-scale implementation of an evidence-based collaborative care management model to improve health outcomes in patients with depression and diabetes and/or cardiovascular disease.

**Methods**

Ten health care systems participated in the COMPASS initiative, representing 18 medical groups with over 172 clinics across eight states, and 3,854 patients. Eligible patients had active depression (PHQ9 > =10) and one of the following poorly controlled medical conditions: diabetes mellitus with a HgbA1c > 8% or systolic blood pressure (SBP) > 145; and/or cardiovascular disease with (SBP > 145 [SBP > 165 for patients over 65]). COMPASS entailed 1) a clearly defined care management process, a care team that included a consulting physician and psychiatrist, and weekly systematic case reviews focusing on treat-to-target guidelines; 2) A registry to track patients for follow-up contacts; and 3) monitoring of hospital and emergency department utilization. Descriptive data on COMPASS implementation were obtained from annual site visit reports and supplemental site surveys. Site visit reports were analyzed with Atlas.ti software to identify emergent themes regarding implementation. The Consolidated Framework for Implementation Research (CFIR) was used to generalize themes to broader implementation constructs.

**Findings**

Nine specific implementation themes were identified that related to six main CFIR constructs: 1) Length of patient enrollment in COMPASS and 2) registry use (CFIR intervention characteristics); 3) patients’ social needs and 4) challenges to health systems’ organizational environments (CFIR outer setting), 5) primary care physician engagement and 6) experiences with care coordination (CFIR inner setting); 7) care manager characteristics (CFIR characteristics of individuals); and 8) COMPASS care team dynamics and 9) quality improvement and outcomes monitoring reports (CFIR process).

**Implications for D&I Research**

Substantial variation across COMPASS medical groups was observed in the emergent implementation themes. Understanding such variation may provide important data with which to increase successful large-scale dissemination of similar models.

**Primary Funding Source**

Centers for Medicare and Medicaid Services - The project described was supported by Grant Number 1C1CMS331048-01-00 from the Department of Health and Human Services, Centers for Medicare & Medicaid Services

### S2 Cost analysis of the collaborative care model for behavioral health in an urban, African-American, Medicare population

#### Carmel Nichols, Wen Wan, Erin Staab, Neda Laiteerapong

##### Medicine, University of Chicago, Chicago, IL, 60637, USA

###### **Correspondence:** Carmel Nichols

**Background**

The Collaborative Care Model (CCM) for behavioral health has been studied in more than 70 randomized controlled trials and has been shown to be more cost-effective than usual care in the primary care setting. However, it has not been evaluated in an urban, predominately African-American, Medicare population. We evaluated the change in inpatient and outpatient provider costs before and after implementation of the CCM in this population.

**Methods**

The Medicare Advantage Program (MAP) CCM program for Behavioral Health was implemented in January 2016 at the primary care clinic of an urban academic medical center. The MAP CCM program involved routine and targeted mental health screening, outreach, and comprehensive care management. We performed a retrospective analysis of the total costs of patients who were continually enrolled in Medicare Advantage from July 2015 to June 2016. Changes in total cost before and after the start date of the CCM were compared using a Wilcoxon signed rank test, as well as changes in emergency room, inpatient and outpatient costs.

**Findings**

Of the 762 patients in MAP, 460 patients were continuously enrolled during the study period. Median (range) age was 69 years (29-95), 67% were female and over 85% were African-American. There was a significant decrease in median health care costs after (vs. before) the CCM implementation (difference = -$1,899, interquartile range (IQR) (-11,921, 5193), p = 0.005), mostly due to a decrease in outpatient (difference = -$946, IQR (-8283, 4501), p = 0.01), and emergency room (p = 0.002) costs. There was also a decrease, although not significant, in inpatient costs (p = 0.26).

**Implications for D&I Research**

These results provide evidence of potential cost-saving attributable to the Collaborative Care Model for Behavioral Health in an urban, predominately African-American, Medicare population. Future studies will include a more detailed evaluation of the CCM program in order to evaluate the cost-effectiveness of the program in this population, both for patients with depression and for those with depression and diabetes.

### S3 Barriers and facilitators to implementing the New York state collaborative care initiative for depression in academic primary care settings: using a theoretical framework to inform policy

#### Nathalie Moise^1^, Ravi Shah^2^, Susan Essock^2^, Margaret Handley^3^, Amy Jones^4^, Jay Carruthers^4^, Karina Davidson^1^, Lauren Peccoralo^5^, Lloyd Sederer^4^

##### ^1^Medicine, Columbia University Medical Center, New York, NY, 10032, USA; ^2^Psychiatry, Columbia University, New York, NY, 10032, USA; ^3^General Internal Medicine, Epidemiology and Biostatistics, University of California San Francisco, San Francisco, CA, 94110, USA; ^4^New York State, Office of Mental Health, New York, NY, 10001, USA; ^5^Medicine, Icahn School of Medicine at Mount Sinai, New York, NY, 10029, USA

###### **Correspondence:** Nathalie Moise

**Background**

In 2012, the New York State (NYS) Office of Mental Health, in partnership with the NYS Department of Health, implemented Collaborative Care (CC) for depression in 19 academic medical centers (32 clinics serving 1 million patients). We aimed to assess barriers and facilitators to its implementation and to use a theoretical framework to propose corresponding policy and behavioral change interventions for future successful spread of CC.

**Methods**

Of 19 centers, 17 agreed to be interviewed. We completed 30 semi-structured interviews (6 psychiatrists, 8 clinic administrators, 8 primary care physicians, and 8 care managers) representing 8 healthcare systems before reaching saturation. We conducted a hybrid process of both inductive and deductive thematic analysis (NVIVO 11.1), before using the *Capability, Opportunity and Motivation-Behavior (COM-B)* model to characterize the themes and to propose related policy recommendations that could address identified barriers and facilitate site-level behavioral change.

**Findings**

Clinics ranged in size from 6,000 to 70,000 patients (5% to 37% depression screen positive rates) with 0.5 to 6.0 care manager full time equivalents. Themes related to major implementation barriers concerned *personnel-resources* (e.g., competing care manager demands, inadequate staff) (77% of respondents), *patient engagement* (e.g., no shows/non-adherence) (63%), *team engagement* (e.g., physicians/residents) (50%), and *external factors* (e.g., competing state/national initiatives, psychosocial resources) (40%). Major facilitators involved *patient engagement* (e.g, warm handoffs, personalization) (83%), *team engagement* (e.g., culture change, training, accountability) (80%), and *personnel/resources* (e.g., hiring paraprofessionals for registry/billing) (73%). Themes were predominantly classified under opportunity and motivation (COM-B). Corresponding policy categories for future opportunities included optimizing billing infrastructure and instituting value-based purchasing (*fiscal supports, legislation*), incorporating paraprofessionals and information technology platforms into the CC framework (*environmental/social planning, service provision)*, and providing educational and decision support tools to improve staff, physician and patient engagement (*marketing/communication, guidelines).*

**Implications for D&I Research**

This is one of the first studies to identify barriers and facilitators for implementing a large, state-wide initiative for depression CC in academic centers and to use a theoretical framework to provide concrete suggestions for its future dissemination and successful implementation. Our study suggests that engagement strategies with optimization of registry and billing infrastructures are key to success.

**Primary Funding Source**

National Institutes of Health - NHLBI 3 R01 HL114924-03S1

### S4 Improving transitions from detox to continuing care: using a care transition implementation system

#### Todd Molfenter

##### Center for Health Enhancement System Studies, University of Wisconsin, Madison, WI, 53706, USA

**Background**

With successful transitions from detoxification to continuing care, patients are more likely to remain drug-free or sober, making this is a key transition in the addiction treatment continuum. Yet, just 11-27% of individuals receiving emergency detoxification services for alcohol or drug abuse receive additional treatment or continuing care. This is occurring at a time when drug overdose deaths, primarily due to opioids, are the leading cause of accidental death in the United States.

**Methods**

The system to be tested to improve transitions from detoxification to continuing care integrated three evidence-based implementation science approaches: a) a *practice bundle* of replicable evidence-based practices found to increase detoxification to continuing care transition rates, b*)* a *checklist* to review use of practice bundle elements, and c) an established *standardized organizational change model,* called the NIATx model, to facilitate use of the practice bundle and checklist. The mixed methods analysis compared baseline to post-intervention detoxification to continuing care transition, the efficacy of different practice bundle combinations, and observed the use of the checklist.

**Findings**

The integrated implementation approach improved detoxification to continuing care transition rates from 20% (baseline average) to 43% (post-intervention). The bundle combination that demonstrated the highest transition rates was: a) transitioning patients from detoxification to continuing care in <3 days; b) conducting warm/orchestrated hand-offs (or transfers) between levels of care when possible; and c) collecting data in the detoxification unit to monitor continuing care performance. The process checklists were modified from being used per patient to being used to guide systems change.

**Implications for D&I Research**

The intervention uses an innovative approach based on practice bundles and process checklists, which have been successfully applied in acute care hospital settings, with varied approaches to implementation. The system offers a standardized implementation approach and examines how using bundles and checklists is generalizable to a community-based behavioral health setting. Should the system prove to be generalizable and beneficial, it could provide a tool to address other long-standing public health challenges that are worsened by poor transitions between levels of care.

### S5 A mixed-methods study of system-level sustainability of an evidence-based practice following 12 large-scale implementation initiatives

#### Ashley Scudder^1^, Sarah Taber-Thomas^1,2^, Kristen Schaffner^1^, Amy Herschell^1,3^

##### ^1^Department of Psychiatry, University of Pittsburgh, Pittsburgh, PA, 15213, USA; ^2^Psychology, University at Buffalo, Buffalo, NY, 14260, USA; ^3^Department of Psychology, West Virginia University, Morgantown, WV, 26506, USA

###### **Correspondence:** Amy Herschell

**Background**

As evidence-based practices (EBPs) become more widespread in community behavioral health systems, determining how to systematically sustain (facilitate and optimize) EBPs in complex, multi-level service systems has broad implications for public health. The current study sought to understand the process of sustaining a particular EBP, Parent-Child Interaction Therapy (PCIT), based on the knowledge and experiences of individuals working within existing service systems across states that have employed large-scale implementation initiatives.

**Methods**

This mixed methods study examined rates of sustainment following 12 large-scale initiatives to implement PCIT, factors impacting sustainability, and strategies used to enhance sustainment. Participants were recruited from large-scale PCIT training initiatives using a snow-ball sampling approach. A mixed-methods approach to data collection was utilized; qualitative and quantitative data were sequentially collected and analyzed, using each type of data to answer specific questions and to provide depth and breadth of understanding related to sustainability outcomes and processes.

**Findings**

Sustainment strategies fell into nine categories, including infrastructure, training, marketing, integration, and building partnerships. Strategies involving integration of PCIT into existing practices and quality monitoring predicted sustainment, while financing also emerged as a key factor. The relations among demographic variables, barriers, and strategies were examined, with regression results indicating two strategies (integration into existing practices and monitoring quality) and one barrier (lack of financial funding) as significant predictors of sustainment outcomes. In conjunction with the quantitative data, a grounded theory approach was used to qualitatively identify system-level factors, implementation and initiative factors, and intervention factors related to the facilitation and impediment of the sustainability of PCIT over time.

**Implications for D&I Research**

Authors will discuss implications for the system-level sustainability of EBPs, such as the dynamic interaction among challenges and specific strategies utilized, as well as the reported need of innovation in systems and practice.

**Primary Funding Source**

National Institutes of Health - This research was supported by funding from the National Institute of Mental Health (R01 MH095750)

### S6 Implementing integrated primary care in late adopter sites: the impact of key events on repeated measures of uptake

#### Eva Woodward^1^, Jeffery Pitcock^2^, Mona Ritchie^2^, JoAnn Kirchner^2,3^

##### ^1^MIRECC, VA, North Little Rock, AR, 72114, USA; ^2^QUERI for Team-Based Behavioral Health, Central Arkansas Veterans Healthcare System, North Little Rock, AR, 72114, USA; ^3^Department of Psychiatry and Behavioral Sciences, University of Arkansas for Medical Sciences, Little Rock, AR, 72205, USA

###### **Correspondence:** Eva Woodward

**Background**

Integrating behavioral health care into primary care is a complex behavioral health innovation and often warrants facilitation—an umbrella implementation strategy—to increase its adoption, especially in late adopter sites. We conducted a secondary data analysis from a two-year hybrid Type II study using facilitation to promote implementation of integrated primary care across seven VA medical clinics. Our aim was to (a) identify patterns of adoption across clinics and (b) identify associations between key events that occurred during implementation and adoption.

**Methods**

For each clinic, we synthesized quantitative and qualitative data. First, we utilized a quantitative B-spline function to map out a pattern of continuous, linear combination of multiple repeated (monthly) measures of adoption—this accounted for correlations between time points. Second, we used qualitative data from program records, debriefing interviews with facilitators, and facilitator time logs to identify key events that occurred during implementation facilitation (e.g., staff turnover). Two coders categorized key events into domains of the theoretical framework that informed the study—Integrating Promoting Action on Research for Health Services (i-PARIHS). Then, we created a visual overlay of key events on to mapped patterns of adoption.

**Findings**

Adoption increased over time, although with great variability—it was more rapid for smaller clinics. Events that inhibited adoption over time included staff loss and hiring of staff and leadership not trained in the innovation. Events that promoted adoption over time included facilitator site visits, positive changes in leadership, and creating and reviewing implementation-planning checklists.

**Implications for D&I Research**

When using facilitation for complex innovations in healthcare, preliminary results suggest that site visits, continuous assessment of implementation-planning checklists, and staff loss or mismatch to the innovation are most impactful on adoption. Though it presents methodological challenges, an advantage of using repeated measures is that, rather than having only a snapshot of adoption at a few, discrete time points, we were able to assess variability in adoption by using a nearly continuous measure of repeated assessments over two years. One challenge is validity of associations between adoption and key events, due to the multifactorial nature or “shocks” of the data over time.

**Primary Funding Source**

Department of Veterans Affairs - Health Services Research and Development and QUERI grants

### S7 Enhancing organizational capacity in implementation science: an evaluation of the practicing knowledge translation course

#### Julia E. Moore^1^, Sobia Khan^1^, Shusmita Rashid^1^, Jamie Park^1^, Melissa Courvoisier^1^, Sharon Straus^1,2^

##### ^1^ Knowledge Translation (KT) Program, Li Ka Shing Knowledge Institute, St. Michael's Hospital, Toronto, ON, M5B 1 T8, Canada; ^2^ Geriatric Medicine, University of Toronto, Toronto, ON, M5G 1 V7, Canada

###### **Correspondence:** Julia E. Moore

**Background**

Effective evidence implementation can help health care organizations optimize quality of care. A challenge is lack of organisational capacity in implementation. Building on a previous evaluation, we developed a 6-month course, Practicing Knowledge Translation (PKT), designed to teach best practices in high-quality implementation and equip participants with the core competences required to integrate evidence-informed practices. We conducted an outcome evaluation of the course.

**Methods**

The 15 PKT course participants included professionals working in healthcare, government, and non-government organizations that support healthcare delivery or funding. The primary outcome was an increase in *knowledge* and *self-efficacy* across six core competencies (developing an evidence-informed, theory-driven program (ETP); implementation; evaluation; sustainability, spread, scale up; contextual assessment; stakeholder engagement) measured through surveys (likert 1-7) completed before (time 1), immediately after (time 2), and 7 months after (time 3) the course. Secondary outcomes included *use of implementation* (likert 1-5) and satisfaction with the course based on semi-structured interviews. Descriptive statistics and repeated-measures ANOVA and Bonferroni post-hoc models were used for quantitative data analysis to evaluate changes in knowledge and self-efficacy in SPSS v.20. Data from qualitative interviews will be analyzed in NVivo.

**Findings**

Survey response rate was 60-80% across all time points (Time 1, n = 12; Time 2, n = 12; Time 3, n = 9). Participants’ *knowledge* and *self-efficacy* increased in four and five of the six core competencies, respectively: developing an ETP (*p* = .06; *p* = .01), implementation (*p* = .002; *p* = .001), evaluation (*p* = .003; *p* = .03), sustainability/scale/spread (*p* = .002; *p* = .02), contextual assessment (*p* = .012; *p* = .002), and stakeholder engagement (*p* = .06; *p* = .13). There was no change in current implementation practice after the course was delivered (*p* = .12). Qualitative data are being analyzed and will be available for presentation.

**Implications for D&I Research**

Quantitative findings indicate that the PKT course increased participant knowledge and self-efficacy across 5 of 6 core competencies. There is a need to evaluate the impact of implementation training on participants’ knowledge of and self-efficacy in using implementation science in their projects and organizations. Findings from this evaluation can be used to inform future training initiatives.

### S8 Implementation barriers and facilitators of treatments for criminogenic thinking in the Veterans Health Administration

#### Daniel Blonigen^1,2^, Allison Rodriguez^3^, Luisa Manfredi^1^, Andrea Nevedal^1,4^, Joel Rosenthal^5^, David Smelson^6,7^, Christine Timko^1^

##### ^1^HSR&D Center for Innovation to Implementation, Department of Veterans Affairs Palo Alto Health Care System, Menlo Park, CA, 94025, USA; ^2^Clinical Psychology PhD Program, Palo Alto University, Palo Alto, CA, 94304, USA; ^3^VA Palo Alto Health Care System, National Center for PTSD, Menlo Park, CA, 94025, USA; ^4^Health Services Research and Development, Veterans Health Administration, Menlo Park, CA, 94025, USA; ^5^Office of Homelessness/Veterans Justice Programs, Department of Veterans Affairs, Menlo Park, CA, 94025, USA; ^6^Center for Healthcare Organization and Implementation Research (CHOIR), VA Health Services Research & Development, Bedford/Boston, MA, 01730, USA; ^7^Department of Psychiatry, University of Massachusetts Medical School, Worcester, MA, 01655, USA

###### **Correspondence:** Daniel Blonigen

**Background**

With the rise of specialty courts, behavioral health services are increasingly called upon to treat criminal offenders in order to reduce offenders’ risk for recidivism. *Criminogenic thinking* (antisocial cognitions/attitudes) is the risk factor with the strongest association with criminal recidivism. Cognitive-behavioral treatments for criminogenic thinking–e.g., Moral Reconation Therapy (MRT); Thinking 4 a Change (T4C)–were developed within correctional settings and are regarded as best practices for reducing recidivism. However, knowledge of their implementation potential within behavioral health services is unknown. To address this gap, we identified barriers to implementation of cognitive-behavioral treatments for criminogenic thinking in the Veterans Health Administration (VHA), and facilitators that could serve as solutions to these barriers.

**Methods**

Specialists from the VHA’s Veterans Justice Programs–a nationwide outreach and linkage service for justice-involved veterans–were recruited to participate in a semi-structured phone interview to describe their practices regarding treatment of criminal recidivism (*n* = 63). Participants who had been trained in MRT or T4C (n = 22) were asked questions regarding the implementation potential of these interventions in VHA. Thematic coding and pile-sorting techniques were used to identify barrier and facilitator themes at the patient-, program-, and system levels.

**Findings**

The time-intensiveness of these treatments emerged as a barrier to implementation. Potential solutions identified were patient incentives for treatment engagement, streamlining the curriculum, and implementing the treatments within long-term/residential programs. At the program level, providers’ stigma/bias towards patients with antisocial tendencies was seen as a barrier to implementation, as were time/resource constraints on providers. To address the latter, use of peer providers to deliver the treatments and partnerships between justice programs and behavioral health services were suggested. At the system level, lack of recognition of criminogenic treatments as evidence based, and uncertainty of sustained funds to support ongoing costs of these treatments emerged as implementation barriers. To address the latter, a “train-the-trainers” model was suggested.

**Implications for D&I Research**

Our findings serve as a guide for implementation of criminogenic treatments for providers/policymakers in VHA and other large healthcare systems that are increasingly called upon to provide care to justice-involved adults–a large and highly vulnerable subgroup of the behavioral health population.

**Primary Funding Source**

Department of Veterans Affairs - VA Health Services Research & Development (RRP 12-507)

### S9 Therapist and leader attitudes towards evidence-based practices: influence of practice, staff role, and perceptions of organizational climate

#### Nicole Stadnick^1^, Jennifer Regan^2^, Miya Barnett^3^, Anna Lau^4^, Lauren Brookman-Frazee^1^

##### ^1^Psychiatry, University of California, San Diego, La Jolla, CA, 92093, USA; ^2^Clinical Training and Evidence-Based Practice, Hathaway-Sycamores Child and Family Services, Pasadena, CA, 91105, USA; ^3^Counseling, Clinical, & School Psychology, University of California, Santa Barbara, Santa Barbara, CA, 93106-9490, USA; ^4^Psychology, University of California, Los Angeles, Los Angeles, CA, 90095, USA

###### **Correspondence:** Nicole Stadnick

**Background**

Organizational factors, such as implementation climate and workforce burnout, play an influential role on attitudes towards implementation of evidence-based practices (EBPs) in mental health systems. Both line staff and administrators are integral to EBP implementation efforts, and it is important to understand whether attitudes toward specific EBPs vary between agency leaders and therapists. Further, it is plausible that distinctive organizational factors differentially impact leader versus therapist attitudes toward EBPs.

**Methods**

This study examined leader and therapist attitudes towards EBPs within the context of a system-level reform mandating the implementation of multiple EBPs through reimbursement policies in children’s mental health services in Los Angeles County. Study objectives were to: 1) determine whether leaders and therapists differed in their attitudes toward six EBPs, and 2) identify organizational factors associated with leaders’ and therapists’ practice-specific attitudes. Data were obtained from 181 leaders and 763 therapists who completed an online survey that assessed perceptions of workplace burnout and organizational functioning using subscales from the Organizational Climate Measure and Organizational Readiness for Change. Hierarchical linear models were specified with practice (L1) nested within leader/therapist (L2) nested within agency (L3) to predict practice-specific attitudes (e.g., ease of use, adaptability to fit to service setting).

**Findings**

There were significant cross-level interactions between practice and informant, indicating that leaders and therapists reported differing preferences for EBPs. Furthermore, distinctive organizational factors interacted with practice to predict leader and therapist attitudes. For example, leader perceptions of staff cohesion were associated with more positive appraisals for most practices. Therapist perceptions of burnout were associated with more negative evaluation of some practices but more positive evaluation of others.

**Implications for D&I Research**

Understanding the unique perspectives of different types of staff towards EBPs can inform EBP implementation efforts. Findings indicate that leaders and therapists differ in their preferences for practices and these differences may be shaped by their perceptions of organizational functioning. Results highlight the importance of considering organizational characteristics such as staff cohesion and burnout when selecting and implementing EBPs to promote uptake and sustainment in service systems.

**Primary Funding Source**

National Institutes of Health - Funding for this research project was supported by the following grants from NIMH: R01 MH100134 and R01 MH100134-S.

### S10 The influence of leadership at executive and middle management levels on the implementation of evidence-based behavioral health care practices

#### Erick Guerrero^1^, Karissa Fenwick^2^, Yinfei Kong^3^, Gregory Aarons^4^

##### ^1^Communities, Organizations and Business Innovation, School of Social Work, and Marshall School of Business, University of Southern California, Los Angeles, CA, 90015, USA; ^2^School of Social Work, University of Southern California, Los Angeles, CA, 90015, USA; ^3^Mihaylo College of Business and Economics, California State University, Fullerton, CA, 90089, USA; ^4^Psychiatry, UC San Diego, La Jolla, CA, 92083-0812, USA

###### **Correspondence:** Erick Guerrero

**Background**

Leadership plays a critical role in the implementation of evidence-based practices (EBPs) in behavioral health settings. While most research focuses on a single leader or a broad leadership style (e.g. transformational approach), this study examined the impact of both broad and focused leadership and multiple leadership levels on implementation of EBPs. Guided by social exchange theory, we proposed that mid-level, implementation-focused leadership would enhance how executive level transformational leadership affects 1) staff attitudes toward EBPs, and 2) implementation of contingency management treatment (CMT) and medication-assisted treatment (MAT).

**Methods**

We collected multilevel data in 2013 from 427 employees embedded in 112 behavioral health treatment programs in the largest addiction health services system in the United States. We measured transformational leadership using the Survey of Transformational Leadership, implementation-focused leadership using the Implementation Leadership Scale, and attitudes towards EBPs using the Evidence-Based Practice Attitudes Scale. We assessed implementation of CMT and MAT using a 5-point Likert Scale indicating how often the EBP is used in the program. We tested a multilevel path analysis with bootstrap standard errors using Stata, 13.

**Findings**

Results showed a positive relationship between transformational executive leadership and mid-level implementation-focused leadership (standardized direct effect = .173, bootstrap *p* < .03). As expected, transformational leadership was indirectly associated with employee attitudes toward EBPs via mid-level implementation leadership (standardized indirect effect = .090, bootstrap *p* < .01). Also, transformational executive leadership was indirectly associated with delivery of CMT through mid-level implementation-focused leadership (standardized indirect effect = .006, bootstrap *p* < .09), though this relationship was partially significant. The relationships between leadership types and MAT were not statistically significant.

**Implications for D&I Research**

This study offers theoretical contributions to social exchange and leadership theories as applied to implementation of EBPs in behavioral health settings. Findings highlight the importance of testing leadership process using mediators in order to unpack how leadership may cascade to different levels. Results highlight the importance of training mid-level management on implementation leadership to enhance the uptake of CMT in addiction health services.

**Primary Funding Source**

National Institutes of Health - R33DA035634-03, PI: Erick Guerrero, R25 MH080916, PI: Enola Proctor.

### S11 A multilevel case study of act implementation

#### Rebecca Lengnick-Hall, Karissa Fenwick, Benjamin Henwood

##### School of Social Work, University of Southern California, Los Angeles, CA, 90015, USA

###### **Correspondence:** Rebecca Lengnick-Hall

**Background**

Commonly used implementation frameworks—such as the Consolidated Framework for Implementation Research (CFIR) and the Exploration, Preparation, Implementation, and Sustainment (EPIS) framework—describe the multilevel nature of evidence-based practice (EBP) implementation. These frameworks highlight the ways in which inner and outer contextual features overlap, interact, and influence implementation success. This study uses a multilevel case study method to understand the implementation of Assertive Community Treatment (ACT) in Los Angeles County.

**Methods**

Focusing on fidelity as a key implementation outcome, we conducted semi-structured interviews to explore ACT implementation experiences at three levels: county, agency leadership, and frontline staff. The sample included county level experts as well as 35 providers and managers at three organizations currently implementing ACT in L.A. County. Interview questions assessed knowledge of the ACT model, history of ACT implementation, fidelity, and the use of ACT in everyday clinical practice. Experts and managers addressed outer contextual features such as legislative decisions, funding structures, and contracting arrangements. Frontline staff and managers addressed inner contextual features such as organizational characteristics, employee attitudes, and EBP fit.

**Findings**

Findings revealed important relationships and differences among the three levels of ACT implementation, in particular relating to definition of the ACT model and conceptualization of fidelity. At the provider level, frontline staff offered many examples of 'doing' ACT, yet reported little explicit knowledge of model components or fidelity measurement. This was consistent with county administrator interviews describing the California programs as 'ACT like' and designed to be flexible, but also contrasted with administrator and director statements about the importance of achieving fidelity to core components.

**Implications for D&I Research**

This study demonstrates how a multilevel case study approach can capture and describe the nuances and complexity of EBP implementation. First, this design enabled us to link specific outer contextual features to behaviors and perspectives among those who are using ACT on a day-to-day basis. Second, this design allowed us to explore and assess differences across the three levels of analysis. Findings offer concrete examples of the relationships described in implementation frameworks.

### S12 Organizational factors differentiating VA PTSD outpatient teams with high and low delivery of evidence-based psychotherapy

#### Nina Sayer^1,2^, Craig Rosen^3,4^, Robert Orazem^1^, Brandy Smith^3,5^

##### ^1^Center for Chronic Disease Outcomes Research, Minneapolis VA Health Care System, Minneapolis, MN, 55417, USA; ^2^Medicine and Psychiatry, University of Minnesota, Minneapolis, MN, 55454, USA; ^3^VA Palo Alto Health Care System, National Center for PTSD Dissemination & Training Division, Menlo Park, CA, 94025, USA; ^4^Department of Psychiatry and Behavioral Sciences, Stanford University School of Medicine, Stanford, CA, 94304, USA; ^5^VA Palo Alto Health Care System, Center for Innovation to Implementation, Menlo Park, CA, 94403, USA

###### **Correspondence:** Nina Sayer - Medicine and Psychiatry, University of Minnesota, Minneapolis, MN, 55454, USA

**Background**

As part of a broad effort to implement evidence-based care, the Veterans Health Administration (VHA) has been working over the past decade to expand use of Cognitive Processing Therapy (CPT) and Prolonged Exposure (PE) for treatment on posttraumatic stress disorder (PTSD). VHA's primary strategy has been national training initiatives efforts that target individual clinicians. Less attention has been given to team-level processes, and identifying local organizational factors that promote higher levels of use of CPT and PE.

**Methods**

This mixed methods study used automated processing of chart notes to select PTSD clinics with high and low use of CPT and PE. Rapid Assessment Process methodology was then used to compare teams along dimensions known to affect implementation and sustainability of a practice improvement program. The primary method of data collection was semi-structured interview with 100 staff from nine VA medical centers which had PTSD teams that differed in reach of CPT and PE to therapy patients with PTSD. Qualitative analysis involved constant comparison of high, medium and lower reach teams in terms of the identified themes.

**Findings**

High reach teams embraced a mission to deliver evidence-based psychotherapies for PTSD. This mission was reflected in staff beliefs about CPT and PE, enacted through clinic operations and supported by resources and leadership outside the teams. Lower reach teams were less specialized and situated in practice environments with less support and movement of patients between mental health teams. Medium reach teams shared characteristics of both high and lower reach teams.

**Implications for D&I Research**

Widespread implementation of PE and CPT requires not only training clinicians to deliver these treatments, but also addressing team and facility-level organizational factors. High reach teams prioritized evidence-based treatments and either de-implemented or shifted to other clinics delivery of other types of care. Teams that added EBPs without de-implementing other clinical responsibilities had lower reach.

**Primary Funding Source**

Department of Veterans Affairs

### S13 Engaging clinicians and veterans in efforts to decrease benzodiazepines in posttraumatic stress disorder (PTSD): de-implementing through academic detailing

#### Craig Rosen

##### VA Palo Alto Health Care System, National Center for PTSD, Dissemination and Training Division, Menlo Park, CA, 94025, USA; Psychiatry and Behavioral Sciences, Stanford University School of Medicine, Menlo Park, CA, 94025, USA; Veterans Affairs Palo Alto Healthcare System, Center for Innovation to Implementation, Menlo Park, CA, 94025, USA

###### **Correspondence:** Craig Rosen

**Background**

Benzodiazepines are not recommended by practice guidelines for the treatment of PTSD. Despite these recommendations, providers routinely prescribe benzodiazepines in patients diagnosed with PTSD. Our research group has identified subgroups of Veterans with PTSD in whom the rate of prescribing of benzodiazepines is higher and to whom benzodiazepine use poses increased risk of harm. Resulting non-adherence to the guideline recommendations suggests that increased education, training, and consultation to both clinicians and Veterans can help ‘de-implement’ the use and harm of these agents.

**Methods**

Our team conducted research locally and in Northern California to examine the use of an Academic Detailing (AD) intervention to share developed decision support tools (brochures for both providers and patients). A clinical pharmacist met with providers in individualized outreach visits at their practice site to promote evidence-based treatment options. A dashboard developed by VA’s Pharmacy Benefits Management identified providers and patient-level prescribing data and allowed a focus on high-risk patients on a provider’s panel. Brochures were shared, key clinical messages about benzodiazepines in PTSD were discussed, and safer treatment alternatives were reviewed. Direct-to-consumer strategies were used by mailing patient brochures to identified subgroups of Veterans and were asked to discuss the content with their provider at upcoming visits.

**Findings**

Not all clinical pharmacists are strong detailers. We experienced variability in the skill of our pharmacists to engage providers in discussions of decreased benzodiazepine use. We also learned that clinical providers are typically too busy to have time to use performance dashboards designed to improve care. So the detailer has to offer brief key messages, clinical shortcuts, and resources to engage prescribing clinicians in a dialogue. Decreases in incidence and prevalence rates of benzodiazepine prescribing have been variable. More success has been observed locally due to repeated visits by the detailer.

**Implications for D&I Research**

AD is a promising and potentially efficacious de-implementation strategy, but it requires interpersonal skill and repeated contacts for it to be effective.

**Primary Funding Source**

Department of Veterans Affairs

### S14 Participatory system dynamics: triangulating electronic health records, stakeholder expertise and simulation modeling to expand evidence-based practices

#### Lindsey Zimmerman^1,2^, David Lounsbury^3,4^, Craig Rosen^1,5,6^, Rachel Kimerling^1^, Jodie A. Trafton^5,6,7^, Steven Lindley^5,8^

##### ^1^VA Palo Alto Health Care System, National Center for PTSD, Dissemination and Training Division, Menlo Park, CA, 94025, USA; ^2^Psychiatry and Behavioral Sciences, University of Washington School of Medicine, Seattle, WA, 98195, USA; ^3^Epidemiology and Population Health, Albert Einstein College of Medicine, Bronx, NY, 10467, USA; ^4^Division of Community Collaboration and Implementation Science, Albert Einstein College of Medicine of Yeshiva University, Bronx, NY, 10467, USA; ^5^Psychiatry and Behavioral Sciences, Stanford University School of Medicine, Menlo Park, CA, 94025, USA; ^6^Veterans Affairs Palo Alto Healthcare System, Center for Innovation to Implementation, Menlo Park, CA, 94025, USA; ^7^VA Program Evaluation and Resource Center, VA Office of Mental Health Operations, Menlo Park, CA, 94025, USA; ^8^Outpatient Mental Health, Veterans Affairs Palo Alto Healthcare System, Menlo Park, CA, 94025, USA

###### **Correspondence:** Lindsey Zimmerman

**Background**

In some settings, evidence-based practices (EBPs) may be adopted by providers, prioritized by leadership and supported by health system infrastructure, yet still not reach an adequate proportion of patients. In these systems, local staff expertise and operations data can be synthesized in a participatory system dynamics (PSD) model, developed for selecting strategies to improve EBP reach. PSD modeling simulation empowers stakeholders by enabling them see the potential yield of implementation plans prior to implementation.

**Methods**

Our team worked with Veteran patients, local leadership, psychiatrists, psychologists, social workers, nurses and clerks to develop a PSD model of Veterans Health Administration (VA) outpatient mental health care. Our team coordinated with national VA offices responsible for disseminating evidence-based psychotherapy and pharmacotherapy, and conducting quality assurance. Qualitative group modeling sessions with frontline staff included developing the model structure, reviewing data inputs for validity, and model calibration checks against the historical behavior of the clinical system. Stakeholders provided a list of restructuring scenarios intended to improve EBP reach, which were evaluated with simulation.

**Findings**

Using a model parameterized with local clinic data, stakeholders viewed the operational mechanisms in their settings that they believed could expand EBP reach. Interdependent relationships among model parameters, such as patient factors (e.g., missed appointment rates), and system capacities (e.g., staffing) were examined. Stakeholders determined which implementation plan was likely to increase EBP reach the most, drilling down to examine the entire EBP continuum: referral to EBP, scheduling EBPs, initiating an EBP, completing a therapeutic dose of an EBP and patient benefit from an EBP.

**Implications for D&I Research**

PSD models evaluate stakeholders’ theories of clinic operation, testing explanatory mechanisms (i.e., local policies and procedures) by which EBP reach could be improved. PSD capitalizes on health record data, stakeholder expertise and simulation to optimize implementation. PSD may be a strategy for more timely patient access to EBPs even when no new system resources (e.g., new staff, new EBP trainings) are available. The PSD process increases staff general capacity for quality improvement, while the PSD modeling tool identifies implementation strategies tailored to local EBP-specific capacities and constraints.

**Primary Funding Source**

Department of Veterans Affairs

## Big Data and Technology for Dissemination & Implementation Research

### S15 Media influence and framing the public health narrative around unarmed deaths, race, and gun violence: mapping the Trayvon Martin case

#### Rahul Bhargava^1,2^

##### ^1^Media Lab, Massachusetts Institute of Technology, Cambridge, MA, 02139, USA; ^2^Berkman Klein Center for Internet & Society, Harvard Law School, Cambridge, MA, 02138, USA

###### **Correspondence:** Rahul Bhargava

**Background**

Restrictions on gun-related research in the US create barriers to fully understanding the health implications of gun violence on racial disparities. Media coverage of gun related deaths and violence, particularly incidents that result in the death of unarmed African Americans, helps illustrate the gun violence landscape. The killing of unarmed teen Trayvon Martin offers a potent case study for examining the potential influence of participatory media on framing gun violence and race as a public health issue.

**Methods**

We collected data on the Trayvon Martin case from Media Cloud sources to analyze the media coverage volume and network. Conceiving of the media ecosystem as a network demanded a network analysis approach, for which we used Gephi and the PageRank algorithm. We complemented and informed the direction of our quantitative analysis through qualitative interviews with media activists involved in the early stages of the Trayvon Martin story. Peak coverage and the linked network of publishers were mapped from the date of his death, February 26, 2012, to April 30, 2012.

**Findings**

News coverage started as a short-lived, local Florida news piece. Media pressure grew after Martin’s family hired a civil rights attorney, and a few large influential media outlets generated the racial framing of the story. 8643 total sources linked to one another and the civil rights narrative was carried across the national media network through hyperlinks. Social media clicks (n = 1.2million) provided a proxy for readership and resulting action offline. Broadcast media remained an amplifier and gatekeeper of the narrative, but participatory public media activism co-created the framing of the story’s ultimate message about race

**Implications for D&I Research**

These findings show the utility of viewing online media influence metrics in the context of a network of diverse actors. Our results suggest that this network influenced the public discourse, sentiment, and response to gun violence and race. Mapping the network of online media sources that create, spread, and reinforce messages illustrates how the general public shapes the conversation through their own narratives. Network mapping provides opportunities for identifying professional and public influencers who can assist in framing gun violence and racial disparities as public health issues

**Primary Funding Source**

John D. and Catherine T. MacArthur Foundation, Open Society Foundation, and Ford Foundation

### S16 Vaccine hesitancy and digital networks: the influence of social proof

#### Hal Roberts

##### Berkman Klein Center for Internet and Society, Harvard University, Cambridge, MA, 02138, USA

**Background**

Health experts recognize childhood vaccinations as one of the most important public health achievements of the twentieth century. The Internet, directly linked to vaccine hesitancy, is a well-documented health information source. The rise in numbers and combinations of vaccines along with globalized, non-hierarchical communication make today’s challenge of overcoming vaccine hesitancy particularly complex. Despite a variety of efforts to address this challenge, online vaccine hesitant communities remain robust.

**Methods**

We used Media Cloud’s searchable archive of over 350 million stories from 50 thousand media sources, along with tools to analyze that archive, to conduct quantitative and qualitative network analysis based on key word selection and community detection. We generated a network map of sources who published about vaccines from June 1, 2014 to March 1, 2015. We measured influence via hyperlink degree centrality, betweenness centrality, degree of freedom of speech, and sentiment for the top 400 sources. Pearson chi squared tests determined magnitudes of associations.

**Findings**

4,187 identified sources formed distinct networked communities based on hyperlink sharing. Those link-sharing communities separated cleanly into pro and vaccine hesitant groups. Pro vaccine sources were directly linked to most frequently (p = 0.027), but highly connected sources within the network were twice as likely to portray vaccine hesitant sentiment contrary to evidence (p = 0.03). Sources that were more successful in spreading their information through linked connections were non-evidence based (p = 0.001) with low or no degree of scientific peer review. (p = 0.033).

**Implications for D&I Research**

The social structure and function of the Internet means network science and visualization mapping is essential in understanding relationships and connections between various publishers. These findings demonstrate an online environment where scientific evidence may not be as influential as social proof, a form of imitation where individuals ascribe to social behaviors of others to resolve uncertainty, such as link sharing. A challenge for childhood vaccinations communication may not only be poor quality information online but also the norms and values of the most influential online social networks sharing that information. Understanding online influence and network sociology is key to maximizing the effectiveness of digital and social communications necessary for sustaining optimal vaccine acceptance and reducing vaccine hesitancy.

### S17 Could fragmented communication networks reshape the narrative?: Evidence from tobacco and e-cigarette media framing

#### Laura Gibson

##### Tobacco Center of Regulatory Science, University of Pennsylvania School for Communication, Philadelphia, PA, 19104, USA

**Background**

Youth smoking behavior in 2003 could be predicted from content analysis of the public communication environment around tobacco. The more frequently tobacco appeared in the news (e.g., describing negative health effects and anti-smoking policies), the less youth used tobacco. However since 2003, media platforms have diversified – as have tobacco products.

**Methods**

Text from websites popular among 12-24 year olds were pulled from the Media Cloud database via tobacco terms. Three traditional media sources. (Associate Press [AP], top 50 U.S. newspapers, and broadcast news transcripts) were pulled by searching the Lexis-Nexis database for the same set of tobacco terms. Supervised learning was used to classify this initial sample of potentially related texts (n = 79,020) as truly tobacco-related (n = 62,232). We calculated the three-day frequency of mentions of each topic for each source (n =136). Online and traditional media were compared.

**Findings**

E-cigarette mentions accounted for (n = 4,167, 7%) while tobacco only mentions were the majority (n = 58,065, 93%). Results for both topics showed moderate coordination between websites with broadcast news and AP. Coordination was significant for e-cigarette coverage across all sources (r = .44), suggesting that different platforms are picking up on the same e-cigarette narrative of events. For tobacco, message coordination across websites and newspapers was absent.

**Implications for D&I Research**

Comparing new online networked messaging with traditional media sources allows us to evaluate how the narrative is changing as media evolve and new tobacco products emerge over time. Implications of this approach include visualizing general trends in narratives across the publishing network (rather than single issues or events), mapping of all data available from every source, and the inclusion of many examples of sources for each platform (i.e., multiple newspapers and websites). However e-cigarettes in particular are a controversial product, so there could still be fragmentation in type of coverage. Follow-up analysis of specific content of e-cigarette news coverage across sources will provide further information about whether or not the public communication environment is fragmented on this topic. Network science and visualization models can assist in improved hyperlinking and generating new connections for dissemination of optimally coordinated messaging around tobacco and e-cigarettes.

**Primary Funding Source**

National Institutes of Health - National Cancer Institute (NCI) of the National Institutes of Health (NIH) and FDA Center for Tobacco Products (CTP) under Award Number P50CA179546.

### S18 Implementation of real time clinical decision support: the challenge of evaluation

#### Gabriel J Escobar^1^, Vincent Liu^2^, Benjamin Turk^2^, Arona Ragins^2^, Patricia Kipnis^3^

##### ^1^Division of Research, Kaiser Permanente, Oakland, CA, 94612, USA; ^2^Health Care Effectiveness, Kaiser Permanente Division of Research, Oakland, CA, 94612, USA; ^3^Decision Support, Kaiser Permanente Northern California, Oakland, CA, 94612, USA

###### **Correspondence:** Gabriel J Escobar

**Background**

Comprehensive electronic medical records (EMRs) permit access to large datasets with granular clinical data and can serve as platforms for clinical decision support (CDS). In theory, this combination should facilitate testing, implementation and evaluation of CDS tools to improve patient outcomes. In practice, demonstration of clinical benefit remains difficult. We describe challenges encountered in evaluation of two inpatient CDS systems deployed by Kaiser Permanente Northern California.

**Methods**

Using data from >1.0 M patients, we developed an early warning system for patient deterioration outside the intensive care unit (ICU) and a risk score for non-elective rehospitalization. The early warning system, deployed at 2 hospitals in 2013-2014, provides discrete, patient-specific probability estimates every 6 hours. The rehospitalization risk score, deployed in 7 hospitals in 2016, generates patient-specific probability estimates every morning. Implementation teams developed clinician workflows for both systems. Given KPNC’s 22 hospitals and unitary information capture (single EMR across the entire enterprise), we can assign probability estimates to patients at the pilot sites and virtual probability estimates at the other sites. This permits evaluating the CDS by comparing patient outcomes from pilot and non-pilot hospitals (adjusting for risk differences via multivariate matching, since technical limitations precluded randomization).

**Findings**

Based on observed mortality, rehospitalization, and cost decreases at the pilot sites, KPNC will deploy these two CDS systems in all its hospitals. We encountered these challenges in the evaluation process: selection of outcomes measures - including the problem of employing mortality as an outcome, since patients near the end of life may not desire further intervention; inability to access real time data for model development; lack of concordance between real time data used by CDSs and data that are stored for retrospective analyses; computational challenges involving extremely large datasets; need for balancing measures (e.g., possible increases in admissions to the ICU); and differential effects across study sites

**Implications for D&I Research**

Although the use of EMRs as platforms for real time CDS holds great promise, quantification of benefit is likely to remain challenging in the near future. Greater attention should be given to methodological aspects of the evaluation of CDS impact.

**Primary Funding Source**

Other (please specify below) - Gordon and Betty Moore Foundation

### S19 Reducing readmissions through improving care transitions (RRTICT): development of a tailored approach to improve veteran outcomes

#### Ashley Ketterer Gruszkowski^1^, Michael W. Kennedy^1^, Emily Rentschler Drobek^1^, Lior Turgeman^2^, Aleksandra Sasha Milicevic^2^, Terrence L. Hubert^1^, Larissa Myaskovsky^1,3,4^, Youxu C. Tjader^2^, Robert J. Monte^1^, Kathryn G. Sapnas^5^

##### ^1^Veterans Engineering Resource Center, VA Pittsburgh Healthcare System, Pittsburgh, PA, 15125, USA; ^2^Joseph M. Katz Graduate School of Business, University of Pittsburgh, Pittsburgh, PA, 15260, USA; ^3^Center for Health Equity Research and Promotion, VA Pittsburgh Healthcare System, Pittsburgh, PA, 15240, USA; ^4^University of Pittsburgh School of Medicine, University of Pittsburgh, Pittsburgh, PA, 15240, USA; ^5^Office of the Assistant Deputy Under Secretary of Health for Patient Care Services, VA Central Office, Washington, DC, 20420, USA

###### **Correspondence:** Ashley Ketterer Gruszkowski

**Background**

The Pittsburgh Veterans Engineering Resource Center (VERC) partnered with the University of Pittsburgh and the VA Office of Patient Care Services to develop the Reducing Readmissions through Improving Care Transitions (RRTICT) program. RRTICT is a suite of care strategies that combines 32 best practices from Veterans Administration Medical Centers (VAMC) and health services literature. The use of a near real-time readmission risk predictive model to assist clinical decision making and equitable provisions of care differentiates RRTICT from other programs.

**Methods**

The RRTICT model was derived using a set of over 7000 patients and has an 80% accuracy rate. It uses administrative, clinical, and patient descriptive data from the VA Corporate Data Warehouse to predict risk of hospital readmission (within 30-days of discharge, 30DRR) at initial hospital admission. Six VAMCs volunteered to pilot RRTICT for six months in FY15. Participating sites had to agree to engage a discharge team, participate on monthly calls, and submit a midpoint and final report. The model provided daily risk values for each patient on the pilot wards. At the conclusion of the pilot, the VERC used the VA Inpatient Evaluation Center (IPEC) all-cause readmission rate definition to calculate the 30DRR on the patients in the pilot sites. Calculated rates were compared to rates from the same six-month period in FY 2014 using the two proportions Z-test.

**Findings**

Sites that implemented RRTICT demonstrated an overall decrease in 30DRR by 28% (Table 1).

**Implications for D&I Research**

RRTICT provides a structured framework that helps translate evidence based interventions into clinical practice. By using “Big Data,” the RRTICT predictive model allows clinical teams to maximize their use of best practices to achieve the greatest equity of care. In addition, the success of reducing readmissions is encouraging further interest to expand RRTICT throughout the VHA.

**Primary Funding Source**

Department of Veterans Affairs

**Table 1:** RRTICT 30DRR Results

Site	Readmission Rate Difference	Percent Difference	P Value
Overall	−0.044	−28%	0.00001
Albany	−0.013	−9%	0.59992
Bay Pines	−0.043	−27%	0.00937
Gainesville	−0.056	−22%	0.24760
Omaha	−0.053	−35%	0.05105
San Juan	−0.077	−48%	0.00011
Sioux Falls	−0.021	−17%	0.32614

### S20 Work system redesign and implementation of evidence-based hypertension protocol in specialty clinics using electronic health record prompts

#### Edmond Ramly^1^, Diane R Lauver^2^, Christie M Bartels^3^

##### ^1^Industrial and Systems Engineering, University of Wisconsin-Madison College of Engineering, Madison, WI, 53704, USA; ^2^Nursing, University of Wisconsin School of Nursing, Madison, WI, 53704, USA; ^3^Medicine, Rheumatology Division, University of Wisconsin School of Medicine and Public Health, Madison, WI, 53705, USA

###### **Correspondence:** Edmond Ramly

**Background**

Hypertension protocols improve hypertension control and CVD prevention in primary care but have not been tailored for implementation in specialty clinics, although specialty visits outnumbered US primary care visits in 2013. We aimed to engage clinic staff in work system redesign to tailor and implement a blood pressure (BP) protocol for specialty clinics using electronic health record (EHR) prompts.

**Methods**

Over six months, our multidisciplinary study team (specialty MD, nursing, systems engineering) engaged staff from three specialty clinics in focus groups, specialty-relevant education, EHR prompted workflows, and audit feedback. EHR alerts prompted routine re-measurement and EHR order set assisted with primary care follow up orders after elevated BPs. Four monthly interactive 1-on-1 audit and feedback sessions with staff used a Self Determination Theory-informed process to continuously identify and overcome barriers to improving protocol fidelity and reach. EHR data was used to monitor implementation outcomes: re-measurement of elevated BP, follow up orders, and timely follow up. We also administered a retrospective anonymous 15 item staff questionnaire.

**Findings**

We compared implementation outcomes for all visits with elevated BP at baseline and in the intervention study months. BP re-measurement improved from <2% of visits to 83%. Follow up orders for patients with confirmed elevated BP improved from 0% to 73%. Monthly patient-completed timely follow up improved from 29% to 71%, doubling timely follow-up in multivariable analysis (OR 2.1, CI 1.4-3). All groups benefited and black patients showed additional gains compared to white patients (OR 1.7, CI 1.1-2.5). Gains were sustained at 18 months. Additionally, the percentage of staff self-reporting high or extremely high BP care confidence (self-efficacy) rose from 20% before to 90% after implementation.

**Implications for D&I Research**

Work system redesign with EHR-prompted workflows resulted in success tailoring and implementing a hypertension protocol for specialty clinic staff. Results suggest sound intervention reach, feasibility, and acceptability including improved BP re-measurement, follow up orders, patient-completed timely follow up, and staff self-efficacy.

**Primary Funding Source**

Peer reviewed QI funding

## Clinical (i.e., Primary, Specialty, Hospital, etc.) Care Settings

### S21 Diffusion of Excellence initiative: accelerating the implementation of best practices in America's largest integrated healthcare delivery system

#### Shereef Elnahal^1^, Andrea Ippolito^2^, Hillary Peabody^3^, Carolyn Clancy^1^

##### ^1^Office of the Under Secretary for Health, Organizational Excellence, US Department of Veterans Affairs, Washington, DC, 20005, USA; ^2^Office of Under Secretary for Health, Veterans Health Administration, Washington, DC, 20005, USA; ^3^Healthcare Performance Improvement and Innovation, Atlas Research, Washington, DC, 20005, USA

###### **Correspondence:** Shereef Elnahal

**Background**

The Veterans Health Administration (VHA) is America’s largest integrated healthcare system with over 1,700 sites of care, serving 8.76 million Veterans each year. Despite numerous accomplishments, VHA previously lacked a systematic mechanism for identifying and scaling best practices.

**Methods**

The model has five steps: ^(1)^ identify promising practices; ^(2)^ find the champions; ^(3)^ adapt and replicate; ^(4)^ establish consistency and standardize; and ^(5)^ sustain and improve. Three guiding principles were governance, process, and technology.

**Findings**

Launched in October 2015, this initiative has already resulted in over 280 adaptations of gold status practices at over 70 facilities nationwide. Key accomplishments include:

- Facilitated adaptation provided to over 15 facilities to adapt 12 practices.
- Identified select processes for national rollout
- Prioritized vulnerable populations facing mental health, economic, and chronic disease related challenges:
  - o One practice that includes the use of tablets for patients to complete screening tools from the waiting room reduced data entry time by 17 minutes per patient. This process improves care for Veterans with mental health conditions, as it increased screening for suicide risk from 50% to 90% and cut in half the time to document suicide risk.
  - o One practice brings Veterans together in social worker facilitated group visits to discuss advance care planning. 85% of all participants took concrete steps to develop more comprehensive care plans, especially important as patients with lower socioeconomic status are less likely to have Advance Directives.
  - o One practice leverages pharmacists to support the primary care team, saving primary care providers 15 minutes per new patient appointment. This practice could save up to 2,000 hours of primary care providers’ time if implemented just for patients with diabetes.

**Implications for D&I Research**

The VHA diffusion initiative model offers early evidence that it is an engaging solution that can scale best practices that serve vulnerable patient populations.

**Primary Funding Source**

Department of Veterans Affairs

### S22 Reducing disparities in a regional health improvement collaborative: a positive deviance approach

#### Randall Cebul^1^, Thomas Love^1,2^, Douglas Einstadter^1,3^, Shari Bolen^1,4^

##### ^1^Center for Health Care Research and Policy, Case Western Reserve University School of Medicine, Cleveland, OH, 44109, USA; ^2^Medicine and Epidemiology and Biostatistics, Better Health Partnership, Cleveland, OH, 44109, USA; ^3^MetroHealth Medical Center, Cleveland, OH, 44109, USA; ^4^Medicine, Better Health Partnership, Cleveland, OH, 44109, USA

###### **Correspondence:** Randall Cebul

**Background**

In northeast Ohio, our regional health improvement collaborative uses a positive deviance (PD) approach with public reporting to identify and disseminate best practices in primary care. PD identifies subgroups in a community whose strategies enable better solutions to problems than their peers, despite having similar challenges. We examine reductions in disparities in diabetes (DM) care and hypertension (HBP) control using the PD approach.

**Methods**

Observational study of disparities in DM care (four measures, over 7 years, by race/ethnicity) and HBP control (<140/90, over 2 years, by several social factors) among patients in diverse urban clinics. PD protocols were identified from clinic performance data, confirmed by interviews, and disseminated using practice coaching and biannual Learning Collaboratives. For DM, we modeled annualized region-wide changes in race/ethnicity gaps (highest minus lowest performing category). For HBP, we compared improvement in control across numerous disparities factors, including non-safety net vs. safety net clinics (SNC). For DM, 53 clinics reported DM achievement regularly across 2008-2014. In 2014, there were 34,185 DM patients: 46.3% non-white, 17.5% uninsured or Medicaid, and over 30% in the region’s lowest tertile for household income and education. For HBP, 35 clinics reported in both 2014 and 2015. In 2015, 42% of these clinics’ 122,740 patients were treated at SNCs.

**Findings**

For patients with DM in 2008, there was a 16.3 percentage point gap between white and Hispanic patients meeting all four care measures (achievement: 50.4% white, 47% black, and 34.1% Hispanic). In 2014, the gap was reduced to 4.4 points, an 11.9 point reduction. In a weighted regression, gap reduction was 1.72 points per year (95% CI: -2.74, -0.71; p = 0.002). For HBP, the top 9 improvers (2015 vs 2014) of 35 clinics were SNCs using PD with coaching. Together the 25 SNCs improved by 2.9 percentage points; non-SNCs improved by 0.5 point.

**Implications for D&I Research**

A PD approach in a primary care regional health improvement collaborative was associated with community-wide improvement and reductions in disparities in care and outcomes for important chronic conditions. Similar models for accelerating improvement should be tested in other regional improvement collaboratives that collectively serve more than 120 million Americans.

**Primary Funding Source**

The Robert Wood Johnson Foundation - Aligning Forces for Quality

### S23 Building synergy among national health system initiatives and a regional health improvement collaborative: a case study of the Cleveland Veterans Affairs medical center

#### Brook Watts^1,2^

##### ^1^ Informatics and Analytics, Louis Stokes Cleveland VA Medical Center, Cleveland, OH, 44106, USA; ^2^ Department of Medicine, Case Western Reserve University, Cleveland, OH, 44106, USA

###### **Correspondence:** Brook Watts

**Background**

The Veterans Health Administration, the largest integrated health system in the U.S., has made substantial efforts in recent years to ensure system-wide provision of high-quality ambulatory care. These efforts include full implementation of the Patient-Centered Medical Home, increased use of non-face-to-face encounters, and tools to support population management strategies that emphasize care coordination and quality improvement. On a local level, the Cleveland VA Medical Center (Cleveland VAMC) developed and implemented a variety of best practices to ensure high quality primary care. In addition, 13 VA clinics recently joined the main clinic at Cleveland VAMC in becoming members of Better Health Partnership (BHP), a northeast Ohio regional health improvement collaborative (RHIC) that publicly reports clinic-level data twice yearly.

**Methods**

Leadership at the Cleveland VAMC emphasizes the use of clinical process and outcome data for internal and external benchmarking as a foundational tool for quality improvement efforts. Local population health management tools and regular performance reports at the provider and clinic level were developed as a partnership between operational, analytic, and clinical staff. By participating with BHP in public reporting and twice yearly regional Learning Collaboratives, the Cleveland VAMC learns from and collaborates with health care “competitors” to improve care for the region’s residents with chronic medical conditions. Exceptional achievement (top decile performance in the region) is recognized by BHP with publicly awarded “Gold Stars”.

**Findings**

The Cleveland VAMC has maintained a “5-Star Quality” rating on internal VA benchmarking metrics since 2010. Public reporting of all fourteen Cleveland VAMC primary care clinics through BHP began in 2016, including results for 10,146 VA patients with diabetes and 40,910 patients with hypertension. All fourteen clinics received “Gold Star Practice” awards for care and/or outcome achievement in 2015.

**Implications for D&I Research**

Local initiatives in a hospital system synergized with national system-wide quality improvement efforts and a regional health improvement collaborative. Public reporting and the sharing of best practices with provider systems outside the VA will benefit the community and strengthen even high performing VA facilities.

**Primary Funding Source**

Department of Veterans Affairs

### S24 Applying lean principles to improve hepatitis C testing in VA primary care community clinics

#### Vera Yakovchenko^1,2^, Angela Park^3^, William Lukesh^3^, Donald R. Miller^1,2^, David Thornton^4^, Mari-Lynn Drainoni^1,2,5^, Allen L. Gifford^1,2,7^

##### ^1^Department of Health Law, Policy and Management, Boston University School of Public Health, Boston, MA, 02118, USA; ^2^Department of Veterans Affairs, VA HSR&D Center for Healthcare Organization and Implementation Research (CHOIR), Bedford, MA, 01730, USA; ^3^Boston VA Healthcare System, New England Veterans Engineering Resource Center, Boston, MA, 02132, USA; ^4^VA Boston Healthcare System, Boston, MA, 02139, USA; ^5^Department of Medicine, Boston University School of Medicine, Boston, MA, 02118, USA; ^7^Internal Medicine, Boston University School of Medicine, Boston, MA, 02215, USA

###### **Correspondence:** Vera Yakovchenko

**Background**

The proliferation of Lean and team-based quality improvement (QI) has been relatively unexplored at Department of Veterans Affairs (VA) community-based outpatient clinics (CBOCs), which often serve rural areas and socioeconomically underserved neighborhoods. Hepatitis C (HCV) testing is recommended for all those born between 1945-1965. Nevertheless, 42,008 (38%) VA New England 1945-1965 birth cohort Veterans have never been tested. We describe a primary care-based QI initiative using Lean principles, external facilitators, and local champions to increase HCV testing in CBOCs.

**Methods**

Four intervention and four matched control CBOCs across different healthcare systems were selected based on primary care volume (medium/large), HCV tested population (29.5-77.1%), and geographic location. The intervention consisted of: 1) collaborative on-site improvement sessions, 2) ongoing external facilitation, 3) champion-led Plan-Do-Study-Act cycles, and 4) audit and feedback. Using interrupted time series we analyzed pre- and post-intervention HCV testing rates, adjusting for clinic volume, implementation climate scores, and HCV prevalence.

**Findings**

Veterans with a first HCV test increased (p = 0.016) at intervention CBOCs by an average of 20.4% (4.4-36.2%) and without significant change (p = 0.204) at matched controls [5.3% (-0.5-15.1%)] over a three-month period. In the month prior to intervention, Lean intervention CBOCs were 8% of regional HCV testing and by intervention month three were 29%. Clinic staff participated in training sessions (N = 68) and responded (N = 45) to an organizational context instrument. At baseline, 43% of staff were satisfied with current HCV testing processes and 53% were familiar with QI. While 84% agreed that they have a sense of personal responsibility for improving patient care and outcomes only 31% agreed that they would receive appreciation for HCV testing Veterans. Higher improvement sites had more rapid uptake and integration of changes into workflow. The lowest baseline HCV testing performance site had the lowest implementation climate, and highest testing improvement with a 20-fold increase (1.9 to 38.1%) of newly tested.

**Implications for D&I Research**

This Lean and team-based multi-modal QI intervention activated HCV testing improvement in CBOCs. Future study will use Qualitative Comparative Analysis to identify the necessary context, organizational and interventional conditions conducive to adoption and sustainability before being scaled across CBOCs.

**Primary Funding Source**

Department of Veterans Affairs - QUERI

### S25 What does the facilitation implementation strategy look like in the real world?

#### Shawna Smith^1^, Julia Kyle^2^, Mark S Bauer^3,4^, Daniel Eisenberg^5^, Celeste Liebrecht^2,6^, Michelle Barbaresso^2,6^, Amy Kilbourne^2,7,8^

##### ^1^Internal Medicine, Division of General Medicine, University of Michigan, Ann Arbor, MI, 48109, USA; ^2^Psychiatry, University of Michigan Medical School, Ann Arbor, MI, 48109, USA; ^3^Center for Healthcare Organization and Implementation Research, VA HSR&D, Bedford/Boston, MA, 02130, USA; ^4^Department of Psychiatry, Harvard Medical School, Boston, MA, 02115, USA; ^5^Health Management and Policy, University of Michigan School of Public Health, Ann Arbor, MI, 48109, USA; ^6^Center for Clinical Management Research, VA Ann Arbor Health System, Ann Arbor, MI, 48109, USA; ^7^Dept of Veterans Affairs, VA Quality Enhancement Research Initiative (QUERI), Washington, DC, 20420, USA; ^8^VA Health Services Research and Development, VA Quality Enhancement Research Initiative, Washington, DC, 20420, USA

###### **Correspondence:** Shawna Smith

**Background**

Evidence-based collaborative care programs can improve physical and mental health outcomes. However, barriers to implementation of effective programs in community-based practices are significant. External facilitators (EF), or change agents outside of the study site, have been highlighted as a key factor in successful implementation. Yet, few studies have studied the content and impact of EF as it relates to implementation.

**Methods**

49 community-based practices participating in a health systems trial of implementation strategies to improve the uptake of a psychosocial intervention, Life Goals, were randomized to receive either External Facilitation (EF) or EF augmented with Internal Facilitation (EF + IF) for 6-12 months. The EF logged their tasks, categorizing mode, personnel interaction, duration, and primary focus of each task. We examine the content of EF tasks, describing predominant themes, and testing differences in content and quantity of tasks across sites receiving EF vs. EF + IF.

**Findings**

1,037 tasks were logged by the EF between January 2015 and July 2016. The EF logged a median of 28 minutes (IQR: 24-41) per site per month. 64% of interactions were done via email and 34% via phone, with email requiring a mean 6 minutes of EF time and phone calls 23 minutes. Interactions at EF sites (N = 564) involved site administrators (31%), supervisors (29%) and providers (20%); at EF + IF sites (N = 473), 70% of interactions were with the assigned IF. Mean interaction times did not differ across EF and EF + IF (t = 0.47, p = 0.64), however content did. EF sites focused more on education (EF: 55%; EF + IF: 45%), while EF + IF sites focused more on strategic tools (EF: 10%; EF + IF: 18%), including leveraging resources, marketing, and developing implementation plans, and reinforcement (EF: 26%; EF + IF: 36%).

**Implications for D&I Research**

Our uniquely-detailed purview of EF tasks provides insight into the mechanisms of EF as it impacts implementation efforts. Our results show that the focus of EF activity becomes more strategic when augmented with IF. Future work will link EF data to data collected by IFs to further describe differences in mechanisms of EF vs. EF + IF implementation strategies, and also explore site-level differences in EF interactions as they relate to implementation success and mediators of implementation success.

**Primary Funding Source**

National Institutes of Health - R01MH099898

### S26 Lesson learned from NCI SPRINT entrepreneurs: commercializing a tobacco treatment for cancer center implementation

#### Elyse Park^1^, Giselle Perez^1^, Jamie Ostroff^2^

##### ^1^MIHP, MGH, Boston, MA, 02114, USA; ^2^Psychiatry, Memorial Sloan Kettering Cancer Center, New York, NY, 10022, USA

###### **Correspondence:** Elyse Park

**Background**

As clinical researchers investigating the integration of evidence-based, tobacco treatment into cancer care, we participated in an NCI Dissemination and Implementation Research Program initiative beginning in July 2016, Speeding Research-tested INTerventions (SPRINT). SPRINT’s trains researchers in the business of real-world transformation of behavioral interventions into commercially viable and scalable products or services. We have worked towards creating and refining a business plan for our promising intervention. Our goal is to market tobacco treatment, as a 3-part product, to a variety of buyers (i.e., hospitals, health care systems, employers).

**Methods**

The SPRINT course consists of didactic learning, interactive presentations, expert consultation, collaborative learning and completion of targeted interviews focusing on our potential customers’ preferences and needs. We were tasked to complete 30 interviews with potential "end users" (e.g., patients, clinicians, administrators, insurers, and businesses). Our initial value propositions, are as follows: 1) Providers and patients need a detailed tobacco treatment manual/guide; 2) Cancer patients have unique tobacco treatment needs; 3) Oncology nurses will be eager to deliver tobacco treatment; and 4) There are specialized training needs for tobacco treatment in cancer patients.

**Findings**

To date we have completed 20 interviews, all of which have played vital information supporting or disproving our value propositions. We developed a Value Proposition Canvas to assess interest in our products (e.g., manuals, certification and training), perceptions of customer “Pains” (reducing no shows) this product could address, and customer “Gains” (engaging patients in tobacco treatment) that our product would achieve. We also identified three types of customer archetypes (oncology providers, tobacco treatment specialists, and cancer patients) representing our potential end users. Currently, we are exploring channel distributions (e.g., publishing and training companies) as well as payment and reimbursement venues. Primary interview themes will be presented.

**Implications for D&I Research**

Lessons learned from our training experience- both its process and results- will guide our ability to transform our research-tested intervention into a marketable product. Complementary to traditional behavior change theory and strategies, business concepts and methods, introduced through SPRINT and explored through a series of interviews, can shape the translation of behavioral intervention into real world settings.

**Primary Funding Source**

National Institutes of Health - NCI SPRINT initiative

### S27 Dissemination and implementation in health systems: more GPS than self-driving car

#### Sarah Greene^1^, Michael Parchman^2^, Brian Austin^2^, Eric Larson^2^

##### ^1^Research, Health Care Systems Research Network, Seattle, WA, 98115, USA; ^2^MacColl Center for Health Care Innovation and Senior Investigator, Group Health Research Institute, Seattle, WA, 98101, USA

###### **Correspondence:** Sarah Greene

**Background**

Effective dissemination of research findings into practice is a critical element of the research lifecycle, but one that is often constrained by lack of resources, time, and/or skill. Moreover, spreading evidence into organizations requires substantial effort, as implementation is rarely a turn-key process. Overcoming these barriers and enhancing availability and usability of evidence is a critically important step in realizing the promise of true learning healthcare systems. Using the example of an embedded research center in a care delivery system, we explored the barriers and potential facilitators to more effective dissemination and implementation (D&I).

**Methods**

Through key informant interviews and an environmental scan, we explored the barriers and facilitators to researchers' willingness and ability to engage in D&I activities beyond conference presentations and journal publications. We assessed the potential business model(s) for effective D&I, and also discussed with interviewees whether there is a moral imperative to engage in D&I. The environmental scan identified case studies of exemplar entities that sustained their research through strategic D&I activities.

**Findings**

Through 28 semi-structured interviews, we identified a range of challenges and opportunities related to researchers' dissemination and implementation capabilities. Notably, some researchers felt compelled to intensify their D&I skills and activities, whereas others felt that good science and publications were sufficient end products. Challenges impeding D&I included lack of funding, lack of protected time for building connections and nurturing relationships with health system leaders, and incomplete understanding of the market potential for research products. Use of digital and social media was identified as an opportunity, but many researchers are not proficient in these newer approaches. Fifteen exemplar organizations were included in the environmental scan, and illustrate a range of business models and approaches to supporting translation of research into practice.

**Implications for D&I Research**

Dissemination and implementation of research results often amounts to an "unfunded mandate" for many researchers. Moreover, many would benefit from training on how to effectively communicate their results to healthcare decision-makers, policymakers, and other stakeholders. The entire research community would benefit from addressing the structural, interpersonal, financial, and technical aspects that invigorate and incentivize more effective D&I.

**Primary Funding Source**

GHRI Director's Development Fund

### S28 Implementation of an integrated care management program in community pharmacies: identifying barriers and employing implementation strategies

#### Stefanie Ferreri^1^, Chris Shea^2^, Megan Smith^3^, Kea Turner^4^

##### ^1^Division of Practice Advancement and Clinical Education, University of North Carolina Eshelman School of Pharmacy, Chapel Hill, NC, 27599, USA; ^2^Health Policy and Management, The University of North Carolina at Chapel Hill, Chapel Hill, NC, 27599, USA; ^3^Pharmacy Practice, University of Arkansas for Medical Sciences College of Pharmacy, Little Rock, AR, 72205, USA; ^4^Health Policy and Management, University of North Carolina Gillings School of Global Public Health, Chapel Hill, NC, 27599, USA

###### **Correspondence:** Stefanie Ferreri

**Background**

Preliminary evidence and expert guidance suggest integrated care management (ICM) programs in community pharmacies contribute to improved outcomes and lower cost of care. However, existing payment models prioritize drug dispensing rather than integrated care services. In this study, we identified and categorized barriers to implementing ICM services among a network of community pharmacies and documented implementation strategies employed to assist uptake.

**Methods**

The Community Pharmacy Enhanced Services Network (CPESN) of North Carolina focuses on implementation of a payment model to support delivery of enhanced services. At launch, 123 community pharmacies joined CPESN. We conducted interviews with pharmacists representing 73 pharmacies to identify implementation barriers within the ICM program: ^(1)^ Initiating; ^(2)^ accessing internal information; ^(3)^ accessing external information; ^(4)^ scheduling patients; ^(5)^ performing patient interviews; ^(6)^ developing assessment; ^(7)^ developing a care plan; ^(8)^ documenting; ^(9)^ coordinating care; and ^(10)^ performing follow-up. We also conducted 23 interviews with CPESN staff and collaborators who were employing implementation strategies to assist pharmacies. We used a semi-structured interview guide informed by Proctor et al.’s (2013) recommendations for reporting implementation strategies and Powell et al.’s (2015) implementation strategy compilation.

**Findings**

Most pharmacists reported barriers around “Initiating” and “Documenting”. Many pharmacists lacked a planning process for incorporating new services, underestimated resources needed, and/or did not ensure timely availability of resources. The most commonly reported barriers were around adequate staffing and time. Also, documentation in the web-based platform was problematic due to unclear requirements, poor system design, time, and system connectivity/responsiveness. Regarding implementation strategies, several strategies from the Powell et al. compilation were reported, such as conducting educational outreach and audit and feedback. The strategies have been focused on utilizing staff time more efficiently, facilitating documentation in the web-based platform, and monitoring performance.

**Implications for D&I Research**

Our project highlights challenges with implementing new care processes within a complex program. Identifying barriers within an ICM program facilitates targeted interventions for pharmacists’ needs; however, systematic evaluation of each strategy is needed. Finally, documenting implementation strategies promotes more efficient use of implementation resources and reveals barriers not currently being addressed.

**Primary Funding Source**

Centers for Medicare and Medicaid Services - CMS Demonstration Grant

### S29 Implementation strategies for patient care services in community pharmacy: a systematic review

#### Jennifer Bacci^1^, Kyle Bigham^1^, Geoffrey Curran^2^, Stefanie Ferreri^3^, Caity Frail^4^, Cory Hamata^1^, Terry Jankowski^5^, Wendy Lantaff^6^, Melissa Somma McGivney^7^, Margie Snyder^6^

##### ^1^Pharmacy Practice, University of Washington School of Pharmacy, Seattle, WA, 98195, USA; ^2^Department of Pharmacy Practice, University of Arkansas for Medical Sciences, Little Rock, AR, 72205, USA; ^3^Division of Practice Advancement and Clinical Education, University of North Carolina Eshelman School of Pharmacy, Chapel Hill, NC, 27599, USA; ^4^Pharmacy Practice, University of Minnesota College of Pharmacy, Minneapolis, MN, 55455, USA; ^5^Library Science, University of Washington, Seattle, WA, 98195, USA; ^6^Pharmacy Practice, Purdue University College of Pharmacy, Indianapolis, IN, 46202, USA; ^7^Pharmacy Practice, University of Pittsburg School of Pharmacy, Pittsburg, PA, 15261, USA

###### **Correspondence:** Jennifer Bacci

**Background**

Studies have identified the positive impact of patient care services in community pharmacy; yet, widespread implementation remains limited. Identifying the most effective implementation strategies is critical to sustaining evidence-based patient care in this setting. This scoping systematic review aims to describe strategies used to ^(1)^ implement and ^(2)^ evaluate the implementation of patient care services in community pharmacies.

**Methods**

PubMed/ MEDLINE, EMBASE, and International Pharmaceutical Abstracts were searched until March 9, April 20, and April 5, 2016, respectively. *Innovations in Pharmacy, Implementation Science,* and reference lists of excluded articles were hand searched. The primary investigator reviewed each article’s abstract to assess eligibility for full-text review. At least 2 investigators conducted full-text reviews. Discrepancies were resolved by a third investigator. To be included, articles had to ^(1)^ be written in English and published in 1985 or later; ^(2)^ describe a peer-reviewed empirical study or practice experience implementing a patient care service in a community pharmacy; and, ^(3)^ describe the strategies used to implement and/ or evaluate the implementation of the service. Data are being extracted from each article by 2 investigators using a standardized form, including pharmacy type(s), patient care service type(s), workflow and financial models, pharmacy staff roles, implementation strategies, implementation outcomes, evaluation method, and conceptual model/ framework. Implementation strategies are being categorized using terminology from the Expert Recommendations for Implementation Change (ERIC) study by Waltz and colleagues.

**Findings**

Of the 3,046 articles retrieved, 237 were included. The majority describe implementation of medication management, disease state management education, and risk reduction services in the US, Europe, and Australia. Preliminary results indicate an emphasis on adapting and tailoring to local context and training and educating stakeholders as implementation strategies and qualitative and process evaluation. Final results will be completed in October 2016.

**Implications for D&I Research**

This review is one of the first efforts to comprehensively identify implementation strategies for patient care services in community pharmacies and is a critical step in identifying the most effective implementation approaches. The results of this work will enable implementation scientists and clinicians to refine and test strategies that result in sustainable and scalable patient care service implementation.

### S30 Testing implementation strategies for anticoagulation improvement in clinical pharmacy clinics: a qualitative study

#### Megan McCullough^1,2^, Chris Gillespie^1^, Beth Ann Petrakis^1^, Ellen Jones^3^, Angela Park^4^, Carol VanDeusen Lukas^5,6^, Adam Rose^1,7^

##### ^1^Center for Healthcare Organization and Implementation Research (CHOIR), VA Health Services Research & Development, Bedford, MA, 01730, USA; ^2^Department of Health Law, Policy and Management, Boston University School of Public Health, Boston, MA, 02118, USA; ^3^Pharmacy, Central Western Massachusetts VA Healthcare System, Leeds, MA, 01053, USA; ^4^Boston VA Healthcare System, New England Veterans Engineering Resource Center, Boston, MA, 02130, USA; ^5^Research, VA Boston Healthcare System, Center for Healthcare Organization and Implementation Research, Boston, MA, 02130, USA; ^6^Health Policy and Management, Boston University School of Public Health, Boston, MA, 02118, USA; ^7^Internal Medicine, Boston University Medical Center, Boston, MA, 02119, USA

###### **Correspondence:** Megan McCullough

**Background**

Implementation science (IS) has recently concentrated efforts on assessing what implementation strategies are effective and in what circumstances. In complex interventions, a “bundle” of implementation strategies are often applied. In a four-year pharmacist-focused Anticoagulation Care Improvement Initiative (ACCII), we implemented a strategy bundle (audit and feedback, blended internal-external facilitation, small tests of cyclical change, and ongoing consultation) to improve anticoagulation across 8 Veterans Health Administration (VHA) medical centers in a single region. This study describes the significance and impact of these combined strategies on successful implementation.

**Methods**

We conducted an average of 50 semi-structured interviews with all frontline ACC staff annually for 4 years with a special focus on implementation strategies. Interviews were conducted with the pharmacy leadership twice (N = 22) and the External Facilitation Team (delivered intervention) once (N = 5). Analysis drew deductively on Promoting Action on Research Implementation in Health Services (PARIHS) as well as an emergent thematic analysis. Analysis focused on identifying factors related to the impact and effectiveness of strategies employed.

**Findings**

Sites with greater improvement: 1) Integrated and used all IS strategies. 2) Had support from managers so full implementation of strategies could occur. 3) Spread strategies throughout entire clinical team (i.e., made quality improvement everybody’s job) as opposed to having a designated person. Sites with less successful implementation of the ACC II had less leadership support and lower engagement but most important they divided the bundle of strategies (i.e., pursued some but not all of them).

**Implications for D&I Research**

While the value of bundling IS strategies has been recognized, this study suggests how and why bundling can be effective in successful implementation as well as where it might not. Our findings can help guide researchers who seek to choose and then incorporate a bundle of implementation strategies. This study contributes to a growing literature regarding if and how bundles of implementation strategies impact successful implementation.

**Primary Funding Source**

Department of Veterans Affairs - Research Grant from the Department of Veterans Affairs

### S31 An implementation research model for pharmacy: application of the Consolidated Framework for Implementation Research (CFIR) to community pharmacy

#### Sarah J. Shoemaker^1^, Geoffrey Curran^2^, Jeremy Thomas^2^, Benjamin Teeter^2^, Holly Swan^1^

##### ^1^US Health, Abt Associates, Cambridge, MA, 02138, USA; ^2^Department of Pharmacy Practice, University of Arkansas for Medical Sciences, Little Rock, AR, 72205, USA

###### **Correspondence:** Sarah J. Shoemaker

**Background**

Pharmacists are providing direct patient care (e.g., medication therapy management) and delivering important prevention and screening (e.g., immunizations, rapid HIV testing) interventions. Community pharmacies are an increasingly important health care setting with opportunities for improving quality and safety, yet little is understood about determinants of implementation. Given community pharmacy’s unique features for consideration in implementation research, we developed a model for implementation research for this setting based on the Consolidated Framework for Implementation Research (CFIR).

**Methods**

We conducted a critical review examining literature on services and interventions provided in community pharmacies, including MTM services, rapid HIV testing, and immunizations provided in community pharmacies. We scanned select titles and abstracts to identify implementation studies or studies that sought to understand barriers and facilitators to implementing these types of services in community pharmacies. We found few systematic implementation studies in these settings. To synthesize findings and develop a model, we chose the CFIR because of its ability to situate potential implementation determinants across a wide range of constructs.

**Findings**

While *Intervention Characteristics* depend on individual interventions, of note here are “relative advantage” and “complexity”. The former because implementation of services can pose a cost-benefit challenge where dispensing is the primary role and the latter because of the challenge of integrating services into the dispensing workflow. In terms of *Outer Setting*, pharmacies are subject to external policy and incentives (e.g., CMS star ratings, payor incentives) but to a lesser extent than other health settings. For *Inner Setting*, important structural characteristics include pharmacy type (e.g., chain, independent, safety net), extent of “patient-centeredness”, and relationships with schools of pharmacy-- which facilitate social networks, communications, and “in-kind” staffing (e.g., students, residents). Key *Characteristics of Individuals* include training, preparedness, and self-efficacy of the pharmacist for providing new services (e.g., MTM). Finally, *Process* constructs of note from pharmacy implementation studies include the importance of champions/active local change agents.

**Implications for D&I Research**

As pharmacists’ roles in health care expand, models to inform implementation research in community pharmacy (and other) settings are crucially needed. Additionally, further application of the CFIR to other healthcare settings expands the field’s understanding of the utility of the framework.

### S32 Adaptation of a motivational interviewing intervention in community pharmacies: application of the Wiltsey Stirman framework to characterize modifications in a 4-site implementation demonstration study

#### Benjamin Teeter^1^, Jeremy Thomas^1^, Geoffrey Curran^1^, Appathurai Balamurugan^2^

##### ^1^Department of Pharmacy Practice, University of Arkansas for Medical Sciences, Little Rock, AR, 72205, USA; ^2^Center for Health Advancement, Arkansas Department of Health, Little Rock, AR, 72205, USA

###### **Correspondence:** Benjamin Teeter

**Background**

As community pharmacies expand their role in healthcare through the implementation of cognitive services into their dispensing workflows, identifying the different modifications made to these services for implementation and the impact of these modifications are important to determining their success or failure. Previous research by Wiltsey Stirman and colleagues (2013) identified 4 types of contextual modifications, 12 types of content modifications, and 7 levels at which modifications occur, all of which have the potential to impact the desired benefits of the intervention. This study seeks to apply their framework in the community pharmacy setting to determine the modifications that occur during implementation of cognitive services.

**Methods**

In a 4-site demonstration project, pharmacists were trained to provide a brief Motivational Interviewing (MI) intervention to at least 50 patients who were non-adherent to antihypertensive medications. Training included a three-hour online course in MI and in-pharmacy training on identifying eligible patients and documenting the intervention. Observations and semi-structured interviews took place in 4 community pharmacies that implemented the MI intervention. Interviews covered modifications to the process of identifying eligible patients, MI interventions, and documenting the intervention. Data was coded using Wiltsey Stirman and colleagues’ framework.

**Findings**

Contextual modifications were made to the format of the intervention (e.g., telephone instead of in-pharmacy). All 4 pharmacies reported conducting at least some of their interventions via telephone. The most common content modification was ‘Loosening the Structure’ (e.g., cancelling use of computer alerts) followed by ‘Drifting or Departing’ (e.g., stopped MI with defensive patients). Although less frequent, ‘Adding Elements’ (e.g., reminder cards) and ‘Repeating Elements’ (e.g., identification of patients) were also mentioned. Modifications to the MI intervention typically occurred at the provider/facilitator level. Interestingly, modifications seemed to be made to fit the pharmacists’ needs, not the needs of their patients.

**Implications for D&I Research**

This study demonstrates the utility of Wiltsey Stirman and colleagues’ framework in the pharmacy setting. In contrast to previous research, individual level content modifications were uncommon. Data from this study will guide future research within a larger sample of pharmacies in an effort to determine the modifications that are detrimental and beneficial to patient outcomes and sustainability of services.

### S33 Evidence supporting clinician acceptance of a standardized handoff process: findings from a hybrid effectiveness-implementation study of operating room to intensive care unit handoffs

#### Meghan Lane-Fall^1,2,3^, Rinad Beidas^2,4^, Laura Di Taranti^1^, Sruthi Buddai^1^, Enrique Torres Hernandez^5^, Jerome Watts^6^, Lee Fleisher^1,2^, Frances Barg^1,7^

##### ^1^Anesthesiology and Critical Care, Perelman School of Medicine, University of Pennsylvania, Philadelphia, PA, 19104-4865, USA; ^2^Leonard Davis Institute of Health Economics, University of Pennsylvania, Philadelphia, PA, 19104, USA; ^3^Center for Healthcare Improvement and Patient Safety, Perelman School of Medicine, University of Pennsylvania, Philadelphia, PA, 19104, USA; ^4^Psychiatry, University of Pennsylvania, Philadelphia, PA, 19104, USA; ^5^School of Science, Engineering and Technology, St. Mary's University, San Antonio, TX, 78228, USA; ^6^Haverford College, Haverford, PA, 19041, USA; ^7^Family Medicine and Community Health, University of Pennsylvania, Philadelphia, PA, 19104, USA

###### **Correspondence:** Meghan Lane-Fall

**Background**

Hospitalized patients whose care is transferred from one healthcare team to another experience a “handoff”, during which patient information and accountability are transferred. After implementing a standardized handoff protocol for patients being transferred from a surgical operating room (OR) to the intensive care unit (ICU), we sought to determine clinician acceptance of the new process.

**Methods**

As part of the Handoffs and Transitions in Critical Care (HATRICC) study, we conducted interviews, focus groups, collected surveys, observed patient handoffs, and surveilled the health system’s event reporting system from 2014 to 2016. We defined “process adherence” as following our standardized handoff protocol's steps in a pre-specified order. Qualitative data were analyzed with a grounded theory approach while quantitative data were analyzed with descriptive and bivariate statistics.

**Findings**

We conducted 109 interviews, 8 focus groups (71 participants), and collected 445 responses across 3 surveys from clinicians participating in OR to ICU handoffs, including physicians, nurses, and advanced practice providers. In interviews and focus groups before the intervention, a “perfect handoff” was variably described, but this definition coalesced afterward to become consistent with the new handoff protocol. In surveys, 143/152 (94.1%) and 136/152 (89.5%) respondents found the new process to be appropriate and acceptable, respectively. 125/160 (78.1%) respondents said that they usually or always used the new process in their handoffs. 107/156 (68.7%) said that the new process made patient care “better” or “much better”. We observed 61 patient transfers before and 156 transfers after standardized handoff implementation. In the postintervention period, 156/156 transfers included an observable handoff, compared to 53/61 (86.9%) before the intervention (p < 0.001). Clinicians adhered to the new handoff process in 110/156 (70.5%) handoffs. Review of the hospital’s event reporting system revealed safety reports including the term “HATRICC” (study acronym) used as both a noun and a verb, indicating familiarity with the process.

**Implications for D&I Research**

Multiple data sources may be used to assess clinician acceptance of a process change. In this study, we demonstrated through interviews, focus groups, surveys, direct observation, and event report review that clinicians were accepting of a standardized OR to ICU handoff process.

**Primary Funding Source**

Safety Scientist Career Development Award

### S34 Defying expectations for care management practice uptake: a mixed methods analysis of positive deviants in a national sample of physician organizations

#### Isomi Miake-Lye^1^, Tanya Olmos^2^, Emmeline Chuang^1^, Hector Rodriguez^3^, Gerald Kominski^4^, Becky Yano^1,5^, Stephen Shortell^6^

##### ^1^Health Policy and Management, UCLA Fielding School of Public Health, Los Angeles, CA, 90024, USA; ^2^Health Policy and Management, University of California, Los Angeles, San Fernando, CA, 91340, USA; ^3^Health Policy and Management, University of California - Berkeley, School of Public Health, Berkeley, CA, 94720, USA; ^4^Department of Health Policy and Management, UCLA Center for Health Policy Research, Los Angeles, CA, 90272, USA; ^5^HSR&D Center for the Study of Healthcare Innovation, Implementation & Policy (CSHIIP), VA Greater Los Angeles Healthcare System, Sepulveda, CA, 91343, USA; ^6^School of Public Health, University of California, Berkeley, Berkeley, CA, 94720, USA

###### **Correspondence:** Isomi Miake-Lye

**Background**

Evidence-based practices are often slow to diffuse from the settings in which they were initially developed. Given the differing contexts of these later adopter organizations, new strategies may be needed to support adoption. This study seeks to identify organizational characteristics associated with non-adoption of care management practices (CMPs) for diabetes and to examine the role of culture, leadership, and organizational priorities in non-adopters and positive deviants.

**Methods**

In this explanatory sequential mixed methods study, we first use quantitative analyses to identify organizational characteristics associated with non-adopter status in cross-sectional data from the National Survey of Physician Organizations (NSPO3, 2012-2013; n = 1,328), a nationally representative sample of physician organizations. We then conducted semi-structured interviews with key stakeholders (i.e., primary care provider, nurse, administrator) in a purposive sample of positive deviant and non-adopter organizations. Qualitative directed content analysis builds from this work to explore the role of culture, leadership, and organizational priorities in the adoption decisions for diabetes CMPs among non-adopter and positive deviant organizations.

**Findings**

We identified organizational characteristics including using electronic medical records, using quality improvement systems, making investments in quality of care, having physician ownership, and being in California. These factors were then used as sampling criteria in the qualitative phase. Two non-adopter sites and two positive deviant sites were included in interviews. All sites shared similarities in culture that resembled the laggard characteristics described by Diffusion of Innovation, including aversion to change. The main differences identified in qualitative analyses between non-adopter and positive deviant sites were if they considered diabetes management to be within their scope of practice and if they described support from outside organizations with CMPs.

**Implications for D&I Research**

Non-adopters and positive deviants had cultural similarities in addition to being similar on quantitative measures. While non-adopters may require more outside support to adopt diabetes CMPs, they may also be non-adopters for key strategic reasons, such as focusing solely on specialty care. Lessons learned from positive deviants may be key in building strategies to combat variations in care, and more attention to these organizations is warranted.

**Primary Funding Source**

Agency for Healthcare Research and Quality - AHRQ R36 HS024176-01; National Study of Physician Organizations III was funded by the Robert Wood Johnson Foundation (Award No. 68847)

### S35 Using implementation theory to evaluate the impact of technology on nurses’ knowledge and use of best practices in acute care

#### Mary Hook

##### Center for Nursing Research and Practice, Aurora Health Care, Milwaukee, WI, 53233, USA

**Background**

Advances in health information technology (HIT) and the use of electronic clinical decision support (CDS) tools to support nurses to know and use evidence-based practices (EBP) hold great promise, but relatively untested in acute care.

**Methods**

This pre/post mixed methods study was conducted to evaluate the impact of embedding EBP recommendations into policy and the electronic health record (EHR) to support nurses to know and use best practices to improve patient outcomes. The Dissemination of Evidence-based Policy Framework (Dobson, Brownson, & Weiss, 2012) was adapted and guided the study, proposing that the impact of an EBP innovation is influenced by how it is deployed. The study was conducted with consenting inpatient nursing units (N = 28 units) from 3 diverse facilities where the policies and technology were deployed. Non-participant observations, audits, nurse surveys, patient surveys, process and outcome metrics were gathered to describe context, nurse knowledge, use of EBP behaviors, and achievement of nurse-sensitive patient outcomes. A multimodal implementation intervention was delivered with audit/feedback, optimization training, and support for unit-based implementation related to 6 nurse-sensitive phenomena: pain, falls, pressure ulcers, medication adherence, delirium, and depression/suicide over a 6 month period.

**Findings**

Baseline findings revealed a supportive culture, functioning technology, with gaps in knowledge (N = 536, Mean Score = 56.3% correct, SD 8.4) and use of EBP practices using dissemination-based deployment conditions. Baseline results were used to create the feedback-based optimization training curriculum. Training Sessions (n = 57) were attended by 89% of staff and 100% of leaders with high ratings for goal achievement and commitment to implementation. Units were observed to have limited capacity to monitor and maintain EBP behaviors during the implementation phase. Post assessment revealed some improvement in knowledge (N = 523, Mean Score = 61.8% correct, SD, 8.8, *p* < 0.001) and use of EBP behaviors with little change in patient outcomes.

**Implications for D&I Research**

Gaps in knowledge and use of technology-supported EBP were identified and addressed with a multimodal implementation intervention. Despite high training participation and commitment, units had limited capacity to implement and maintain best practices over time. These findings suggest that the impact of EBP-based technology will be limited when deployment is based on dissemination training alone.

**Primary Funding Source**

US Army Medical Research and Materiel Command (USAMRMC) Grant No. W81XWH-13-1-0034

### S36 Integrating emergent mhealth apps into pediatric practice: a mixed methods implementation project

#### Linda Fleisher^1,2^, Alexander Fiks^3^, Katie Halkyard^1^, Rachel Gruver^1^, Emily Sykes^1^

##### ^1^Research Institute, Children's Hospital of Philadelphia, Philadelphia, PA, 19104, USA; ^2^Population Science, Fox Chase Cancer Center, Philadelpia, PA, 19015, USA; ^3^General Pediatrics, University of Pennsylvania, Philadelphia, PA, 19066, USA

###### **Correspondence:** Linda Fleisher

**Background**

The explosion of digital/mHealth tools for consumers coupled with their increased integration into health care systems has prompted the need for systematic organizational processes to evaluate and determine which tools should be provided or recommended. The iAPP (Integrating Apps into Pediatric Practice) initiative was created to develop a strategy to integrate evidence-based health apps for patients and families into pediatric care.

**Methods**

This 2-year project includes: an implementation framework-guided environmental scan of leading pediatric hospitals, parent surveys , provider surveys, development of a decision framework and governance process, and validation through a number of strategically selected pilot projects. This abstract focuses on the environmental scan (including surveys and interviews) results and the resulting implementation tools (decision tree, driver diagram, and process documents) to guide mHealth integration. Leading pediatric hospitals (N = 7) were recruited based on their reputation in innovation and mHealth. The appropriate contact (Director-level) completed a brief online survey prior to the interview. The interview guide, developed based on four implementation frameworks (e.g. TAM, Rodgers’ Innovation) included questions on strategic leadership, culture, regulatory, economic, technical, legal and governance factors within the context of mHealth tools for patients and families.

**Findings**

Findings from both the survey and interviews highlighted that although these hospitals are entering the digital health/mobile health space (90% using digital health tools), only 3 had policies either in development/implementation or relied on external organizations (e.g. HIMSS) for guidance. The most frequent topics for these mHealth tools were patient education and chronic disease management and most are deployed through the app store or institutional websites. Respondents indicated that the 3 top factors to determining whether to use a 3^rd^-party app were cost, credibility and level of clinician support.

**Implications for D&I Research**

This presentation focuses on the findings of this environmental scan that have applicability to the integration of mHealth into practice as well as our resulting decision making and implementation process tools addressing the evidence of efficacy, perceived clinical utility/impact on quality, interest of clinicians and families in using a particular app, the fit of the app in workflows, age appropriateness, and sensitivity to culture, cost and the potential for adverse consequences/safety.

**Primary Funding Source**

Pediatric Chair's Initiative

### S37 Promoting change in the genitourinary care of postmenopausal women through clinician education and electronic health record tools: a cluster randomized trial

#### Kimberly Vesco^1,2^, Kate Beadle^3^, Joanna Bulkley^1^, Ashley Stoneburner^1^, Michael Leo^1^, Amanda Clark^1,2^

##### ^1^Science Programs Department, Kaiser Permanente Center for Health Research, Northwest, Portland, OR, 97227, USA; ^2^Obstetrics & Gynecology, Northwest Permanente, Clackamas, OR, 97015, USA; ^3^Obstetrics & Gynecology, Kaiser Permanente, Northwest, Portland, OR, 97227, USA

###### **Correspondence:** Kimberly Vesco

**Background**

Data from our health care system (2011-2012) suggested that the genitourinary syndrome of menopause (GSM), an easily treated condition that progresses in severity with age, was underdiagnosed in our patient population (observed 4%, expected 20-45%). To promote greater detection and treatment of GSM, we created a clinician-focused intervention which included a suite of evidence-based electronic health record (EHR) tools and clinician education. We conducted a cluster-randomized trial to test the efficacy of the intervention.

**Methods**

We randomized Primary Care (PC) and Gynecology (GYN) clinics to intervention (8 PC, 3 GYN) or control (8 PC, 3 GYN). From September to November 2014, we provided training about GSM diagnosis, treatment, and the EHR tools through face-to-face presentations at each intervention clinic and through an on-line video. Control clinicians received no training or notification about the tools. Our primary outcome was the proportion of well visits with GSM-related diagnoses and prescriptions from 11/15/14 through 11/15/15. We also assessed use of the electronic tools. There was PC and GYN departmental support for the intervention but no prioritization within the health care system to drive new performance measures or incentives for change related to GSM care.

**Findings**

There were 199 intervention and 208 control clinicians who performed 15,062 well visits for women aged 55 and older. Among intervention clinicians, 107 (53.7%) completed educational training. Intervention clinicians were more likely (p < .001) to use the Smart Set (2.8 vs 0.1 uses) and Smart Text (1.1 vs 0.1 uses). However, the proportion of visits that included a GSM-related diagnosis (9.2% vs 8.2%) or prescription (6.5% vs 5.8%) did not differ between study arms. There was a significant interaction for PC and Gyn, suggesting an increase in GSM diagnosis in Gyn but not PC intervention clinics (OR = 1.57, p = .01).

**Implications for D&I Research**

EHR tools are more likely to be used if clinician education is included as part of their implementation. However, without simultaneous departmental and organizational initiatives to promote, enforce, or sustain a clinical practice change, the overall efficacy of EHR tools and education alone for improving patient care is limited and competing patient care priorities may take precedence.

**Primary Funding Source**

Other (please specify below) - North American Menopause Society & Pfizer Independent Grant for Learning & Change #10319

### S38 Provider-developed clinical decision support and education to optimize and sustain timely and accurate treatment of hypoxic ischemic encephalopathy in the neonatal intensive care unit

#### Joan Smith^1^, Christopher Smyser^2^, Maggie Wolf^1^, Shamik Trivedi^3^, Brian Hackett^4^, Rakesh Rao^4^, F. Sessions Cole^4^, Rose McGonigle^5^, Ann Donze^6^, Enola Proctor^7^, Amit Mathur^8^

##### ^1^Nursing, St. Louis Children's Hospital, St. Louis, MO, 63110, USA; ^2^Neurology, Washington University School of Medicine, St. Louis, MO, 63110, USA; ^3^Neonatal-Perinatal Medicine, Washington University School of Medicine, St. Louis, MO, 63110, USA; ^4^Pediatrics and Division of Newborn Medicine, Washington University School of Medicine, St. Louis, MO, 63110, USA; ^5^Newborn Intensive Care Unit, St. Louis Children's Hospital, St. Louis, MO, 63110, USA; ^6^Nursing/Newborn Intensive Care Unit, St. Louis Children's Hospital, St. Louis, MO, 63110, USA; ^7^George Warren Brown School of Social Work, Washington University in St. Louis, St. Louis, MO, 63130, USA; ^8^Pediatrics and Newborn Medicine, Washington University School of Medicine, St. Louis, MO, 63110, USA

###### **Correspondence:** Joan Smith

**Background**

Neonatal brain injury due to hypoxic ischemic encephalopathy (HIE), or birth asphyxia, is the leading cause of all neonatal deaths worldwide. Therapeutic hypothermia (TH) is standard of care for moderate to severe HIE. However, significant practice variation and the potential for inappropriate treatment persist. Timely identification for appropriate treatment is critical to survival, outcome, and cost. Although knowledge on the use of TH is substantial, it is not always aptly applied. Using the Promoting Action on Research Implementation in Health Services (PARiHS) framework and human factors principles, our interdisciplinary team of providers, nurse/physician leaders, informatics analysts/specialists and family partners developed and deployed provider education and an evidence-based electronic clinical decision support (CDS) tool in the electronic health record (EHR) to reduce practice variation and improve teamwork, consistency and appropriateness of treatment.

**Methods**

A pre-post study design was used and an interdisciplinary team formed to test the CDS integration into provider workflow. Prior to implementation, we conducted usability testing with a standard ‘think aloud’ approach for iterative evaluation and provider education using a 10-item pre-post knowledge test. Following implementation, we measured provider satisfaction with the Computer System Usability Satisfaction Questionnaire (CSUQ; score range on each item: 1 = strongly disagree to 7 = strongly agree) and fidelity with an electronically abstracted adherence score. Appropriateness of treatment was determined by the percentage of infants treated with TH across the mild, moderate and severe encephalopathy categories.

**Findings**

Usability testing identified and corrected many issues in the functionality of the CDS prior to implementation. Provider knowledge increased from a median total score of 44.44 at baseline to 74.33 at follow-up (p < 0.001). Overall, providers were extremely satisfied with the tool and found it to be helpful (mean item score, 6.4). Fidelity with the CDS was high (>90%) with 98% of the infants being appropriately treated with TH.

**Implications for D&I Research**

Implementation science informed deployment of a provider-developed CDS tool integrated into clinical workflow and provider education decreased practice variation and improved interdisciplinary teamwork, consistency and appropriateness of treatment for hypoxic-ischemic encephalopathy. These findings have been sustained for 15 months. Further investigation of broader application is warranted.

## Global Dissemination & Implementation

### S39 Building capacity for implementation science in global health: four years of experience with the University of Washington’s PhD in implementation science

#### Kenneth Sherr, Emmanuela Gakidou, Stephen Gloyd

##### Global Health, University of Washington, Seattle, WA, 98105, USA

###### **Correspondence:** Kenneth Sherr

**Background**

Limited capacity for implementation science (IS) in low and middle-income countries (LMICs) contributes to poor coverage of evidence-based intervention. Though a range of IS training options are needed to meet the diverse needs for IS practitioners, there is an acute shortage of doctoral-level trainees with adequate skills to lead IS from in-country universities, Ministries of Health, and partner institutions. To respond to the need for advanced IS skills in LMICs, the University of Washington Department of Global Health developed a doctoral-level training program to prepare future IS leaders, emphasizing students based at leading institutions conducting research, developing policy and strategy, and driving implementation in LMICs.

**Methods**

Development of the PhD program in Global Health Metrics and Implementation Science began in 2011, enrolling the first cohort in the 2012-2013 academic year. The program has two areas of emphasis – Metrics and Implementation Science. This presentation shares the experience with student recruitment and retention, curriculum and skills-based training, and dissertation research within the Implementation Science area of emphasis.

**Findings**

The PhD program has continues to demonstrate high demand for IS training at the doctoral level. There are consistently over 60 applicants per year from over 30 countries for the implementation science area of emphasis, with 5-10% of applicants admitted to the program. A total of 13 IS students enrolled in the first four cohorts, 11 (85%) from LMICs (including Kenya ^(4)^, China ^(3)^, Rwanda ^(1)^, Uganda ^(1)^, Sudan ^(1)^, Mozambique ^(1)^). Support for trainees has been primarily from research and teaching assistantships, with dedicated training funds for students from Kenya and China. Students include junior faculty in LMIC universities or research institutions (5;38%), non-governmental organizations (3;23%), the Ministry of Health (1;8%), and a number who were not based at a specific institution (4;31%). To date, one student has graduated. This presentation will also describe the curriculum pathway and dissertation topics for implementation science students.

**Implications for D&I Research**

Doctoral-level IS training is needed to develop a global workforce with skills to design, evaluate, and disseminate effective strategies to implement and scale-up evidence-based interventions. Our novel PhD program provides a model for others designing novel advanced IS training programs.

### S40 Adapting an adherence support workers intervention: engaging traditional healers as adherence partners for persons taking antiretroviral therapy in rural Mozambique

#### Carolyn Audet^1^, Jose Salato^2^, Sten Vermund^3^, Rivet Amico^4^

##### ^1^Health Policy, Vanderbilt University Medical Center, Nashville, TN, 37209, USA; ^2^Community Health, Friends in Global Health, Quelimane, 00000, Mozambique; ^3^Department of Pediatrics, Vanderbilt University Medical Center, Nashville, TN, 37203, USA; ^4^Health Behavior Health Education, University of Michigan, Ann Arbor, MI, 48109, USA

###### **Correspondence:** Carolyn Audet

**Background**

Systematic adaptation of evidence-informed interventions to increase retention in care and improve adherence to antiretroviral therapy (ART) will help disseminate innovation in rural sub-Saharan Africa. We selected and adapted an adherence support worker intervention employed in Malawi for use in rural Mozambique. Given the levels of trust and dependence previously expressed by patients for traditional medicine, we adapted the program to engage traditional healers within the allopathic health system.

**Methods**

Adaption followed a theoretically-driven approach to intervention adaption, the Assessment-Decision-Administration-Production-Topical Experts-Integration-Training-Testing model (ADAPT-ITT). Three rounds of performance-feedback based on theater presentations of the adapted intervention for stakeholders and idea generation were completed with 12 groups to develop the final model from March - July 2016. We offered healer support to 153 newly diagnosed HIV-infected patients.

**Findings**

Traditional healers, clinicians, and interested community members suggested novel strategies to tailor the Adherence Support Worker intervention, revealing a local culture of HIV-denialism, aversion to the health system and dislike of health care providers, as well as a preference for traditional treatments. Proposed changes to the intervention included modifications to the training language and topics, expanded community-based activities to support acceptability of an HIV diagnosis and to facilitate partner disclosure, and accompaniment to the health facility by healers to encourage delivery of respectful clinical care. Patients, healers, and clinicians deemed the intervention socially acceptable during focus groups. We subsequently recruited 153 newly diagnosed HIV-infected patients into the program; 146 (95%) accepted.

**Implications for D&I Research**

Systematic translation of interventions, even between regions with similar social and economic environments, is an important first step to successful program implementation. Using theater-based performances to demonstrate delivery of the intervention generated discussion about social norms, community concerns, and the merits of an acceptable strategy to improve retention and adherence to ART.

**Primary Funding Source**

National Institutes of Health - NIMH K01 award

### S41 Evaluating partners in health's mental health integration program in Burera district, Rwanda

#### Stephanie Smith^1,2^, Beatha Nyirandagijimana^3^, Hildegarde Mukasakindi^3^, Christian Rusangwa^4^, Molly Franke^5^, Giuseppe Raviola^1,5^

##### ^1^Mental Health, Partners In Health, Boston, MA, 02199, USA; ^2^Psychiatry, Brigham and Women's Hospital, Boston, MA, 02115, USA; ^3^Mental Health, Inshuti Mu Buzima, Butaro, Rwanda; ^4^Health Systems Strengthening, Inshuti Mu Buzima, Butaro, Rwanda; ^5^Department of Global Health and Social Medicine, Harvard Medical School, Boston, MA, 02115, USA

###### **Correspondence:** Stephanie Smith

**Background**

Integrating mental health care into primary care could reduce the high global burden of mental disorders. In 2012, Partners In Health and the Rwandan Ministry of Health adapted an evidence-based program of supported supervision based on task shifting for HIV/AIDS care (Monitoring and Enhanced Supervision at Health Centers, MESH), to include care of severe mental disorders within primary care settings, specifically at rural satellite health centers. The aim of this study was to evaluate implementation processes and outcomes for patients seeking care at health centers participating in the MESH mental health program in one rural district of Rwanda.

**Methods**

All qualifying adults diagnosed with a neuropsychiatric disorder at four selected health centers were enrolled in the study over a nine-month period (n = 146). Assessments of clinical and functional status were conducted at baseline, two, and six month follow-up, using a single group cohort design. An evaluation of implementation efficacy was performed using service utilization data to assess mental health service uptake at eleven participating district health centers over a six month period, and using MESH mental health supervision checklists to determine whether primary care nurses adequately assessed and treated patients receiving care at select health centers.

**Findings**

Over six months, a total of 2293 mental health visits occurred at district health centers participating in the MESH MH program. The proportion of initial mental health evaluation questions performed correctly by primary care nurses at select health centers improved from 0.35 to 0.8. There were significant improvements in patient scores on the General Health Questionnaire (25.0 [95% CI: 23.6–26.3] to 11.8 [95% CI: 10.8-12.9], p < 0.0001) and the WHO-DAS Brief (25.5 [95%CI: 23.6-27.3] to 8.0 [95% CI: 6.7-9.4], p < 0.0001).

**Implications for D&I Research**

In this study, we demonstrate that primary care nurses in resource-limited settings can provide effective mental health care to patients living with severe mental disorders using an evidence based supported supervision program. We also show that effective implementation of the MESH Mental Health program results in significant clinical and functional improvement for patients.

### S42 A multi-modal intervention to improve care of the severely ill in western Uganda: a prospective implementation study

#### Matthew Cummings^1^, Elijah Goldberg^2^, Savio Mwaka^2^, Olive Kabajaasi^2^, Adithya Cattamanchi^3^, Achilles Katamba^4^, Shevin Jacob^5^, Nathan Kenya-Mugisha^2^, J. Lucian Davis^6^

##### ^1^Medicine, New York Presbyterian - Columbia University Medical Center, New York, NY, 10032, USA; ^2^Implementation, Walimu, Kampala, Uganda; ^3^Medicine, University of California San Francisco, San Francisco, CA, 94110, USA; ^4^Medicine, Makerere College of Health Sciences, Kampala, Uganda; ^5^Medicine, University of Washington, Seattle, WA, 98104, USA; ^6^Epidemiology (Microbial Diseases), Yale School of Public Health, New Haven, CT, 06520-8034, USA

###### **Correspondence:** J. Lucian Davis

**Background**

In low-income countries with a high burden of HIV-infection, the prevalence of severe illnesses such as hemodynamic shock and severe respiratory distress is high. In such settings, optimal management of severe illness remains challenged by infrequent vital sign monitoring and lack of standardized treatment practices. We sought to determine the impact of adding a structured implementation-support intervention to a standardized clinical training program on the identification and management of severely ill patients in western Uganda.

**Methods**

We conducted a prospective cohort study of clinician practices before and after introduction of a complex, multi-modal education and training intervention using a quasi-randomized, stepped-wedge, implementation design at 4 inpatient health facilities in Kabarole District, western Uganda. Training interventions were based on World Health Organization guidelines for management of severe illness in resource-limited settings.

**Findings**

From August 2014-May 2015, 6,028 patients were enrolled; 1,698 in the pre-intervention cohort and 4,330 in the intervention cohort. In-hospital mortality was significantly higher among HIV-infected vs. HIV-negative patients (14.6% vs. 3.1%; p < 0.001). Among all patients, rates of shock and severe respiratory distress were high, identified on 15.7% and 4.7% of all patient-days, respectively. Compared to the pre-intervention cohort, patients in the intervention cohort were more likely to have ≥3 vital signs collected (42.3% vs.4.3%; p < 0.001). For patients diagnosed with shock and severe respiratory distress, those in the intervention cohort were more likely to receive intravenous fluid resuscitation (53.6% vs. 36.6%; p < 0.001) and supplemental oxygen (61.1% vs. 16.4%; p < 0.001) within 24 hours, respectively.

**Implications for D&I Research**

Standardized clinical training programs plus post-training implementatuin support can improve identification and management of severe illness in resource-limited settings. Further prospective assessment of such interventions using validated implementation frameworks is needed.

**Primary Funding Source**

Other (please specify below) - An anonymous private foundation based in the Netherlands supporting work to improve care of patients with HIV/AIDS

### S43 Context matters: adapting the Model for Understanding Success in Quality Improvement (MUSIQ) for low and middle income countries

#### Julie Reed^1^, Rohit Ramaswamy^2^, Gareth Parry^3^, Sylvia Sax^4^, Heather Kaplan^5^

##### ^1^Public Health and Primary Care, NIHR CLAHRC NWL, London, SW10 9NH, UK; ^2^Gillings School of Public Health, University of North Carolina, Chapel Hill, NC, 27599, USA; ^3^Institute for Healthcare Improvement, Boston, MA, 02138, USA; ^4^Independent Global Consultant, Heidelberg, 69118, Germany; ^5^Divisions of Neonatology, Cincinnati Children's Hospital Medical Center, Cincinnati, OH, 45229, USA

###### **Correspondence:** Rohit Ramaswamy

**Background**

The importance of incorporating local context in designing quality improvement (QI) initiatives has received increasing attention in implementation and improvement fields. The environment where change occurs has been highlighted as an explanatory factor for why some interventions work in some settings, but not in others. This observation led to the development and refinement of the Model for Understanding Success in Quality (MUSIQv2.0) to define what context is and how it interacts with implementation and improvement efforts. However, MUSIQ was developed and refined in high resource settings and the applicability of the results to low and middle income countries (LMICs) is unknown.

**Methods**

To explore the application of MUSIQ in LMICs, a dialogue collaborative process was utilised to draw on the authors’ individual experiences and understanding of context and to generate, through iterative development, a group understanding of context applied to improvement initiatives in LMICs. Authors of MUSIQv2.0, JR and HK have experience of studying context in high income countries and RR, SS and GP have experience of improvement in both settings. The process consisted of iterative discussions and refinement on how the MUSIQ findings applied to low resource settings.

**Findings**

Initial findings demonstrate that the three types of context, identified through MUSIQv2.0 for high income settings, were found to be relevant to LMIC: the care delivery where a QI intervention is introduced; the QI team conducting a QI project; and the wider context supporting general QI. The collaborative dialog revealed that while these categories are applicable for LMICs, their manifestation differed. For example, the QI team, often consisting of 6-8 personnel in HICs may have no more than one or two people in LMICs. This dialog resulted in recommendations for a simplified version of MUSIQ 2.0 applicable to LMICs which will be completed by November 2016.

**Implications for D&I Research**

This work articulates how interpretation of contextual factors affecting implementation can vary by setting, especially between HICs and LMICs. Insights from such comparisons can support design and modification of improvement initiatives and their evaluation as they are spread across a variety of countries.

### S44 Integrating evidence-based pediatric behavioral health services into primary and community settings: pragmatic strategies and lessons learned from literature review and global implementation projects

#### Keng-yen Huang^1^, Sabrina Cheng^1^, Susan Yee^1^, Kimberly Hoagwood^2^, Mary McKay^3,4^, Donna Shelley^1^, Gbenga Ogedegbe^5^, Laurie Miller Brotman^1^

##### ^1^Populaiton Health, Population Health at NYU Langone Medical Center, New York, NY, 10016, USA; ^2^Child & Adolescent Psychiatry, The Child Study Center at NYU Langone Medical Center, New York, NY, 10016, USA; ^3^Social Work, McSilver Institute for Poverty Policy and Research, New York, NY, 10013, USA ; ^4^Social Work, New York University, New York, NY, 10013, USA; ^5^Population Health and Medicine and Center for Healthful Behavior Change, New York University, New York, NY, 10016, USA

###### **Correspondence:** Keng-yen Huang

**Background**

Promoting pediatric health in low-resource and low-and-middle-income-country (LMIC) settings face numerous challenges in global health research. Children growing up in these settings tend to live in environments characterized by extreme poverty and violence. Toxic stressors combine to yield suboptimal child behavioral health. Although numerous evidence-based interventions (EBIs) are relevant for efforts to promote child behavioral health in low-resource settings, research on disseminating EBIs to these settings is extremely limited. Major challenges include: 1) lack of systematic review on pediatric behavioral health services from dissemination and implementation (D&I) perspectives; and 2) lack of general guidance for pediatric researchers on strategies to initiate D&I research. This paper addresses these gaps by focusing on two implementation objectives: 1) synthesizing pediatric D&I and behavioral health literature by identifying gaps and solutions (in multilevel contexts) that have been studied in primary and community service settings; and 2) generating a list of pragmatic guidance for pediatric researchers based on lessons learned from our global implementation projects (e.g., US, Uganda, Nepal).

**Methods**

The review of the study is guided by the Consolidated Framework for Implementation Research. A systematic review of US and global health pediatric health literature over the past 10 years has been initiated. Pragmatic strategies and research lessons have been generated based on co-authors' D&I project experiences in diverse community settings.

**Findings**

Preliminary results from the ongoing literature review identified several common barriers at the consumer, service provider, implementer, and system/policy levels (e.g., low health literacy, poor provider competency). Also, several D&I strategies have been found to be effective in addressing these implementation gaps (e.g., patient education models, policy stakeholder capacity building approaches). Guidance on conducting D&I research has been generated from lessons learned in multiple projects, such as applying effectiveness-implementation hybrid design in intervention study and characterizing inner and outer pediatric service contexts.

**Implications for D&I Research**

This study addresses important pediatric D&I research gaps by synthesizing literature. Considering the complexity of practice health research, this study also generates useful D&I guidance from multiple implementation projects to inform future pediatric behavioral health research in LMIC and US settings.

**Primary Funding Source**

National Institutes of Health - NIMH U19 MH110001-01; UBS Optimus Foundation Geneva

## Health Policy Dissemination and Implementation

### S45 Distortion of implementation techniques in health care: the case of "facilitation"

#### Roman Kislov^1^, John Humphreys^2^, Gill Harvey^3^, Paul Wilson^1^

##### ^1^Alliance Manchester Business School, University of Manchester, Manchester, M15 6 PB, UK; ^2^NIHR CLAHRC Greater Manchester, Salford Royal NHS Foundation Trust, Salford, M6 8HD, UK; ^3^School of Nursing, The University of Adelaide, Adelaide, SA 5005, Australia

###### **Correspondence:** Paul Wilson

**Background**

When applied to solving real-world problems of health care, service improvement approaches are likely to evolve over time in response to the context of their implementation. The temporal dynamics of this evolution and its underlying processes, however, remain under-researched. To address this gap, we explore the evolution of *facilitation*, an implementation approach that can be broadly defined as enabling the processes of learning in group contexts and is often deployed to mobilize research knowledge into clinical practice.

**Methods**

The prospective longitudinal case study was conducted in a five-year UK-based collaborative research programme involving a university, a tertiary-care hospital and a number of local general practices. The programme aimed to increase the identification of chronic kidney disease (CKD) and improve the management of blood pressure in CKD patients by facilitating the mobilization of existing health research in day-to-day clinical practice. A purposive sampling strategy was used, with 40 research participants drawn both from the programme 'core' team and participating general practices. 45 semi-structured interviews (30-95 minutes in duration) served as the main method of data collection and were conducted (face-to-face or by phone) in three rounds (2010-2011, 2012-2013 and 2013-2014) to enable longitudinal analysis.

**Findings**

We argue that an uncritical and uncontrolled adaptation of implementation techniques may lead to their gradual *distortion,* undermining their promise to positively affect organizational learning processes and masking the unsustainable nature of the resulting improvement outcomes captured by conventional performance measurement. We describe the following three parallel and overlapping micro-processes underpinning the gradual distortion of facilitation over time: ^(1)^ prioritization of (measurable) outcomes over the (interactive) process; ^(2)^ reduction of (multiprofessional) team engagement and ^(3)^ erosion of the facilitator role.

**Implications for D&I Research**

Our findings emphasize the lack of attention to the sustainability of change once the short-term outcomes of facilitated implementation projects have been attained, measured and reported. An exploration of new ways of maintaining context-sensitive adaptation of implementation techniques without losing their core elements could provide a useful direction for future empirical inquiry.

### S46 Measuring the cost of patient-centered medical home implementation

#### Robert Lieberthal^1,2,3^, Colleen Payton^4^, Mona Sarfaty^4^, George Valko^4^

##### ^1^Jefferson School of Population Health, Thomas Jefferson University, Philadelphia, PA, 19107, USA; ^2^Sidney Kimmel Cancer Center, Thomas Jefferson University, Philadelphia, PA, 19107, USA ^3^Department of Public Health, University of Tennessee, Knoxville, Knoxville, TN, 37996, USA; ^4^Department of Family and Community Medicine, Thomas Jefferson University, Philadelphia, PA, 19107, USA

###### **Correspondence:** Robert Lieberthal

**Background**

The 2010 Patient Protection and Affordable Care Act (ACA) and a number of state-based initiatives have significantly increased the incentives for primary care practices to become patient-centered medical homes (PCMHs). Currently, the cost for individual practices to become more patient-centered is not well recognized.

**Methods**

The objectives of this study are to develop a methodology to inventory the costly activities involved in implementing the PCMH model and apply that methodology to practices that had transformed to PCMHs. A convenience sample of eleven small and medium sized primary care practices in southeastern Pennsylvania that had previously attained NCQA PCMH recognition enrolled in our study. We assess the cost of PCMH transformation by categorizing the clinical activities required to attain PCMH recognition. We apply the economic principles of production to the primary care practice in order to develop an economic taxonomy for the costs of clinical activities.

**Findings**

In our taxonomy, the cost of each PCMH-related clinical activity can be classified as either 1) an NCQA activity category, 2) recognition as a PCMH, or 3) a change in practice culture. Cost offsets including financial incentives, pay for performance bonuses, and productivity improvements can be used to offset the cost of the PCMH model. We demonstrate the applicability of this method by applying it to medical and and nurse-led PCMH practices.

**Implications for D&I Research**

Small and medium sized practices may experience particular difficulty in separating PCMH and non-PCMH activities. Tools for planning the transition to PCMH could facilitate adoption of the model in a cost-effective manner.

**Primary Funding Source**

Agency for Healthcare Research and Quality - AHRQ grant R03-HS22630

### S47 Successful strategies for implementing patient centered care in VA medical centers

#### Rendelle Bolton^1^, Carol VanDeusen Lukas^2^, Christine Hartmann^3,4^, Nora Mueller^5^, Sally K Holmes^4,6^, Barbara Bokhour^1,4^

##### ^1^ENRM Veterans Hospital, VA HSR&D Center for Healthcare Organization and Implementation Research (CHOIR), Bedford, MA, 01730, USA; ^2^Center for Organization, Leadership & Mgmt. Rsch., Department of Veterans Affairs/Boston University-School of Public Health, Boston, MA, 02130, USA; ^3^Department of Veterans Affairs, Center for Healthcare Organization and Implementation Research (CHOIR), Bedford, MA, 01730, USA; ^4^Health Law, Policy and Management, Boston University School of Public Health, Boston, MA, 02118, USA; ^5^Center for Healthcare Organization and Implementation Research, VA HSR&D Center for Healthcare Organization and Implementation Research (CHOIR), Bedford, MA, 01730, USA; ^6^Research, VA HSR&D Center for Healthcare Organization and Implementation Research, Bedford/Boston, MA, 02130, USA

###### **Correspondence:** Rendelle Bolton

**Background**

The Veterans Health Administration (VHA) Office of Patient-Centered Care and Cultural Transformation (OPCC&CT) spearheads VHA’s effort to transform facility culture to embody patient-centered care (PCC). As part of this effort, OPCC&CT sought to identify optimal strategies used by facilities to successfully implement PCC. Drawing on prior work, this study examined the impact of key areas on PCC implementation at VA medical centers including the role of leadership, Veteran engagement, staff enculturation efforts, strategies to foster PCC innovation; staff roles and priorities; and organizational structures.

**Methods**

Semi-structured interviews were conducted with individuals leading PCC transformation at 31 geographically diverse VA medical centers, representing 18 of VA’s 21 official regions. These facilities were identified by regional PCC leaders, and were categorized by the regional leader as high, medium, or low performing with respect to perceived success with PCC implementation. Participants were asked about barriers, facilitators, and contextual factors that influenced implementation of PCC initiatives. Using a content analysis, transcripts were analyzed iteratively with both a priori and emergent codes grounded in interview data. We drew on the previously identified key areas critical for patient-centered transformation as sensitizing concepts for our analysis, and compared data across high, medium, and low performing facilities.

**Findings**

Key differences between high, medium, and low facilities were identified in the areas of leadership, Veteran engagement, staff enculturation, strategies to foster innovation, and organizational structures to support PCC. High performing facilities described receiving concrete leadership support, formally engaged Veterans in program development and implementation, and developed PCC programing from ideas generated by multiple stakeholder groups. They also developed infrastructure to support PCC programming and sustained staff enculturation efforts. Low performing facilities trained less staff in PCC principles, and had limited staff resources to champion PCC. Staff buy-in and competing priorities were challenges for all facilities.

**Implications for D&I Research**

As healthcare systems implement policies to promote patient-centered care in medical centers, attention should be given to actively engage leadership, patients, and staff at all stages of PCC implementation. Creating organizational infrastructure to support PCC is critical to the success of cultural transformation efforts.

**Primary Funding Source**

Department of Veterans Affairs - US Department of Veterans Affairs Office of Patient-Centered Care and Cultural Transformation, and QUERI program

### S48 Mapping a method for rapid dissemination and implementation: the primary care extension program

#### Sarah Ono^1,2^, Benjamin Crabtree^3^, Leah Gordon^1^, William Miller^4^, Bijal Balasubramanian^5^, Leif Solberg^6^, Deborah Cohen^1^

##### ^1^Department of Family Medicine, Oregon Health & Science University, Portland, OR, 97239, USA; ^2^VA Portland Health Care System, Department of Veterans Affairs, Portland, OR, 97239, USA; ^3^Department of Family Medicine and Community Health, Rutgers Robert Wood Johnson Medical School, New Brunswick, NJ, 08901, USA; ^4^Family Medicine, Lehigh Valley Hospital, Allentown, PA, 18105-7017, USA; ^5^Epidemiology, University of Texas Health Science Center at Houston, Houston, TX, 77030, USA; ^6^Institute for Education and Research, HealthPartners, Bloomington, MN, 55425, USA

###### **Correspondence:** Sarah Ono

**Background**

Primary Care Extension Programs (PCEP) were authorized, but not funded, through the Affordable Care Act in 2010. PCEPs are envisioned as a network of primary care practices, academic institutions, and state, federal, and community-based health extension agents that would institutionalize a resource, akin to the US Agricultural Cooperative Extension System, that would facilitate quality improvement of primary health care. In 2015, AHRQ funded EvidenceNOW so that seven regional Cooperatives would help move this extension-development forward. These Cooperatives were each tasked with covering a single state or multi-state contiguous region and each engaging 250 small primary care practices to rapidly disseminate and implement evidence related to cardiovascular disease prevention.

**Methods**

An EvidenceNOW National Evaluation was also funded by AHRQ to conduct a mixed methods evaluation of these interventions, using a range of tested and novel data collection approaches. Cross-case analyses of the infrastructure and resources across the diverse contexts of the seven Cooperatives were conducted to identify existing extension elements and variation in their design and function for regions at various stages of network development.

**Findings**

EvidenceNOW Cooperatives are using a combination of practice facilitation, expert consultation, HIT support, peer learning, and data feedback with benchmarking to support widespread dissemination and implementation of cardiovascular prevention services to practices in their regions. Cooperatives mobilized existing infrastructure (e.g., trained workforces, centralized data extraction capacity, and connections to local programs), capitalizing on partnerships to advance and explore PCEP potential. We identified variation in the PCEP models and show how these are influenced by regional resources, local politics, and unique visions for how to optimize and build networks. Our findings show that states considering developing a PCEP need a finite set of elements to support rapid dissemination and implementation.

**Implications for D&I Research**

Regional leaders who wish to develop a PCEP need to identify and engage the resources in their region in order to develop key partnerships and invest in building critical infrastructure. The goal of building PCEPs is to optimize health and health care by facilitating dissemination and implementation of evidence-based practices into primary care throughout the country, and support ongoing quality improvement on a national scale.

**Primary Funding Source**

Agency for Healthcare Research and Quality - R01: National Evaluation of EvidenceNOW Initiative

### S49 Department of Defense (DoD) practice based implementation network: an optimized framework for provision of behavioral health interventions to the Military Health System (MHS)

#### Kate McGraw^1^, Andrew Blatt^2^, Demietrice Pittman^3^

##### ^1^DHCC, Defense Centers of Excellence for Psychological Health and Traumatic Brain Injury, Silver Spring, MD, 20910, USA; ^2^DHCC/PHCC, Defense Centers of Excellence for Psychological Health & TBI, Silver Spring, MD, 20901, USA; ^3^Implementation Science Team, Defense Centers of Excellence for Psychological Health and Traumatic Brain Injury (DCoE), Silver Spring, MD, 20910, USA

###### **Correspondence:** Kate McGraw

**Background**

In 2014, the Institute of Medicine reported that two decades may pass before psychological health research findings become part of routine clinical practice. The DoD in conjunction with the Department of Veterans Affairs (VA) developed a Practice Based Implementation (PBI) Network to rapidly translate psychological health research findings into clinical practice by facilitating practice change. In 2015, 14 military treatment facility sites were engaged to pilot the implementation of outcomes monitoring of PTSD treatment, and alcohol misuse screening and by 2016 the Defense Health Agency sustained the PBI Network.

**Methods**

Based upon the Promoting Action on Research Implementations in Health Services and the VA Quality Enhancement Research Initiative frameworks, the initial pilot effort presented Evidence Based Practices (EBPs) to clinicians through trainings that respect clinic culture and context while providing continuous support and facilitation to pilot sites to try to increase provider knowledge and accountability, promote coordination and information sharing, and potentially reduces cost by testing implementation initiatives prior to broader dissemination throughout the enterprise. Simultaneously a website was developed to serve as repository of shared resources and lessons learned.

**Findings**

Strong leadership engagement was associated with increased participation and an engaged clinic staff was associated with increased site commitment to develop and implement solutions. Feedback indicated increases in usage of outcome measures and positive PTSD Check List use to monitor treatment progress as well as consistent method to track care planning and delivery. Finally, increased leadership prioritization of outcome measure use as necessary for practice changes but access and ability to provide care tended to be ongoing barriers to comprehensive utilization of EBPs.

**Implications for D&I Research**

The PBI Network sustainment project is poised to provide ongoing training and clinician informed survey research feedback from an active Implementation Science informed knowledge dissemination network within one of the largest U.S. healthcare systems. This presentation will relate the processes and science that have served as foundation for more rapid adoption of behavioral health practice changes within the U.S. military.

**Primary Funding Source**

Joint Incentive Fund (JIF)

### S50 Testing un-learning and substitution strategies to de-implement antipsychotics in nursing homes

#### Megan McCullough^1,2^, Christine Hartmann^1,2^, Helen Kales^3,4^, Dan Berlowitz^1,2^, Teresa Hudson^5,6^, Chris Gillespie^1^, Christian Helfrich^7,8^

##### ^1^Center for Healthcare Organization and Implementation Research (CHOIR), VA Health Services Research & Development, Bedford, MA, 01730, USA; ^2^Department of Health Law, Policy and Management, Boston University School of Public Health, Boston, MA, 02118, USA; ^3^Deparment of Veterans Affairs, Center for Practice Management Outcomes Research, Ann Arbor, MI, 48105, USA; ^4^Department of Psychiatry, University of Michigan, Ann Arbor, MI, 48105, USA; ^5^Psychiatric Research Institute, University of Arkansas for Medical Sciences, Little Rock, AR, 72205, USA; ^6^Department of Veterans Affairs, VA Central Arkansas Veterans Healthcare System, North Little Rock, AR, 72114, USA; ^7^Health Services Research and Development, VA Puget Sound Health Care System, Seattle, WA, 98101, USA; ^8^Department of Health Services, University of Washington, School of Public Health, Seattle, WA, 98195, USA

###### **Correspondence:** Megan McCullough

**Background**

Medical overuse (i.e., treatment that provides no benefit and/or harm) represents 10%-46% of care depending on setting and practice. Use of antipsychotic medications to manage behavioral and psychological symptoms of dementia (BPSD) in nursing homes is an example of overuse. Antipsychotics in this setting are frequently used to manage dementia patients’ BPSD. Despite limited evidence of efficacy and significant evidence of risks including mortality, 1 in 4 dementia patients in the Veterans Health Administration (VHA) Community Living Centers (CLCs—i.e., nursing homes) is prescribed antipsychotics. We developed a planned action model founded on the utility of two distinct, synergistic processes: 1) unlearning; and 2) substitution. Building on prior work, our objective is to operationalize and test unlearning and substitution strategies in the de-implementation of antipsychotic use in 8 VHA CLCs.

**Methods**

This project tests unlearning and substitution via a stepped-wedge design. Academic detailing (i.e., unlearning) promotes change in prescribing habits through awareness of the limited effectiveness and significant adverse effects of antipsychotics. The WeCareAdvisor™ is an on-line tool for use by frontline CLC staff that contains the DICE (Describe, Investigate, Create, Evaluate) Approach for assessment and management of BPSD via an ecobiopsychosocial model (i.e., substitution). The tool guides them through assessing CLC residents’ symptoms and context and prompts them with individualized ways to address BPSD and underlying causes through behavioral and environmental interventions. This project includes a mixed-methods evaluation, including an interrupted time seriesanalysis of changes in prescribing and a quantitative and qualitative evaluation of the process of testing these strategies.

**Findings**

We will provide concrete guidance on operationalizing and measuring unlearning and substitution strategies in nursing home setting. We hope to help other researchers identify the necessary conditions to determine which strategy is more or less effective, or more or less easily deployed. We will also catalogue unintended consequences, particularly related to clinician morale, and outline potential mitigators.

**Implications for D&I Research**

We propose concrete ideas on operationalizing and testing unlearning and substitution strategies. Lessons about the unintended consequences of implementing these de-implementation strategies will also add to the practical and conceptual knowledge about de-implementation

**Primary Funding Source**

Department of Veterans Affairs

### S51 A scoping review to evaluate the impact of prescription drug monitoring program implementation

#### Erin Finley^1,2^, Ashley Garcia^3^, Kristen Rosen^3^, Claudina Tami^3^, Don McGeary^3^, Mary Jo Pugh^2^, Jennifer Sharpe Potter^3^

##### ^1^School of Medicine, University of Texas Health Science Center at San Antonio, San Antonio, TX, 78229, USA; ^2^Veterans Evidence-based Research Dissemination and Implementation Center, South Texas Veterans Health Care System, San Antonio, TX, 78229, USA; ^3^Psychiatry, University of Texas Health Science Center at San Antonio, San Antonio, TX, 78229, USA

###### **Correspondence:** Erin Finley

**Background**

Prescription drug monitoring programs (PDMPs) have been implemented in 49 out of 50 states in an effort to mitigate opioid-related misuse, abuse, and mortality, yet the literature evaluating the impact of PDMP policy implementation remains limited. We conducted a scoping review to: ^(1)^ describe available evidence regarding impact of PDMPs in the United States; and ^(2)^ propose a conceptual model to inform future PDMP implementation and evaluation efforts.

**Methods**

Scoping review following Arksey and O’Malley’s (2005)’s methodology. Of the 121 articles identified from the initial PubMed database search of English-language studies published between 1/1/2000-5/31/16, eleven articles were identified as relevant based on the inclusion criteria defined a priori, specifically: peer-reviewed; presents original research; provides direct assessment of outcomes related to impact or effectiveness of PDMP implementation. We extracted data from each article following a structured template, then conducted thematic analysis to synthesize results.

**Findings**

Thematic analysis revealed studies of opioid-related outcomes associated with PDMPs typically point to a shared logic for how PDMPs are expected to function: i.e., implementation of PDMPs will increase reporting and monitoring of controlled prescriptions, leading to reduced opioid prescribing, opportunities for opioid diversion and misuse, and opioid abuse and mortality. However, extant evidence for the impact of PDMPs as an opioid risk mitigation tool remains mixed, with studies reporting evidence that both supports and contradicts their efficacy. We identified four domains of opioid-related outcomes frequently examined in original studies evaluating PDMP implementation: ^(1)^ opioid prescribing; ^(2)^ opioid diversion and supply; ^(3)^ opioid misuse; and ^(4)^ opioid-related morbidity and mortality. These domains inform a proposed evaluation framework that highlights significant gaps in empirical research across each of these domains.

**Implications for D&I Research**

There is currently no standard of best practices or guidelines regarding implementation or use of PDMPs for their intended purpose, and evidence for their impact remains mixed. We propose a conceptual model for evaluating the complexities of PDMP implementation with the goals of clarifying PDMP mechanisms of impact, identifying characteristics of PDMP implementation associated with best outcomes, and maximizing the utility of PDMP policy to reduce opioid-related public health burden.

**Primary Funding Source**

Air Force Research Laboratory FA8650-15-C-6588

### S52 Baseline clinician interviews of two de-implementation projects: four factors that might inform de-implementation strategies

#### Christian Helfrich^1,2^, Krysttel Stryczek^3^, David Au^3^, Steven Zeliadt^1,2,3^, George Sayre^2,3^, Chris Gillespie^4^

##### ^1^Health Services Research and Development, VA Puget Sound Health Care System, Seattle, WA, 98101, USA; ^2^Department of Health Services, University of Washington, School of Public Health, Seattle, WA, 98195, USA; ^3^Center of Innovation for Veteran-Centered and Value-Driven Care, Department of Veterans Affairs, Seattle, WA, 98108, USA; ^4^Center for Healthcare Organization and Implementation Research (CHOIR), Department of Veterans Affairs, Bedford, MA, 01730, USA

###### **Correspondence:** Christian Helfrich

**Background**

Medical overuse, or the provision of care that provides no benefit or where harms outweigh benefits, accounts for 10% to 46% of care, depending on the clinical practice and setting. In 2015, the VA funded a multisite program to develop evidence-based strategies to de-implement ineffective or harmful clinical practices. The goal of this program is to systematically study de-implementation in multiple clinical settings and for different clinical practices. As part of this effort, we assessed provider perceptions of two practices targeted for de-implementation: inappropriate follow-up of incidental lung nodules on computed tomography (CT) scans of the chest, and use of inhaled corticosteroids (ICS) for treating exacerbations among patients with mild-to-moderate chronic obstructive pulmonary disease (COPD).

**Methods**

We have conducted 26 baseline interviews (PCPs, Pulmonologists & Radiologists) at three VA Healthcare Systems using a semi-structured interviewer guide across two on-going quality improvement projects. Broad themes were identified based on representative interview responses and grouped under higher order headings to describe distinct aspects of participants’ experiences using content analysis.

**Findings**

We found four emergent themes across the two clinical practices. Perceived patient resistance. Even when providers say they explain risks to patients, e.g., from further CT scans, they report some patients resist doing less. Concern over patient resistance may be a significant factor in de-implementing some practices whether or not patients actually express resistance or it is merely assumed by providers. Limited primary care provider (PCP) capacity for staying abreast of current research. Given the scope of primary care practice there may be variation and clear gaps in PCPs awareness of evidence and current recommendations. Substitutions. Some providers had identified substitutes, e.g., putting a patient with COPD on tiotropium as a substitute that facilitated discontinuing ICS. Shared provider responsibility. Participants perceived multiple providers, e.g., PCPs, pulmonologists, and radiologists, to be responsible for the decision for further follow-up of incidental lung nodules. This kind diffusion of responsibility may be challenging for de-implementation of some practices.

**Implications for D&I Research**

Across two very different clinical practices and settings, we identified 4 themes that could be taken into account in designing de-implementation strategies.

**Primary Funding Source**

Department of Veterans Affairs - QUERI

### S53 How do implementation strategies impact efforts to integrate evidence-based interventions into tobacco retail policy?

#### Jennifer Leeman^1^, Allison Myers^2^, Jennifer Grant^2^, Mary Wangen^3^, Tara Queen^4^

##### ^1^School of Nursing, University of North Carolina at Chapel Hill, Chapel Hill, NC, 27599, USA; ^2^Counter Tools, Carrboro, NC, 27510, USA; ^3^Health Behavior, Gillings School of Global Public Health, Chapel Hill, NC, 27599, USA; ^4^Lineberger Comprehensive Cancer Center, University of North Carolina at Chapel Hill, Chapel Hill, NC, 27599, USA

###### **Correspondence:** Jennifer Leeman

**Background**

The US tobacco industry spends $8.7 billion annually on marketing at the point of sale (POS), a practice that promotes tobacco use. Evidence-based interventions (EBIs) are available to counter POS tobacco marketing (e.g., limiting retailer density). Integrating EBIs into policy is challenging, however, and involves leveraging and accommodating local resources, existing policy, and stakeholders (allies and adversaries) throughout an uncertain and often long policy change process. Little is known about how best to support POS EBI policy integration. The present study builds on a theory-based evaluation model. Kingdon’s multiple streams theory of policy change is applied to specify five core components of the EBI policy integration process: ^(1)^ document local problems, ^(2)^ formulate evidence-informed solutions, ^(3)^ engage strategic partners, ^(4)^ raise public awareness, and ^(5)^ persuade decision makers to enact new policy. Building on Leeman’s theory of capacity-building, the model further posits that implementation strategies affect EBI policy integration through their effects on two intermediate outcomes: team-leader self-efficacy to facilitate and team performance of the five core components of the EBI policy integration process.

**Methods**

Implementation strategies (training, tools, and technical assistance) were delivered for one year to 30 community teams in one state. Surveys were conducted at baseline and 12 months to assess impact on team leaders’ self-efficacy and teams’ progress toward enacting new policies. In-depth interviews were conducted at 6 and 12 months to assess teams’ performance and barriers encountered across the five core components of EBI policy integration. Analysis included descriptive and bivariate statistics and content analysis.

**Findings**

Following one-year’s exposure to implementation strategies, team leaders’ self-efficacy increased significantly, and a greater number of teams were making progress towards enacting policies in four of six domains. Teams’ performance of 16 activities varied, with the greatest number of activities performed within the core component “document local problem.”

**Implications for D&I Research**

Additional research is need to assess implementation strategies’ impact beyond one year. Findings can inform delivery of implementation strategies and tests of their effectiveness at promoting EBI policy integration. The study’s conceptual model and measures may also contribute to research testing implementation strategies for other EBIs that require changes to local policy.

**Primary Funding Source**

Centers for Disease Control and Prevention - Centers for Disease Control and Prevention (CDC) and the National Cancer Institute/NIH, through Cooperative Agreement Number U48 DP005017-SIP to the Center for Health Promotion and Disease Prevention at the University of North Carolina at Chapel Hill.

### S54 Framing and disseminating research information to legislators and advocates involved in cancer control policy change

#### Alexandra Morshed^1,2^, Elizabeth Dodson^2,3^, Rachel Tabak^2^, Ross C Brownson^1,2^

##### ^1^Brown School, Washington University in St. Louis, Saint Louis, MO, 63130, USA; ^2^Prevention Research Center, Washington University in St. Louis, St. Louis, MO, 63130, USA; ^3^Institute for Public Health, Washington University, St. Louis, MO, 63112, USA

###### **Correspondence:** Alexandra Morshed

**Background**

Evidence-based policy plays an important role in prevention of cancer and other chronic diseases. Dissemination strategies should be informed by the needs of actors involved in policy decision-making. This study examines the differences between state legislators and advocates in how they seek and use information and what their preferences are for how research information is framed and used.

**Methods**

We carried out a cross-sectional comparison of US advocates (n = 77) and state legislators (n = 265) working on issues related to cancer control.

**Findings**

The advocates differed significantly from the legislators on all demographic characteristics. Advocates reported seeking and using information overall more frequently than legislators, though legislators used legislative research bureaus more often (0.45 point difference, p = .004). Both legislators and advocates prioritized the presentation and timeliness of research information similarly, but reported different preferences for source of research information. Legislators emphasized information delivered to them by someone they trust (0.33 point difference, p = .004), while advocates rated objectivity (-0.26 point difference, p = .03) and relevance to constituents (-0.28 point difference, p = .004) more highly. Both groups put a similar priority on research information that supports the position they hold.

**Implications for D&I Research**

Our study provides leads for development of dissemination strategies to enhance evidence-based policymaking for cancer control that are tailored to state-level legislators and advocates. For example, as legislators prioritize having information delivered to them by someone they trust, dissemination strategies should include partnering with individuals and groups that have existing personal relationships with legislators and their staff. Also, as advocates put a higher priority on information that is unbiased and relevant, ensuring that the research evidence is generalizable to their constituencies and including local data and success stories in dissemination materials targeted at advocates are important. These and other strategies should be tested for effectiveness in future research. In addition, though a growing knowledge base exists on how to disseminate research to policymakers, few studies identify strategies for dissemination to advocates or examine the process of building stronger partnerships between research and advocacy groups. Future research efforts should examine how these key actors in the policymaking process can be more effectively engaged to promote evidence-based policymaking.

**Primary Funding Source**

National Institutes of Health - This research was funded in part by the National Cancer Institute, the National Institute of Diabetes and Digestive and Kidney Diseases, and Washington University Institute of Clinical and Translational Sciences.

### S55 Using decision analysis to understand policy-makers’ use of research and local evidence for evidence-informed policies

#### R. Chris Sheldrick^1^, Thomas Mackie^2^, Justeen Hyde^3^, Laurel Leslie^4^

##### ^1^Department of Pediatrics, Tufts Medical Center, Boston, MA, 02111, USA; ^2^Institute for Health, Health Care Policy, and Aging Research, Rutgers University, New Brunswick, NJ, 08901, USA; ^3^Institute for Community Health, Malden, MA, 02148, USA; Center for Healthcare Organization and Implementation Research (CHOIR), US Department of Veterans Affairs, Bedford, MA, 01730, USA; ^4^Institute for Clinical Research and Health Policy Studies, Tufts Medical Center, Boston, MA, 02111, USA

###### **Correspondence:** R. Chris Sheldrick

**Background**

Initiatives to promote evidence use in policymaking increasingly force state-level policy-makers to make decisions about whether and how to prioritize, implement, sustain, and/or discontinue evidence-informed policies. To provide the best possible services, policymakers must consider not only the research evidence, but also the socioeconomic and politic context, absorptive capacity of relevant entities, and stakeholder-identified needs.

**Methods**

In medicine, decision analysis offers a range of methods to support rational decision making, informing everything from cost-effectiveness analyses to shared decision making with individual patients. Using a decision analysis model of physicians’ use of diagnostic tests as a guide, we constructed a decision-tree to depict policymakers’ use of research and local evidence to inform decisions regarding the implementation of EBPs. To illustrate this approach, we draw on a case example that investigates how state-level policymakers make decisions about psychotropic medication oversight policies for children in foster care. We conducted semi-structured qualitative interviews with 72 state child welfare policymakers, with at least on respondent from each of the 50 states and DC. For analysis, we employed the five systematic and visible steps of framework analysis.

**Findings**

Qualitative results suggest policymakers often rely on both research and local evidence to inform decisions about policy options. Serving as an interpretative frame, the decision-tree model highlights several aspects of such decisions that motivate local evidence use, including uncertainty regarding the effectiveness of specific policies to local population, uncertainty regarding policy costs, and expected comparative effectiveness with respect to existing policies. Our model also highlights how the collection and interpretation of local evidence—especially early in the decision making process when relevant data are often scarce—may bias decisions about potential policy options.

**Implications for D&I Research**

Decision analysis reveals how the influence of an array of factors makes even rational decision-making dynamic, complex, and fraught with uncertainty. To understand the application of research evidence across diverse contexts, implementation science should address the actual decisions policymakers face in negotiating the complex fit between available evidence and the variety of community settings. Our model implies that not just whether but how decision-makers employ local evidence may be critical to evidence-informed decisions and deserves closer examination.

### S56 A longitudinal investigation of knowledge brokering as a mechanism for integrating research evidence into health policymaking

#### Itzhak Yanovitzky, Matthew Weber, Nicole Gesualdo, Teis Kristensen

##### Communication, Rutgers University, New Brunswick, NJ, 08901, USA

###### **Correspondence:** Itzhak Yanovitzky

**Background**

Evidence-based decision making is critical to the formulation of effective health policy and practice, but use of research evidence in the policymaking process continues to be infrequent, inconsistent, and often misinformed despite efforts to increase the availability and accessibility of research to policymakers. This study examined the potential utility of knowledge brokering as a mechanism for increasing policymakers’ use of research evidence in the context of U.S. federal policies to curb childhood obesity over the past 15 years.

**Methods**

A comprehensive set of Congressional and U.S. Government documents (transcripts of Congressional bills and hearings, floor debates, and Congressional reports) concerning federal policies to decrease childhood obesity from 2000-2014 (N = 1,041 documents) were retrieved and coded manually by the research team for variables measuring the scope, type, context, and timing of research evidence use. All excerpts containing explicit reference to research evidence were analyzed quantitatively and qualitatively using Dedoose. A two-mode network of suppliers and users of research evidence was created based on the actors (legislators, government officials, witnesses, etc.) referenced in each document and was used for tracking the flow of research evidence among actors at different phases of the policymaking process.

**Findings**

Analysis of the textual data demonstrates that conceptual use of research evidence was the most common at each phase of the policymaking process, but instrumental use (i.e., using evidence to choose among policy alternatives) was more likely when evidence was presented as authoritative and the policy proposed was relatively uncontroversial or unopposed. The social network analysis of these data show clustering of use of research evidence by policy and topic, with a distinct group of legislators serving knowledge brokering functions, including knowledge transfer, knowledge exchange, and knowledge advocacy (calling for investments in research).

**Implications for D&I Research**

There is a significant opportunity to increase use of evidence in health policymaking by identifying knowledge brokers regarding a particular issue and routinely supplying them with updated, high-quality, and relevant evidence.

**Primary Funding Source**

Use of Research Evidence Award (##185220)

## Models, Measures, and Methods

### S57 Defining and developing a pragmatic construct for implementation measurement

#### Cameo Stanick^1^, Heather Halko^2^, Caitlin Dorsey^3^, Byron Powell^4^, Bryan Weiner^5^, Cara Lewis^6^

##### ^1^Hathaway-Sycamores Child and Family Services, Pasadena, CA, 91105, USA; ^2^Department of Psychology, University of Montana, Missoula, MT, 59812, USA; ^3^Department of Psychological and Brain Sciences, Indiana University, Bloomington, IN, 47405, USA; ^4^Health Policy & Management, University of North Carolina at Chapel Hill, Chapel Hill, NC, 27599-7411, USA; ^5^Department of Global Health, University of Washington, Seattle, WA, 98195, USA; ^6^Group Health Research Institute, Seattle, WA, 98101, USA

###### **Correspondence:** Cameo Stanick

**Background**

Implementation measures largely remain a scientific phenomenon, rarely employed by stakeholders to make clinical program changes. Two likely reasons are: (1) stakeholders typically are not trained to use quantitative measures; (2) measures are typically not designed for use outside of the research context (e.g., often high participant burden, low clinical relevance). If measures are not made to be more pragmatic, stakeholders will remain limited in their ability to make implementation decisions and the gap between implementation science and practice will grow. Glasgow and Riley articulated a definition for the pragmatic construct, indicating that pragmatic measures are (a) important to stakeholders, (b) of low burden for respondents and staff, (c) ‘actionable,’ and (d) sensitive to change. This definition was not informed by stakeholders, nor did it systematically integrate previous literature. Although there is clear face validity to their dimensions, it is possible that key dimensions were overlooked and/or that stakeholders may prioritize dimensions differently than scientists. The present study aimed to conduct: (1) a systematic literature review and (2) stakeholder interviews to reveal pragmatic measures dimensions.

**Methods**

PsycINFO and PubMed were the databases used to identify dimensions of the pragmatic construct. Simultaneously, an international stakeholder panel was interviewed to obtain their perspectives of pragmatic measures.

**Findings**

Combined results from the literature review and stakeholder interviews revealed a final list of 47 short statements (e.g., low cost, brief), which will allow for the development of a rigorous, stakeholder-driven conceptualization of the pragmatic measures construct and will aid in measure development to support implementation practice.

**Implications for D&I Research**

Combined results from the literature review and stakeholder interviews revealed a final list of 47 short statements (e.g., low cost, brief), which will allow for the development of a rigorous, stakeholder-driven conceptualization of the pragmatic measures construct and will aid in measure development to support implementation practice.

**Primary Funding Source**

National Institutes of Health - Acknowledgements: Research reported in this publication was supported by the National Institute of Mental Health of the National Institutes of Health under Award Number R01MH106510

### S58 Stakeholders’ perceptions of criteria for pragmatic measurement in implementation: a concept mapping approach

#### Byron Powell^1^, Bryan Weiner^2^, Cameo Stanick^3^, Heather Halko^4^, Caitlin Dorsey^5^, Cara Lewis^6^

##### ^1^Health Policy & Management, University of North Carolina at Chapel Hill, Chapel Hill, NC, 27599-7411, USA; ^2^Department of Global Health, University of Washington, Seattle, WA, 98195, USA; ^3^Hathaway-Sycamores Child and Family Services, Pasadena, CA, 91105, USA; ^4^Department of Psychology, University of Montana, Missoula, MT, 59812, USA; ^5^Department of Psychological and Brain Sciences, Indiana University, Bloomington, IN, 47405, USA; ^6^Group Health Research Institute, Seattle, WA, 98101, USA

###### **Correspondence:** Byron Powell

**Background**

There is a need for valid and reliable measures that can aid in prospectively assessing barriers and facilitators to implementing evidence-based practices; developing, selecting, and tailoring implementation strategies; and evaluating implementation outcomes. However, practitioners are unlikely to use these measures if they are not pragmatic (i.e., relevant and feasible for use in real-world settings); thus, there is a need to develop stakeholder-informed criteria by which to assess the extent to which measures are pragmatic. A previous study generated 47 criteria for pragmatic measures (e.g., easy to interpret, low cost, uses accessible language) through a structured literature review and semi-structured interviews with implementation stakeholders. The current study engaged stakeholders with expertise in implementation practice in order to 1) generate conceptually distinct clusters of criteria for pragmatic measures, and 2) to assess stakeholders’ perceptions of each criterion’s clarity and importance.

**Methods**

Twenty-four stakeholders with expertise in implementation practice were engaged in a concept mapping process, which involved structured sorting and rating tasks intended to organize the initial list of 47 criteria into conceptually distinct categories and to derive ratings of their clarity and importance. Data collection and analysis were completed using Concept Systems Global Max©, and involved the use of multidimensional scaling, hierarchical cluster analysis, and descriptive statistics

**Findings**

The 47 criteria were meaningfully grouped into four distinct categories: 1) *useful* (e.g., “able to inform decision making”), 2) *compatible* (e.g., “one that is the output of routine activities”), 3) *easy* (e.g., “brief”), and 4) *acceptable* (e.g., “offers relative advantage over existing methods”). Average ratings of clarity and importance at the category and individual criteria level will be presented.

**Implications for D&I Research**

This study illustrates how concept mapping can be leveraged to obtain stakeholder consensus, and advances implementation science by providing criteria that can be used to identify pragmatic measures. Next steps will include a Delphi process to develop consensus on the most important criteria, after which we will develop quantifiable pragmatic rating criteria that can be used to assess measures.

**Primary Funding Source**

National Institutes of Health - Acknowledgements: Research reported in this publication was supported by the National Institute of Mental Health of the National Institutes of Health under Award Number R01MH106510

### S59 Psychometric assessment of three newly developed implementation outcome measures

#### Bryan Weiner^1^, Caitlin Dorsey^2^, Cameo Stanick^3^, Heather Halko^4^, Byron Powell^5^, Cara Lewis^6^

##### ^1^Department of Global Health, University of Washington, Seattle, WA, 98195, USA; ^2^Department of Psychological and Brain Sciences, Indiana University, Bloomington, IN, 47405, USA; ^3^Hathaway-Sycamores Child and Family Services, Pasadena, CA, 91105, USA; ^4^Department of Psychology, University of Montana, Missoula, MT, 59812, USA; ^5^Health Policy & Management, University of North Carolina at Chapel Hill, Chapel Hill, NC, 27599-7411, USA; ^6^Group Health Research Institute, Seattle, WA, 98101, USA

###### **Correspondence:** Bryan Weiner

**Background**

Implementation outcome measures are essential for monitoring and evaluating the success of implementation efforts and comparing the effectiveness of alternative implementation strategies. Yet available measures lack conceptual clarity and have questionable reliability and validity. We systematically developed and psychometrically assessed three new measures of implementation outcomes: acceptability, appropriateness, and feasibility.

**Methods**

Following domain delineation and item generation, we assessed substantive and discriminant content validity by asking 36 implementation scientists and 27 mental health professionals to assign 31 items to the 3 constructs and rate their confidence in their assignments. We used the Wilcoxon one-sample signed rank test to determine whether items represent their intended constructs more so than the other constructs. We used exploratory and confirmatory factor analysis (EFA and CFA) and Cronbach ɑ to assess the validity of our conceptual model. To assess structural validity, reliability, and known-groups validity, we asked 321 mental health counselors to read one of six randomly assigned vignettes depicting a therapist contemplating adopting an evidence-based practice (EBP). Participants used 15 items to rate the therapist’s perceptions of the acceptability, appropriateness, and feasibility of adopting the EBP. Structural validity was assessed using CFA. Reliability was assessed using Cronbach ɑ. Known-groups validity was assessed using analysis of variance (ANOVA).

**Findings**

Median weighted assignments for all but five items were significantly greater than zero, indicating that participants judged the items to reflect to a significantly greater degree the constructs they were intended to measure than the other constructs. A trimmed CFA with 5 items per construct exhibited good model fit, as evidenced by CFI = 0.98 and RMSEA = 0.08 [CI, 0.04-0.11], with factor loadings between .79 and .94. The ɑ’s for the trimmed scales were between .87-.89. Structural validity and reliability data has been collected and is presently being analyzed.

**Implications for D&I Research**

The three newly developed measures demonstrate promising psychometric properties. They are also brief. In planned work, we will assess their predictive validity.

**Primary Funding Source**

National Institutes of Health - Acknowledgements: Research reported in this publication was supported by the National Institute of Mental Health of the National Institutes of Health under Award Number R01MH106510

### S60 Strategies for assessing fidelity to evidence-based interventions: a comparison of feasibility, accuracy, and associations with clinical outcomes

#### Shannon Wiltsey Stirman^1,2^, Patricia Carreno^2^, Kera Mallard^2^, Tasoula Masina^3^, Candice Monson^3^

##### ^1^Stanford University, Menlo Park, CA, 94025, USA; ^2^Dissemination and Training Division, National Center for PTSD, Menlo Park, CA, 94025, USA; ^3^Department of Psychology, Ryerson University, Toronto, ON, M4C1B5, Canada

###### **Correspondence:** Shannon Wiltsey Stirman

**Background**

Fidelity is a key implementation outcome, but measurement is challenging due to time and costs associated with observation-based fidelity assessments. Potentially scalable forms of fidelity assessment, such as self-report or interview data, may be less accurate due to a variety of response biases, and clinical notes may be subject to similar biases. Work samples such as clinical notes or “products” generated during the intervention are promising alternatives for some interventions. However, the relative feasibility and predictive validity of these approaches is unknown. This study compared self-reported, interviewer, assessed, observer-rated and work-sample rated fidelity in a sample of clinicians delivering an evidence-based psychotherapy for PTSD.

**Methods**

Monthly fidelity assessment in the form of self-report and adherence checklists embedded in clinical notes, ratings of clinical worksheets, interview-reported fidelity, and observer-rated fidelity were assessed in a sample of 45 clinicians and 90 clients. Correlations between these forms of fidelity assessment were examined. Response rates, clinician burden (time) and time required for ratings were compared for the different modes of data collection were assessed and compared.

**Findings**

Preliminary data indicates that self-reported adherence was moderate, (m = 2.28/4; sd = .71; and observer rater adherence was high (m = .89/1; sd = .17). Observer and self-reported adherence were correlated (r = .48, p = .04). Compliance rates were highest for provision of audio recordings and self-report monthly surveys, and lower for clinical notes, perhaps because certification was contingent on provision of audio recordings and direct access to clinical records was not provided. Clinician burden for providing interviews, self-report and worksheet were perceived to be higher than audio recordings, although none were perceived to be significant burdens. Rater time was most intensive for observer ratings (60 minutes per session), followed by interview (10 minutes for relevant portions) and worksheets (5-7 minutes). Associations between fidelity scores and clinical outcomes will be examined (all data are collected).

**Implications for D&I Research**

Lower-burden forms of measurement strategies may be feasible and valid. Timing fidelity ratings to avoid temporal confounds with symptom change is necessary when examining associations between fidelity and outcomes. Certain types of fidelity assessment may be more feasible and appropriate for these analyses. Strategies for adequate data capture are discussed.

**Primary Funding Source**

National Institutes of Health - NIMH R01 MH 106506; Canadian Institute of Health Research

### S61 An investigation of the impact of accuracy and frequency of self-report on observed fidelity

#### Taren Swindle^1^, Geoffrey Curran^2^, Zachary Patterson^1^, Leanne Whiteside-Mansell^1^

##### ^1^Family and Preventive Medicine, University of Arkansas for Medical Sciences, Little Rock, AR, 72205, USA; ^2^Department of Pharmacy Practice, University of Arkansas for Medical Sciences, Little Rock, AR, 72205, USA

###### **Correspondence:** Taren Swindle

**Background**

Fidelity data is used to inform strategies designed to improve uptake of evidence-based practices (EBPs). Fidelity can be assessed through direct observation or using self-report. Direct observation methods are perceived as more reliable but are more expensive. Self-report methods require less resources and may contribute to continued improvements in use of EBPs. The purpose of this study was to (a) test the convergence between direct and indirect measures of fidelity to a nutrition curriculum among early educators (N = 34) and (b) examine the relationship between accuracy and frequency of self-report and changes in observed fidelity.

**Methods**

Observational and self-report fidelity measures were developed to assess adoption of EBPs. Monthly, for 8 months, trained observers rated a WISE lesson and recorded the use of EBPs using a 1 (Not at All) to 4 (Very Much) scale. Educators submitted self-report fidelity assessing practices, also on a 4-point scale. Observation and self-report fidelity scores were created by averaging across corresponding items. Then, an average across months was computed. Correlations were positive (indicating convergence) for two practices, role modeling (*r* = .43, *p* = .02) and use of the mascot (*r* = .50, *p* = .005). Self-report and observed ratings of hands on exposure were negatively correlated (*r* = -.39, *p* = .04). A self-report accuracy score was created for each component by subtracting the self-report school-year average from the observed school-year average (i.e., negative value = educator overestimated fidelity). Finally, we conducted linear regression analyses predicting residualized change in fidelity across the school year with accuracy, number of submitted self-report forms, race, and years of experience as predictors.

**Findings**

Frequency of self-assessment of fidelity did not predict improvement in observed fidelity. However, accuracy of self-report was a significant predictor of improvement in observed fidelity for two core components, role modeling [*β* = .89, t(22) = 4.78, p < .001] and hands on exposure [*β* = .66, t(22) = 2.82, p = .02].

**Implications for D&I Research**

Overall, results suggest that some EBPs may be more accurately self-assessed than others. Further, accurate self-reports can contribute to improved fidelity over time.

**Primary Funding Source**

National Institutes of Health - NIMH R01 106506; Canadian Institute of Health Research

### S62 A pragmatic fidelity measurement strategy for integration into community practice settings

#### Rochelle Hanson^1^, Benjamin Saunders^2^, Sonja Schoenwald^1^, Angela Moreland^2^

##### ^1^Psychiatry & Behavioral Sciences, Medical University of South Carolina, Charleston, SC, 29425, USA; ^2^Psychiatry & Behavioral Sciences, National Crime Victims Research & Treatment Center, Charleston, SC, 29425, USA

###### **Correspondence:** Rochelle Hanson

**Background**

Several methods have been developed to measure fidelity to evidence-based treatments (EBTs), with an extensive research base supporting observational coding systems as the ‘gold standard,’ largely due to their ability to provide objective assessments of therapist behavior. Unfortunately, observational coding is labor and cost intensive, curtailing its feasibility and integration into community practice settings. Alternatively, therapist self-reports are potentially more feasible, but rarely evaluated psychometrically. While adherence is a frequently measured element of fidelity, few studies target treatment *competence,* the skill with which providers deliver elements of an EBT, likely due to significant challenges in defining and measuring this construct. This study examined therapist-reported competence to Trauma-Focused Cognitive Behavioral Therapy (TF-CBT), a components-based EBT targeting trauma-related behavioral health symptoms in youth.

**Methods**

Data were analyzed from a recently completed, statewide initiative that involved *n* = 516 clinical providers from mental health, child welfare, and juvenile justice service systems, participating in one of 11 learning collaboratives focused on TF-CBT. To complete training, therapists were required to complete TF-CBT with a minimum of 2 cases (*M* = 4.58; *SD* = 2.34; *N =* 2,361). Therapists reported weekly whether they had seen one of their training cases and if so, which of the 11 TF-CBT components they had delivered and their perceived skill and competence in delivering them (rated on a 5 point scale from ‘Less than adequate skill’ to ‘Expert skill’).

**Findings**

Rasch measurement models were performed, using the weekly therapist reports, with results indicating variability in self-reported competence across components (e.g., lowest ratings on exposure-based components; highest on psychoeducation, parenting skills and relaxation). Growth models performed for each of the 11 TF-CBT components indicated that 6 components increased significantly over time. Finally, mixed-effects regression models for pre- to post-treatment change on child-reported post-traumatic stress (PTS) symptoms indicated that those who received services from therapists with higher self-rated competence on some of the components had greater improvements in PTS symptoms pre-to-post treatment.

**Implications for D&I Research**

Findings suggest therapist-reported competence may offer a valid, feasible and pragmatic fidelity measurement strategy that can be integrated and sustained in community practice settings. Limitations and future directions will be discussed.

**Primary Funding Source**

National Institutes of Health - NIMH 1R34MH104470-01

### S63 Criteria for selecting implementation frameworks and theories among implementation researchers and practitioners

#### Sarah Birken^1^, Byron Powell^1^, Justin Presseau^2,3,4^

##### ^1^Health Policy & Management, University of North Carolina at Chapel Hill, Gillings School of Global Public Health, Chapel Hill, NC, 27599-7411, USA; ^2^Centre for Practice-Changing Research, Ottawa Hospital Research Institute, Ottawa, ON, K1H 8 L6, Canada; ^3^Institute of Health & Society, Newcastle University, Newcastle, NE24AX, UK; ^4^School of Epidemiology, Public Health, and Preventive Medicine, University of Ottawa, Ottawa, ON, K1H 8 L6, Canada

###### **Correspondence:** Sarah Birken

**Background**

Conceptual frameworks and theories can guide the selection and examination of implementation determinants, processes, and outcomes. However, there is a lack of guidance for selecting an appropriate framework or theory amongst the increasing number in the field. These challenges may contribute to the default use, underuse, or misuse of frameworks and theories. The objective of this study was to understand the criteria that researchers and practitioners use to select implementation frameworks and theories.

**Methods**

We triangulated a narrative review of the literature and expert review to identify a preliminary set of criteria for selecting appropriate frameworks and theories (e.g., analytic level, disciplinary approval, outcome of interest). We then conducted a self-administered web-based survey of implementation researchers and practitioners regarding their use of these and other criteria for selecting appropriate frameworks and theories. Potential respondents were recruited at the 2015 Annual Dissemination and Implementation Conference and via several listservs (e.g., Nordic Implementation Network; European Implementation Collaborative; Implementation Network).

**Findings**

143 implementation researchers and practitioners in 12 countries responded to the survey. They reported using a wide range of the criteria that we identified a priori (Table 2), and also identified an additional 49 criteria. While many of the respondents reported using criteria that reflects a pragmatic approach to selecting frameworks and theories (e.g., analytic level, outcome of interest, empirical support), many also endorsed criteria that suggest a lack of clarity about how to select frameworks and theories for their work (e.g., familiarity; habit; mentor or funder expectations).

**Implications for D&I Research**

Implementation researchers and practitioners use an unwieldy number of criteria for selecting frameworks and theories, and the criteria are more idiosyncratic than systematic. Implementation researchers and practitioners may benefit from a tool that guides framework and theory selection. However, the optimal content and format of such a tool is unclear. Future research should develop a stakeholder-informed, pragmatic tool to guide framework and theory selection.

**Table 2:** Number of criteria respondents used to select models (n = 143)

Criteria used	Frequency
<=5	30
6-10	79
> = 10	50

### S64 Readiness in context: a framework and literature synthesis comparing operationalization and measurement of organizational readiness and context

#### Isomi Miake-Lye^1,2^, David Ganz^3,4^, Brian Mittman^3,5^, Deborah Delevan^3^, Erin Finley^6,7^

##### ^1^QUERI, Department of Veterans Affairs, Greater Los Angeles Healthcare System, Los Angeles, CA, 90073, USA; ^2^Health Policy and Management, UCLA School of Public Health, Los Angeles, CA, 90024, USA; ^3^Care Coordination QUERI, VA Greater Los Angeles Healthcare System, Los Angeles, CA, 90073, USA; ^4^David Geffen School of Medicine, University of California, Los Angeles, Los Angeles, CA, 90024, USA; ^5^Research and Evaluation, Kaiser Permanente Southern California, Pasadena, CA, 91101, USA ^6^HSR&D, South Texas Veterans Health Care System, San Antonio, TX, 78229, USA ; ^7^Hospital Medicine & Psychiatry, University of Texas Health Science Center at San Antonio, San Antonio, TX, 78207, USA

###### **Correspondence:** Isomi Miake-Lye

**Background**

Despite theoretical overlap between concepts of context and organizational readiness in practice implementation, prior work has primarily addressed these as distinct constructs with unclear boundaries. To assist implementers, we sought to clarify this conceptual muddiness by describing the overlap between domains and measures used for organizational readiness and context in healthcare.

**Methods**

We identified four key publications (CFIR, Damschroder 2009; Patient Safety Culture, Taylor 2011; MUSIQ, Kaplan 2012; Evidence into Practice, McCormack 2002) describing elements of context relevant to healthcare. Synthesizing from these publications, we identified five domains central to context in healthcare: innovation characteristics; internal context; external environment; individuals involved; and implementation/ improvement strategy or process. We then mapped items from these tools to the context domains identified. Following this, we mapped items from readiness assessments identified by a systematic review (Gagnon 2014) to the same five context domains, in order to (a) assess the degree of concept overlap and (b) populate a list of context and readiness items for use in future projects. All mapping was conducted via independent coding followed by discussion to consensus where discrepancies emerged.

**Findings**

We found that items from both context and readiness measures could be categorized effectively using context domains. Of the 26 tools from the organizational readiness systematic review, we included the five measures (308 items) that were broadly relevant to healthcare settings and were both valid and reliable. When mapped to the five context domains, 26 items assessed innovation characteristics (8.4%), 195 were internal context (63.3%), 4 were external environment (1.3%), 44 were individuals involved (14.3%), 34 were implementation/ improvement strategy or process (11.0%), and 5 were not relevant to readiness or context (1.6%).

**Implications for D&I Research**

Our process suggests that readiness may well be nested within context domains, with nearly two-thirds of readiness measures included in one domain: internal context. The nesting of readiness within context raises the question of whether other constructs, such as adaptive reserve, may be similarly nested. In addition, given the relative paucity of readiness items in certain context domains, a broader pool of context measures might be useful in readiness assessment beyond those currently found in readiness instruments.

**Primary Funding Source**

Department of Veterans Affairs - QUE 15-276 Improving Patient-Centered Care Coordination for High-Risk Veterans in PACT (VA Care Coordination QUERI)

### S65 Three key analytic processes that emerged while using the Consolidated Framework for Implementation Research (CFIR) to evaluate broad-scale system change

#### Jennifer N Hill^1^, Sara Locatelli^1^, Barbara Bokhour^2,3^, Gemmae Fix^2,3^, Jeffrey Solomon^4^, Nora Mueller^5^, Sherri L. Lavela^1,6,7^

##### ^1^Center of Innovation for Complex Chronic Healthcare (CINCCH), Department of Veterans Affairs, Hines, IL, 60141, USA; ^2^ENRM Veterans Hospital, VA HSR&D Center for Healthcare Organization and Implementation Research (CHOIR), Bedford, MA, 01730, USA; ^3^Health Law Policy and Management, Boston University School of Public Health, Boston, MA, 02215, USA; ^4^Evaluating Patient-Centered Care (EPCC), VA HSR&D Center for Healthcare Organization and Implementation Research (CHOIR), Bedford, MA, 01730, USA; ^5^Center for Healthcare Organization and Implementation Research, VA HSR&D Center for Healthcare Organization and Implementation Research (CHOIR), Bedford, MA, 01730, USA; ^6^Department of Physical Medicine and Rehabilitation, Feinberg School of Medicine, Northwestern University, Chicago, IL, 60611, USA; ^7^Department of Preventive Medicine and Center for Healthcare Studies, Institute for Public Health and Medicine (IPHAM), Northwestern University, Chicago, IL, 60611, USA

###### **Correspondence:** Jennifer N Hill

**Background**

Implementation studies seek to understand factors influencing a program and their underlying relationships. A theoretically-driven approach to evaluation offers comprehensive, context-specific results with actionable recommendations for programmatic leaders. The Consolidated Framework for Implementation Research (CFIR) provides an organized, flexible structure and a common language to facilitate knowledge-building and generalizability of findings across implementation science studies. Patient-Centered Care (PCC) is personalized health care that considers a patient’s circumstances and goals. While the Department of Veterans Affairs (VA) is working towards implementing PCC throughout its healthcare system, comprised of many interventions with the long-term goal of cultural transformation, little is known about the factors influencing its implementation. We discuss three key analytic processes that emerged while using CFIR to evaluate a broad-scale system change and demonstrate how use of CFIR resulted in methodologically-sound, comprehensive, rapid, and actionable results to key leadership for use in future efforts.

**Methods**

A qualitative study design, guided by CFIR, was used to examine PCC implementation. Semi-structured interviews were conducted with senior management, middle-management, and front-line employees (n = 107) four VA Medical Centers. An interview guide was developed using CFIR and a mixed deductive-inductive approach was used for coding. CFIR constructs provided the primary deductive coding structure and thematic coding was used to inductively capture themes.

**Findings**

Application of CFIR to this broad-scale evaluation demonstrated important insights including: ^(1)^ the need for adapted definitions for CFIR constructs (especially due to new application to broad-scale change), ^(2)^ the value in using a mixed deductive-inductive approach with thematic coding to capture emergent themes not encompassed by CFIR, and 3) the advantages offered by its use for expedited analysis and synthesis for rapid delivery of findings to operational partners.

**Implications for D&I Research**

This work is among the first to describe use of CFIR to guide the evaluation of a broad-scale transformation, as opposed to discrete interventions. This evaluation demonstrates the flexibility offered by CFIR while offering important insights that should be considered in future evaluations using CFIR. Researchers should consider development of study-specific definitions of the constructs, methods that capture thematic ideas not covered in CFIR, and synthesis of findings to provide meaningful, actionable recommendations.

**Primary Funding Source**

Department of Veterans Affairs - Department of Veterans Affairs Office of Patient-Centered Care & Cultural Transformation and the VA Health Services Research & Development Quality Enhancement Research Initiative (PCE 13-001, PI: Bokhour; PCE 13-002, PI: LaVela).

### S66 Organizational readiness for implementing a new program, policy or practice: measurement and results

#### Victoria Scott^1^, Jonathan Scaccia^2^, Kassy Alia^3^, Brittany Skiles^3^, Abraham Wandersman^3^

##### ^1^Psychology, University of North Carolina Charlotte, Charlotte, NC, 28223, USA; ^2^Institute for Healthcare Improvement, Institute for Healthcare Improvement, Reading, PA, 19606, USA; ^3^Psychology, University of South Carolina, Columbia, SC, 29208, USA

###### **Correspondence:** Jonathan Scaccia

**Background**

Making large-scale gains at the population level requires tools, measures, and methods that can be used across diverse settings. Organizational readiness refers to the extent to which an organization is both *willing* and *able* to implement an innovation (program, policy, or practice that is new to its setting). Readiness is a dynamic concept that changes with shifts in the implementation setting. The ability to measure and monitor readiness for an innovation can yield rapid-cycle insights into implementation barriers and facilitators, and increase readiness for implementation. We will describe an implementation science heuristic for organizational readiness involving the (a) motivation to implement an innovation, (b) general capacities of an organization, and (c) innovation-specific capacities needed for a particular innovation; or R = MC^2^ (Readiness = Motivation x General Capacity x Innovation-Specific Capacity).

**Methods**

The R = MC^2^ implementation science approach for organizational readiness is based on an extensive organizational readiness literature. It was operationalized into an assessment tool using an inductive process for item generation and administered longitudinally to 39 communities across the U.S. as part of a national community health improvement initiative (Spreading Community Accelerators through Learning and Evaluation) project led by the Institute for Healthcare Improvement and funded by the Robert Wood Johnson Foundation. Readiness data was collected longitudinally over two years.

**Findings**

The reliability statistics (Cronbach alpha’s) for the subcomponents of the readiness assessment ranged from .73 to .95. Readiness scores were found to be significantly higher in communities received the full SCALE support model (i.e. coaching, webinars, trainings, peer groups) when compared to newly enrolled (Pathway-to-Pacesetter) communities (Hedges’ g ranged from 0.21 (Ability to pilot) to 1.56 (Implementation Climate). This session will describe how data was used to inform all stakeholders about changes in readiness within and across communities throughout the SCALE initiative.

**Implications for D&I Research**

The R = MC^2^ implementation science heuristic and questionnaire is a practically useful approach and tool for measuring and monitoring readiness for implementing a new innovation. This approach appears able to discriminate between different levels of readiness. Consequently, individualized community readiness “profiles” can be generated from the readiness data and used to inform targeted coaching in health improvement strategies.

**Primary Funding Source**

The Robert Wood Johnson Foundation

### S67 Ten years of evolving methods in implementation research

#### Paul Wilson

##### Alliance Manchester Business School, University of Manchester, Manchester, M15 6 PB, UK

**Background**

Enhancing the routine uptake of research findings remains strategically important for efforts to improve the organisation, delivery and quality of healthcare. Implementation research is the scientific study of methods to promote the systematic uptake of evidence-based interventions into practice and policy and hence improve health. It includes the study of influences on professional, patient and organisational behaviour in healthcare, community or population contexts. As a field, Implementation Science is inherently interdisciplinary and embraces a broad range of methodological approaches and research paradigms.

**Methods**

Drawing on 10 years of journal contributions, the Editors of Implementation Science will reflect on the current state of implementation science as a field of enquiry.

**Findings**

This will include reflection on: the development and evaluation of increasingly complex interventions; the increased focus on the pursuit of ‘good enough evidence’ and with it the emergence of adaptive, hybrid and stepped wedge designs as researchers seek to produce timely and appropriate evidence; and the challenges of adequately studying mechanisms of action, identifying contextual factors that moderate their effects and providing meaningful insights into process of implementation.

**Implications for D&I Research**

The panel will reflect on these issues and consider the need for methodological development in areas of increasing interest including the de-implementation of practices of low or no clinical benefit, the need for efficient designs to evaluate implementation in health systems and global public health, and the cost effectiveness of implementation efforts generally.

### S68 Theory and frameworks in implementation research: current and future challenges

#### Anne Sales^1,2^

##### ^1^ Learning Health Sciences, University of Michigan, Ann Arbor, MI, 48109-5403, USA; ^2^ Center for Clinical Management Research, VA Ann Arbor Healthcare System, Ann Arbor, MI, 48109, USA

###### **Correspondence:** Anne Sales

**Background**

Historically, poor theoretical underpinning has been a feature of implementation research with researchers rarely providing clear rationales for the choice of interventions deployed in studies. Over the last decade, *Implementation Science* has led calls for the greater use of theory to advance the science of implementation research, and an increasing number of research papers have used theoretically derived frameworks to support robust implementation intervention development. Use of theory can improve intervention design and description, help understanding of likely mechanisms of action and the influence context, as well as providing an explicit generalizable framework for analysis to explain how and why implementation succeeds or fails.

**Methods**

Drawing on 10 years of journal contributions, the Editors of *Implementation Science* will reflect on the growth of theories and frameworks relevant to implementation, and explore both current and future challenges surrounding their use.

**Findings**

Specifically we will discuss the need for continued comparative empirical testing of theories either alone or in combination, the need to balance theoretical fidelity with the questions to be addressed as well as the challenges of how, when and to what end theory alone or in combination, should be applied.

**Implications for D&I Research**

The Editor in Chief will lead discussion on the methodological developments of the last decade and discuss how *Implementation Science* can continue to play an important role in advancing use of theory in implementation research

## Precision Medicine

### S69 The current state of implementation science in genomic medicine: opportunities for improvement

#### Megan Roberts^1^, Amy Kennedy^1^, David Chambers^1^, Muin J. Khoury^2^

##### ^1^Division of Cancer Control and Population Sciences, National Cancer Institute, Bethesda, MD, 20850, USA; ^2^Office of Public Health Genomics, Centers for Disease Control and Prevention, Atlanta, GA, 30333, USA

###### **Correspondence:** Megan Roberts

**Background**

Increasingly, genomic information is being applied to disease prevention, diagnosis, and management. However, the speed of translation of evidence to improved health has been relatively slow compared to the speed of discovery. Implementation science can address the challenge of integrating genomics into real-world practice settings. The objective of this literature review is to identify trends and gaps in the field of implementation science in genomic medicine.

**Methods**

We conducted a literature review using the Centers for Disease Control and Prevention’s Public Health Genomics Knowledge Base to identify the literature in 2014 in the field of implementation science in genomic medicine. We selected original research articles based on specific inclusion criteria. We abstracted information about study design, genomic medicine and implementation outcomes. Trends and gaps in the literature were described.

**Findings**

Our final review included 283 articles, the majority of which described uptake (35.7%, n = 101) and preferences around genomic technologies (36.4%, n = 103). Most of the research was in the oncology setting (35%, n = 99). Key study design elements, such as racial/ethnic composition of study populations, were widely underreported in studies. Few studies incorporated implementation science theoretical frameworks, sustainability measures, or capacity building.

**Implications for D&I Research**

While genomic discovery provides the potential for population health benefit, the current knowledge base around implementation is limited. Current evidence gaps demonstrate a need to apply implementation science principles to genomic medicine in order to deliver on the promise of precision medicine.

**Primary Funding Source**

National Institutes of Health - Intramural National Cancer Institute funds

### S70 Challenges and strategies for implementing genomic services in diverse settings: experiences from the Implementing Genomics in Practice (IGNITE) Network

#### Nina Sperber^1,2^, Lori Orlando^3^, Janet Carpenter^4^, Larisa Cavallari^5^, Joshua Denny^6^, Amanda Elsey^5^, Fern Fitzhenry^6^, Yue Guan^7^, Carol Horowitz^8^, Julie Johnson^5^, Ebony Madden^9^, Toni Pollin^7^, Victoria Pratt^10^, Tejinder Rakhra-Burris^11^, Marc Rosenman^12^, Corrine Voils^2,13^, Kristin Weitzel^5^, Ryanne Wu^2^, Laura Damschroder^14^

##### ^1^Durham VA Medical Center, Health Services Research and Development, Durham, NC, 27701, USA; ^2^Department of Medicine, Duke University, Durham, NC, 27705, USA; ^3^Duke University, Durham, NC, 27708, USA; ^4^School of Nursing, Indiana University, Indianapolis, IN, 46202, USA; ^5^College of Pharmacy, University of Florida, Gainesville, FL, 32610-0486, USA; ^6^Department of Biomedical Informatics, Vanderbilt University, Nashville, TN, 37203, USA; ^7^School of Medicine, University of Maryland, Baltimore, MD, 21201, USA; ^8^Population Health Science and Policy, Icahn School of Medicine at Mount Sinai, New York, NY, 10029, USA; ^9^National Human Genome Research Institute, NIH, Bethesda, MD, 20852, USA; ^10^Department of Medical and Molecular Genetics, Indiana University, Indianapolis, IN, 46202, USA; ^11^Center for Applied Genomics and Precision Medicine, Duke University, Durham, NC, 27708, USA; ^12^School of Medicine, Indiana University, Indianapolis, IN, 46202, USA; ^13^Health Services Research & Development, Durham VAMC, Durham, NC, 27705, USA; ^14^Center for Clinical Management Research, Veterans Affairs, Ann Arbor, MI, 48113-0170, USA

###### **Correspondence:** Nina Sperber

**Background**

Precision medicine represents a rapidly growing field, and healthcare systems must be prepared to incorporate evidence-based applications into routine health care; however, there is variability in uptake of clinically actionable recommendations. We compare and contrast implementation challenges across six projects within an NIH-funded network, IGNITE (Implementing GeNomics In pracTicE), using the Consolidated Framework for Implementation Research (CFIR) and associated strategies mapped to the Expert Recommendations for Implementing Change (ERIC) typology. IGNITE projects focus on implementations of diverse interventions (pharmacogenomics, family health history, genetic testing for disease diagnosis/risk assessment) in varied settings (outpatient clinics, university health systems, federal safety net).

**Methods**

We employed a qualitative case study approach to explore how these diverse projects are implementing genomic services into routine care and a matrix-driven, cross-case synthesis to identify common challenges and highlight the CFIR constructs they represent. Using the ERIC typology, we determined which strategies best addressed these challenges; the strategies used across the diverse projects are described.

**Findings**

Common challenges were: 1) integrating programs with the electronic health record (EHR) in the face of other health system priorities (CFIR construct: relative priority): strategies to address this vary across the projects, with one project targeting structural changes at the health system level to adopt new data standards and sharing protocols while other projects are targeting change at local levels, adapting to the current environment using data warehousing techniques to integrate records or data experts to inform management on data usage; 2) clinician knowledge of genomics (CFIR construct: knowledge and beliefs): all projects developed educational materials and conducted educational meetings for clinicians, though specific strategies varied; 3) recruiting patients (CFIR construct: engaging stakeholders): three projects actively involved patients in implementation (e.g., a patient advisory board to develop educational materials) and three projects developed materials to inform patients about questions to ask their clinician or payer. Thus, though projects are diverse, they share challenges at the system, clinician, and patient levels.

**Implications for D&I Research**

Collaborative evaluation of six diverse genomics projects highlight challenges and strategies to address those challenges – information that is essential for precision medicine to achieve its potential for improving the health and well-being of patients.

**Primary Funding Source**

National Institutes of Health - U01

### S71 Insurance coverage policies for guideline-recommended genetic testing for cancer targeted therapies: preliminary results

#### Christine Lu^1^, Rachel Ceccarelli^2^, Kathleen M. Mazor^3^, Ann Wu^1,2^

##### ^1^Department of Population Medicine, Harvard Medical School and Harvard Pilgrim Health Care Institute, Boston, MA, 02215, USA; ^2^Department of Population Medicine, Harvard Pilgrim Health Care, Boston, MA, 02215, USA; ^3^Medicine, University of Massachusetts Medical School, Worcester, MA, 01605, USA

###### **Correspondence:** Christine Lu

**Background**

Modern medicine is transitioning from empirical treatment to treatment on the basis of the underlying biology of the disease through the use of genomics-based technologies. Genomic tests are the fastest growing sector of medicine and medical science and have the potential to improve clinical practice. This study examined differences in patient access to guideline-recommended genetic tests that guide cancer treatment.

**Methods**

We reviewed publicly available coverage policies for 22 gene-cancer drug pairs (e.g., HER2, EFGR, BRAF tests), including 10 large private insurers and 12 Medicare contractors. We searched gene and drug names and key terms including gene, genomic, and biomarker. We reviewed and extracted the following features: type of policy (gene specific, drug specific, generic policy for genetic testing); medical condition for which the test is covered; requirements for prior authorization; test methods & test result definition; and evidence basis for coverage.

**Findings**

Across 10 private insurers, we identified 18 gene-specific policies, 63 drug-specific policies, 36 prior authorization requirements (for genetic test, drug, or both), and 16 general policies for groups of genetic tests. Overall, some insurers have established gene-specific coverage policies that guide use of such tests, while most relied on policies guiding coverage of genetic testing more generally that may or may not include guideline-recommended genetic tests of interest. A few insurers only have drug coverage policies and some of these did not recommend use of evidence-based genetic testing. Further analysis of policies is underway.

**Implications for D&I Research**

Substantial variations in how insurers are addressing guideline-recommended genetic tests exist. These preliminary findings underscore the need for a better understanding of barriers to implement genetic testing that have potential to improve cancer care and outcomes.

**Primary Funding Source**

National Institutes of Health - R21HG008510, Genomics-based Technologies: Access and Reimbursement Issues

### S72 Implementing a Genomefirst(TM) approach to precision medicine

#### Alanna Kulchak Rahm, Adam H. Buchanan, Marci Schwartz, Cara McCormick, Kandamurugu Manickam, Marc S. Williams, Michael F. Murray

##### Genomic Medicine Institute, Geisinger Health System, Danville, PA, 17822, USA

###### **Correspondence:** Alanna Kulchak Rahm

**Background**

For the promise of precision medicine to be realized, genomic information must be effectively incorporated into routine clinical care. Geisinger Health System (GHS) has implemented a GenomeFIRSTÔ protocol to return results for 76 genes associated with 27 medically actionable conditions to participants in the MyCodeÒ Community Health Initiative biobank. Here we report on the development and implementation of the GenomeFIRST program, including barriers, facilitators, and lessons learned in the process of integrating genomic medicine in routine care.

**Methods**

GHS patients are approached at any clinic visit to participate in MyCodeÒ. Consent includes participation in the biobank, consent to have blood drawn for whole exome sequencing (WES), and to be recontacted if medically actionable genomic information is found. The return-of-results protocol includes patient notification that a genomic result is available and an offer to discuss the results and recommended management with a genomics or non-genomics clinician. Results are placed in patients’ electronic health record. Messages notifying primary care providers of the patient’s result include a link to an online CME module and clinical guidance for management and identification of family members.

**Findings**

WES has been completed in 52,726 of the 110,000 MyCode participants. Results have been returned to 147 individuals for 11 conditions (comprising 21 genes) thus far. Implementing genomic medicine within a routine care is complex and relatively uncharted territory; therefore an iterative implementation process was necessary to address unanticipated issues as they arose. During the return of the first 100 results, changes were made to the process of notifying providers, notifying patients, and facilitating clinical follow up for patients and their at-risk relatives. Such changes involved adding a primary care liaison, streamlining interactions with non-genomic clinicians, and streamlining patient processes and follow-up options.

**Implications for D&I Research**

Implementation of precision medicine programs incorporating genomic data into clinical practice can be done in the context of a healthcare system utilizing clinical processes for sample collection and follow up after return of results. Processes for returning results and collaborating with primary care physicians may need to be tailored to the context of individual healthcare systems and must be adaptable to the rapidly changing environment of genomics.

**Primary Funding Source**

Other (please specify below) - this work is supported in part by a research grant from the Regeneron Genetics Center

## Prevention and Public Health

### S73 A scoping study of program adaptation frameworks for evidence-based interventions

#### Ngoc-Cam Escoffery^1^, Erin Lebow-Skelley^1^, Hallie Udelson^1^, Elaine Böing^1^, Maria E. Fernandez^2^, Richard J. Wood^3^, Patricia Dolan Mullen^2^

##### ^1^Behavioral Sciences and Health Education, Rollins School of Public Health, Emory University, Atlanta, GA, 30322, USA; ^2^Health Promotion and Behavioral Sciences, University of Texas School of Public Health, Houston, TX, 77578, USA; ^3^Center for Health Promotion and Prevention Research, University of Texas School of Public Health, Houston, TX, 77030, USA

###### **Correspondence:** Ngoc-Cam Escoffery

**Background**

Evidence-based public health translation of research to practice is essential to improving the public’s health. Dissemination and implementation researchers have explored what happens once practitioners adopt evidence-based interventions (EBIs). Organizations sometimes make changes, or adaptations, to the original EBI to fit their needs. Researchers have developed models and frameworks to provide a process for adapting EBIs. This scoping study identified and summarized these adaptation frameworks and was guided by the following questions: 1) What are adaptation frameworks used in research and practice? and 2) What are the common adaptation steps suggested across the adaptation frameworks?

**Methods**

We followed the six recommended steps of a scoping study: 1) identifying the research question; 2) identifying relevant studies; 3) selecting studies; 4) charting the data; 5) collating, summarizing, and reporting the results; and 6) consulting with experts. We identified frameworks when searching PubMed, PsycINFO, PsycNET and CINAHL databases for published and grey literature, and from reference lists of framework articles. One researcher coded the frameworks and their steps into Excel and grouped common steps. Two researchers then reviewed and created the suggested names and descriptions for the final included adaptation steps.

**Findings**

Thirteen adaptation frameworks were found, including two from the grey literature and 11 from the published literature. Eleven program adaptation steps were identified and grouped into the following categories: 1) assess community, 2) understand the EBI(s), 3) select intervention, 4) consult with experts, 5) consult with stakeholders, 6) decide on needed adaptations, 7) adapt the original EBI, 8) train staff, 9) test the adapted materials, 10) implement the adapted EBI, and 11) evaluate. Eight of these steps were recommended by more than five frameworks: #1-3, 6-7, and 9-11.

**Implications for D&I Research**

This study is the first to identify common adaptation frameworks or models for EBIs. It contributes to the literature by consolidating key steps in the approach to program adaptation of EBIs, describing the associated tasks in each step, and recommending standard steps suggested by the frameworks. We also identify research gaps in understanding how to perform adaptations and contributions of these frameworks to the science of translation of EBIs.

**Primary Funding Source**

National Institutes of Health - National Cancer Institute

### S74 Addressing the adherence-adaptation debate: lessons from the replication of an evidence based sexual reproductive health program

#### Jenita Parekh^1,2^, Valerie Caldas^1^, Elizabeth A. Stuart^3^, Shalynn Howard^4^, Gilo Thomas^5^, Jacky M Jennings^2^

##### ^1^Center for Child Community Health Research, Johns Hopkins School of Medicine, Baltimore, MD, 21224, USA; ^2^Department of Population, Family, and Reproductive Health, Johns Hopkins Bloomberg School of Public Health, Baltimore, MD, 21224, USA; ^3^Department of Mental Health, Johns Hopkins Bloomberg School of Public Health, Baltimore, MD, 21205, USA; ^4^Medicine, Johns Hopkins University, Baltimore, MD, 21224, USA; ^5^Division of Family Health Services, New Jersey Department of Health, Trenton, NJ, 08625, USA

###### **Correspondence:** Jenita Parekh

**Background**

Whether high adherence to programs is necessary to achieve program outcomes is an area of great debate; and, determining if adaptations reduce program effects is critical to moving the implementation science field forward. The objectives of this study were 1) to determine the frequency and type adaptations made in the implementation of an evidence based program, and 2) to determine program outcomes (i.e. knowledge and behavioral intent) for intervention program participants, as compared to comparison participants, by the level of adaptations made in the classroom (high/middle/low adaptation).

**Methods**

A mixed methods approach was used to study the implementation of a sexual reproductive health curriculum in 45 classrooms, with a total of 1,608 participants. Frequency calculations from developer-created fidelity logs were used to describe % adaptation by classroom. Thematic qualitative analysis was used to categorize types and rationales for adaptation. Program outcomes by level of adaptation were determined using logistic regression analyses and mean differences

**Findings**

Adaptations ranged from 2%- 97% across classrooms, with mean adaptation of 46%. Thematic analysis revealed that the adaptations made were related to delivery of content, rather than to the content itself and in response to participant needs and setting constraints. Program outcomes comparing the intervention condition to the comparison condition for the low, middle, and high adaptation groups were as follows: SRH knowledge score difference between intervention vs control [low = +14.3%, middle = +17.4% , high = 17.8%], intent to use birth control in next 6 months [low: OR = 2.29 (1.28-4.09), p = .01; middle: OR = 2.36 (1.09-4.13), p = .01; high: OR = 5.67 ( 2.51-12.85), p = .00]; intent to abstain from sex [low: OR = 1.63 (.80-3.30), p = .17; middle: OR = 1.43 (.79-2.61), p = .23; high: OR = 1.34 (.69-2.63), p = .37]; intent to use condoms in the next 6 months [low: OR = 2.04 (1.11-3.76), p = .04; middle: OR = 2.36 (1.09-4.13), p = .04; high: OR = 5.67 (2.51-12.85), p = .04].

**Implications for D&I Research**

Program outcomes did not appear to be reduced for the high adaptation subgroup. Understanding both rationale (intent) and type of adaptation made is crucial to understanding the complexity of adaptations. These finding also support the argument for allowing facilitators some flexibility and autonomy to adapt the delivery of prescribed content of EBPs to participant needs and setting constraints.

### S75 Online decision support to facilitate adoption of sexual health curricula for AI/AN communities

#### Jennifer Torres^1^, Christine Markham^1^, Ross Shegog^1^, Melissa Peskin^1^, Stephanie Craig Rushing^2^, Amanda Gaston^3^, Gwenda Gorman^4^, Cornelia Jessen^5^, Jennifer Williamson^5^

##### ^1^Health Promotion and Behavioral Sciences, The University of Texas Health Science Center School of Public Health, Houston, TX, 77030, USA; ^2^NW Tribal Epidemiology Center, Northwest Portland Area Indian Health Board, Portland, OR, 97201, USA; ^3^Project Red Talon, Northwest Portland Area Indian Health Board, Portland, OR, 97201, USA; ^4^Health and Human Services, Inter Tribal Council of Arizona, Inc., Phoenix, AZ, 85004, USA; ^5^HIV/STD Prevention Program, Alaska Native Tribal Health Consortium, Anchorage, AK, 99508, USA

###### **Correspondence:** Jennifer Torres

**Background**

There are few evidence-based sexual health programs for American Indian/Alaska Native (AI/AN) youth and even fewer tools available to assist AI/AN communities with adopting, implementing, and maintaining such programs despite documented sexual health disparities. iCHAMPSS (Choosing And Maintaining effective Programs for Sex education in Schools) is an innovative, theory- and web-based decision-support-system that provides tailored step-by-step guidance needed to overcome dissemination barriers and increase reach and fidelity of evidence-based programs (EBPs), specifically for the promotion of sexual health in schools. The goals of this study are to: 1) pilot-test iCHAMPSS for usability and psychosocial impact with adult stakeholders working with AI/AN communities; and 2) determine whether it would be acceptable to adapt iCHAMPSS for AI/AN communities based on usability results.

**Methods**

Adult stakeholders working in AI/AN communities across the country were recruited (N = 36) and asked to review selected tools accessible through the iCHAMPSS website for 2 weeks. Pre- and post-surveys were administered to measure usability parameters and short-term psychosocial outcomes. Data were analyzed using descriptive statistics, the Wilcoxon Signed-Rank test and McNemar’s test.

**Findings**

iCHAMPSS was perceived as more helpful than current resources (75%), credible (86-94%), appealing (81-86%), impactful of EBP adoption, implementation, and maintenance (89-94%), and impactful of individual and organizational determinants of EBP adoption (64-89%). Compared to current practice, when choosing, implementing, and maintaining EBPs, iCHAMPSS was rated as significantly more thorough, efficient, and facilitative of improved communication with schools and communities (all p < 0.05), and was rated easier and faster for choosing and maintaining EBPs (p < 0.05). Conversely, iCHAMPSS significantly increased participants’ perceived barriers to adopting an EBP (*p <* 0.05). Selected iCHAMPSS tools were reported to be acceptable (78-94%), easy to use (78-94%), useful (64-100%), and appealing (81-86%). Qualitative feedback revealed that some users were overwhelmed by the amount of information, though the majority appreciated the organization and content.

**Implications for D&I Research**

Overall, adult stakeholders working with AI/AN communities responded positively to iCHAMPSS. This usability study supports that iCHAMPSS is an acceptable innovative decision-support system that could be adapted for AI/AN communities to expand the dissemination and implementation of sexual health EBP’s in Indian country.

**Primary Funding Source**

Funding was awarded to enhance school and community capacity to implement an innovative comprehensive teen pregnancy, HIV/STI prevention program for early adolescent AI/ANs.

### S76 Go NAP Sacc: testing an online, interactive tool to improve nutrition environments at child care

#### Dianne Ward^1,2^, Amber Vaughn^2^, Ellie Morris^2^, Stephanie Mazzucca^1^, Regan Burney^2^

##### ^1^Nutrition, University of North Carolina, Chapel Hill, NC, 27599, USA; ^2^Center for Health Promotion and Disease Prevention, University of North Carolina, Chapel Hill, NC, 27599-7426, USA

###### **Correspondence:** Dianne Ward

**Background**

As part of the nation’s call to action to fight childhood obesity, the Institute of Medicine recommends that childcare centers (CC) implement evidence-based strategies to promote healthier eating and physical activity habits in children. Translation of evidence-based interventions into real world CC settings often encounter barriers, including costs, time constraints, and needs for training and technical assistance. This study describes translation of an evidence-based program (NAPSACC) into an online format (Go NAPSACC), implementation of the online program by CC directors, and evaluation of its impact on CC nutrition environments.

**Methods**

Go NAPSACC retained core elements of the original program, but translated tools into an online, self-directed format using extensive input from the practice community. Local CC quality improvement agencies facilitated recruitment of 33 CC programs which were randomized to immediate access (IA, n = 17) or delayed access (DA, n = 16) groups. CC directors received an initial training on Go NAPSACC tools from agency staff trained by the researchers. Directors were then encouraged to implement Go NAPSACC independently with minimal additional assistance. Online tools included a self-assessment of CC nutrition provisions, practices, and policies followed by automated feedback that encouraged change thinking. Goal selection and action planning tools guided directors in setting priorities and creating an action plan for change. The tips and materials library provided a curated collection of resources to support changes. The Environment and Policy Assessment and Observation (self-report), collected prior to and following the 4-month intervention period, assessed impact on CC's nutrition environments.

**Findings**

Demographic characteristics of IA and DA centers were similar. One CC from each group did not engage in any implementation activities. Comparing baseline to follow-up among centers with teacher stability (29/31), IA centers improved overall nutrition scores (effect size = 0.83), as well as scores for foods (0.77), beverages (0.51), environment (0.47), and menus (0.63) compared to DA, controlling for CACFP participation and quality rating.

**Implications for D&I Research**

Core elements of NAPSACC were effectively translated into online tools and successfully implemented by CC directors. The online program retained its ability to drive change in CCs’ nutrition environments using a streamlined, self-directed, and flexible implementation approach.

**Primary Funding Source**

The Robert Wood Johnson Foundation - This project was funded by Healthy Eating Research (Round 8) and was an evaluation of Go NAP SACC: A web-based tool based on the NAP SACC program.

### S77 The promise and challenge of building practitioner networks to support implementation of evidence-based programs in community settings

#### Shoba Ramanadhan^1,2,4^, Sara Minsky^1^, Vilma Martinez-Dominguez^3^, Kasisomayajula Viswanath^1,4^

##### ^1^Center for Community-based Research, Dana-Farber Cancer Institute, Boston, MA, 02215, USA; ^2^Population Health Research Program, Harvard Catalyst: The Harvard Clinical and Translational Science Center, Boston, MA, 02115, USA; ^3^City of Lawrence Mayor's Health Task Force, Lawrence, MA, 01840, USA; ^4^Social and Behavioral Sciences, Harvard T.H. Chan School of Public Health, Boston, MA, 02215, USA

###### **Correspondence:** Shoba Ramanadhan

**Background**

Insufficient capacity to use evidence-based programs (EBPs) limits the impact of community-based organizations (CBOs) to improve population health and mitigate health disparities. To address this capacity gap, a team of practitioner-leaders and researchers conducted PLANET MassCONECT, a community-based participatory research project based in three Massachusetts communities. The team co-created an intervention to build capacity among CBO staff members to systematically find, adapt, and evaluate EBPs. In addition to investing in human capital, the project supported development of social capital in the form of social networks among trainees.

**Methods**

Cohorts of trainees were enrolled from June 2010 to April 2012. We conducted sociometric, or whole-network, analyses in each of the three partner communities in late 2013, using a web-based survey. The network relationship of interest was communication regarding the systematic approach to program planning presented in the intervention.

**Findings**

For Communities A, B, and C, 38/59, 43/61, and 51/ 59 trainees responded, respectively. Only the response rate in Community C was sufficient to support a network analysis. The density of the Community C communication network (or proportion of potential connections realized) was 10% and the average degree (number of connections with other trainees) was 5.73. We found a statistically significant association between degree, used here as a marker of network engagement, and use of EBPs in practice in the six months preceding the assessment, adjusting for important covariates (β = 0.11, p = 0.002; adjusted R^2^ = 0.34). We also found that trainees shared intervention content with networks of colleagues outside the program.

**Implications for D&I Research**

The results prompt further research into the potential for infrastructure development (here, in the form of practitioner networks) to support use of research evidence in community settings. Careful study of and adjustment for contextual factors, including availability of relevant resources, turnover rates within CBOs, and community complexity will be critical for intervention success and building the science of using research evidence to create change in communities.

**Primary Funding Source**

National Institutes of Health - R01CA132651

### S78 TEACH: Operationalizing the Knowledge to Action framework to promote practice change

#### Megan Barker^1,2^, Myra Fahim^1,3^, Arezoo Ebnahmady^1^, Rosa Dragonetti^1^, Peter Selby^1,2,4,5,6^

##### ^1^Nicotine Dependence Service, Centre for Addiction and Mental Health, Toronto, ON, M5T1P7, Canada; ^2^Dalla Lana School of Public Health, University of Toronto, Toronto, ON, M5T3M7, Canada; ^3^School of Social Work, University of Windsor, Windsor, ON, N9A 0C5, Canada; ^4^Addictions Program, Centre for Addiction and Mental Health, Toronto, ON, M5T1P7, Canada; ^5^ Family and Community Medicine, University of Toronto, Toronto, ON, M5G1V7, Canada; ^6^ Psychiatry, University of Toronto, Toronto, ON, M5T1R8, Canada

###### **Correspondence:** Megan Barker

**Background**

TEACH (Training Enhancement in Applied Cessation Counselling and Health) is a knowledge translation (KT) project offering Canada’s first interprofessional, university-accredited certificate program in tobacco dependence treatment. Recognizing that KT is an iterative and dynamic process, the Knowledge to Action Framework, was used to operationalize the development and application of TEACH as a Continuing Medication Education (CME) Program.

**Methods**

TEACH KT activities follow the Knowledge-to-Action cycle. TEACH developed a 43.5 hour certificate program (both in-person and online) by synthesizing best-practice approaches in cessation interventions, implementing revisions on an iterative basis. Pre- and post-course learning assessments helped practitioners to identify/review/select knowledge by setting clinically relevant practice objectives. Practitioners adapted knowledge to their local context through the application of content to their own practice (i.e. case examples, reflections), and barriers to knowledge use were assessed through 3- and 6-month follow up surveys, post-training. To facilitate and sustain KT post-training, a menu of continuing education supports were developed to form a TEACH Community of Practice (CoP). Supports included (1) an email Listserv fostering information exchange between TEACH-trained practitioners and subject matter experts (2) adhoc coaching and consultation with TEACH faculty (3) monthly accredited educational rounds on complex clinical issues (4) clinical video vignettes and resources, and (5) train-the-trainer toolkits to build capacity in organizations and local communities.

**Findings**

Since 2006, over 5000 practitioners have attended TEACH trainings. Learning assessment data showed significant increase in self-reported feasibility, importance and confidence of changing practice and 94% set practice goals. At 6 month follow-up, 71.9% reported offering cessation interventions, and 90.5% reported KT activities in their organizations/communities. 730 individuals are currently subscribed to the TEACH Listserv, over 750 toolkits have been disseminated to practitioners and 92 webinars have been offered to date.

**Implications for D&I Research**

Few CME programs have been developed through the operationalization of a KT theoretical model. Findings suggest that using a model to inform the development of a CME program can facilitate and sustain practice change.

**Primary Funding Source**

Ontario Ministry of Health and Long Term Care

### S79 A thematic content analysis of practitioners' inquiries about public health research and interventions: implications for enhancing dissemination

#### Margaret Farrell, Jordan Tompkins, Wynne Norton, Kaelin Rapport

##### Implementation Science Team, Division of Cancer Control and Population Sciences, National Cancer Institute, Rockville, MD, 20850, USA

###### **Correspondence:** Wynne Norton

**Background**

Improving communication and strengthening collaboration between researchers and public health practitioners is critical for effectively disseminating and implementing evidence-based interventions. To this end, NCI’s Research to Reality (R2R) online community of practice convenes researchers and practitioners around a shared commitment to implement evidence-based, cancer-related (e.g., physical activity, tobacco control, screening, prevention) interventions in public health settings. Several dynamic approaches are used to facilitate dynamic communication and collaboration, including monthly webinars, question and answer (Q&A) sessions, and discussion posts.

**Methods**

This study explored the programmatic considerations most important to R2R community members through a content analysis of webinar Q&A sessions and related discussions. We used deductive coding for segments of text related to implementation strategies (Powell et al 2015; Wang 2016) and intervention adaptation (Wiltsey-Stirman et al, 2012), and inductive coding to explore thematic patterns from participants’ questions. Team members coded, sorted, and analyzed the data. Interrater reliability was ensured by having the team members code 9 documents and compare applications of the codes to ensure consistent use. The final coding schema was applied to 62 transcripts of webinar presentations (Q&A section only) and the related online discussions over a 6-year period (2010-2016).

**Findings**

Several themes emerged from the content analysis, including inquiries around financial and institutional support, program sustainability, program tools and resources. Community members requested additional information from presenters about contextual variables of research studies (e.g., the role of partnerships in implementing initiatives and adapting programs to reach underserved populations) as well as evidence-based programs and tools (e.g. evaluation measures). Practitioners consistently sought to better understand variables that made an intervention effective and often requested guidance on the most appropriate implementation strategies for their programs.

**Implications for D&I Research**

While a number of resources are designed to implement evidence-based health programs, the strategies and programmatic considerations most salient to practitioners are difficult to elicit but essential to address. These data highlight additional information researchers should include in their intervention presentations, summaries and publications when communicating with practitioners. By addressing the priority concerns of practitioners, researchers may be able to improve communication and subsequently enhance the dissemination and appropriate use of evidence-based public health interventions.

**Primary Funding Source**

National Institutes of Health

### S80 Preventing and mitigating the effects of adverse childhood experiences by building community capacity and resilience: appi cross-site evaluation findings and collective capacity measurement

#### Margaret Hargreaves

##### Public Health, Community Science, Acton, MA, 01720, USA

**Background**

ACEs (adverse childhood experiences) are a complex population health problem with significant detrimental outcomes. In 2013, the ACEs Public-Private Initiative (APPI) was formed to study effective interventions to prevent and mitigate ACEs, increase resilience, and facilitate fieldwide learning. APPI sponsored a rigorous mixed methods evaluation of five community-based initiatives. The evaluation's findings were issued in July 2016.

**Methods**

The evaluation conducted by Mathematica Policy Research and Community Science sought to understand the sites' strategies, the extent to which the sites developed collective community capacity to initiate program, policy and systems change addressing ACEs, and assessed the impact of their efforts. The mixed methods evaluation included qualitative (1) site visits, (2) key informant interviews, and (3) reviews of site documents. The quantitative methods included: (1) an analysis of county-level trends of 30 ACEs-related indicators that compared the sites to the rest of the state; (3) development and implementation of the valid and reliable evidence-based ARC3 survey, measuring ten domains of site capacity at four levels (coalition, network, community solutions and communitywide impact); and (4) quantitative analyses of individual-, program-, and organization-level changes associated with 11 selected site activities, using rigorous quasi-experimental methods.

**Findings**

The evaluation found that six of the 11 site activities were associated with positive and statistically significant changes in targeted outcomes, including (1) maternal and child outcomes in the Nurse Family Partnership program, (2) decreased alcohol use among youth in the Positive Social Norms Campaign, (3) improved school attendance of children referred for truancy to the Community Truancy Board, (4) 40% communitywide awareness of ACEs concepts in an ACEs and Resilience Awareness Campaign, (5) improved community conditions in the Commitment of Community initiative, and (6) reduction in suspensions and increased graduation rates among students in an alternative high school. The APPI sites that were more successful in addressing ACEs and toxic stress and building resilience aligned three factors: (a) collective community capacity, (b) community network characteristics, and (c) effective community change strategies.

**Implications for D&I Research**

The evaluation provides new valid and reliable capacity measurement methods and rigorous outcome evaluation methods for evaluating the success of community prevention initiatives.

**Primary Funding Source**

The evaluation and collective capacity survey were funded by the APPI consortium of foundations

### S81 Investigating implementation of a partnership approach for community-based health promotion: a mixed methods evaluation of the Massachusetts prevention and wellness trust fund

#### Rebekka Lee^1,4^, Shoba Ramanadhan^1,2^, Gina Kruse^3,4^, Charles Deutsch^4^

##### ^1^Social and Behavioral Sciences, Harvard T.H. Chan School of Public Health, Boston, MA, 02115, USA; ^2^Center for Community-based Research, Dana-Farber Cancer Institute, Boston, MA, 02215, USA; ^3^Division of General Internal Medicine, Massachusetts General Hospital, Boston, MA, 02114, USA; ^4^Population Health Research Program, Harvard Catalyst: The Harvard Clinical and Translational Science Center, Boston, MA, 02115, USA

###### **Correspondence:** Rebekka Lee

**Background**

Utilizing partnerships is a promising approach for integrating evidence-based prevention interventions within clinical and community-based settings. In 2012, the Massachusetts legislature funded The Prevention and Wellness Trust Fund (PWTF), which provides more than $42 million over four years, to address hypertension, pediatric asthma, elder falls, and tobacco use via nine local partnerships throughout the state. We seek to leverage implementation science, social network analysis, and a mixed methods design to understand these how these partnerships function and what contextual factors are most influential on successful implementation. The aim of this abstract is to describe the multi-phase explanatory mixed methods study design embedded in a large evaluation being used to investigate PWTF implementation.

**Methods**

This study is grounded in the Consolidated Framework for Implementation Research. First, 1.5-hour semi-structured qualitative interviews were conducted with two leaders from each partnership to document partnership development and function, intervention adaptation and delivery, and the influence of contextual factors on implementation. These results informed adaptation of a validated quantitative survey to assess the implementation experiences of approximately 175 staff from clinical and community-based settings and a social network analysis to assess changes in the relationships among 90 PWTF partner organizations. The quantitative survey data was used to select 24 staff from 4 "high implementation" partnerships who were interviewed to explore the most successful and challenging experiences of implementing evidence-based interventions for the four conditions. Individuals surveyed and interviewed included clinical staff of varying levels, practitioners in community-based settings, and community health workers.

**Findings**

This study describes how a mixed methods approach can be used to incorporate the investigation of context, allow for exploration of unanticipated consequences and systems changes, and integrate end-users voices into an existing evaluation.

**Implications for D&I Research**

Data from the study will speak to a range of constituents—from scientists to policymakers to public health and clinical practitioners. Description of the study design, measures, and theoretical framework will help researchers plan mixed methods research to investigate the implementation of complex evidence-based interventions. Results will help communities in Massachusetts and throughout the country who want to learn about the implementation of community-clinical partnerships for the promotion of population health.

### S82 Implementing evidence-based resources on childhood obesity in Medicaid managed care

#### Emily Lanier, Ashley Gray

##### Institute for Medicaid Innovation, Institute for Medicaid Innovation, Washington, DC, 20036, USA

###### **Correspondence:** Emily Lanier

**Background**

Roughly one-third of children over two years are overweight or obese, reaching as high as 39 percent for low-income children enrolled in federally funded health programs (e.g., Medicaid). Current systematic reviews demonstrate a lack of evidence to assess the efficacy of non-school based interventions, like those implemented by Medicaid managed care organizations (MMCOs), in preventing childhood obesity, especially among those disproportionately impacted by the social determinants of health.

**Methods**

We used a mixed methods study design to explore barriers and strengths of current implementation strategies around childhood obesity prevention and treatment (CHOPT) initiatives that included a national survey of 39 MMCOs and the collection of five case studies of innovative CHOPT initiatives. We also conducted interviews with 28 families who participated in one of the five case study initiatives to gain an understanding of the impact of the social determinants of health on a family’s ability to sustain healthy lifestyle changes. Finally, we developed an implementation toolkit based on findings from the questionnaire, case studies, and family interviews that includes a validated AHRQ readiness assessment and CMS implementation tools to guide MMCOs through the design, implementation, and evaluation of CHOPT initiatives.

**Findings**

Survey responses were collected from 39 MMCOs, representing 38 states and DC. Survey respondents identified clinical and policy barriers (e.g., reimbursement mechanisms, sustainability challenges, lack of clinical guidelines) that need to be addressed to maximize successful implementation of CHOPT initiatives. Furthermore, the family interviews identified interconnected factors (i.e., time, motivation, access, and support) that impact the efficacy of the obesity prevention and treatment initiatives and influence a family’s ability to participate. Currently, MMCOs address some of these barriers by relying on critical relationships with community resources and key stakeholders.

**Implications for D&I Research**

This study represents the first opportunity for clinicians, MMCOs, and families to identify the barriers to implementing and participating in CHOPT interventions and highlight lessons learned in program design and community resource development and strategies to reduce the impact of social determinants of health. The CHOPT toolkit increases awareness of MMCO-led childhood obesity interventions and provides the foundation for policy solutions that will maximize implementation of CHOPT initiatives.

**Primary Funding Source**

The Robert Wood Johnson Foundation - The Agency for Healthcare Research and Quality (AHRQ) and the Center’s for Medicare and Medicaid Services (CMS)’s Office of Minority Health collaborated on this project.

### S83 Using community-based participatory research to develop a state-wide model for health promotion program implementation in Minnesota

#### Aaron Leppin^1,2^, Lori Christiansen^3^, Karen Schaepe^2^, Jason Egginton^2^, Megan Branda^2^, Charlene Gaw^4^, Sara Dick^1^, Victor Montori^5^, Nilay Shah^2^

##### ^1^Knowledge and Evaluation Research Unit, Mayo Clinic, Rochester, MN, 55905, USA; ^2^Health Sciences Research, Mayo Clinic, Rochester, MN, 55905, USA; ^3^SE MN Area Agency on Aging, Rochester, MN, 55902, USA; ^4^Mayo Medical School, Rochester, MN, 55905, USA; ^5^Health Care and Policy Research, Mayo Clinic, Rochester, MN, 55905, USA

###### **Correspondence:** Aaron Leppin

**Background**

Evidence-based Health Promotion Programs, such as the Diabetes Prevention Program and the Chronic Disease Self-Management Program, support people's efforts to manage their health. They improve outcomes and reduce costs. Because they are offered in community settings and by lay leaders outside the traditional healthcare system, they are challenging to implement.

**Methods**

We conducted a mixed-methods, community-engaged and participatory research project focused on CDSMP implementation in an 11-county region in Southeast Minnesota and informed by the PRECEDE implementation planning framework. As part of this, we developed 5 inter-silo working groups of healthcare, public health, and community stakeholders that focused on brand development, community capacity building, clinical integration and referral procedures, strategy and sustainability, and research. The research working group interviewed and surveyed groups of primary care clinicians and non-clinician stakeholders across the region. Insights from this research were iteratively integrated with the activities of the other working groups and developed into a cohesive implementation strategy.

**Findings**

The integrated quantitative and qualitative data identified a “missed opportunity” for clinic-community collaboration for improving population health. Major themes—Two Systems, Two Worlds; Not My Job; and Use Your Imagination—emerged from the interaction of the PRECEDE codes (e.g. Predisposing, Reinforcing, Enabling, Administrative, and Policy factors) with each other and across clinician (n = 220) and stakeholder respondents (n = 93). We used this insight to develop a regionally-confined and replicable framework for creating a “community system” for evidence-based programs. Key components of the associated implementation strategy were targeted clinician education, establishment of a “community system,” and the partnering of multiple community-based organizations under a single brand and mission. This framework and strategy is now being integrated into a funded, 3-year project focused on state-wide implementation. Key components of the scalability strategy were engagement of stakeholders at the state level and in pre-existing networks from the outset, and the use of common and replicable infrastructures.

**Implications for D&I Research**

Our study suggests that, through proactive engagement of stakeholders, it is possible to develop scalable implementation strategies for health promotion that meet the timelines of a rapidly changing policy environment.

**Primary Funding Source**

National Institutes of Health - NCATS funding through Institutional CTSA

### S84 Use of group model building to develop implementation strategies for early childhood obesity prevention

#### Ariella Korn^1^, Peter Hovmand^2^, Karen Fullerton^1^, Nancy Zoellner^2^, Erin Hennessy^3^, Alison Tovar^4^, Ross Hammond^5^, Christina Economos^3^

##### ^1^Tufts University Friedman School of Nutrition Science and Policy, Boston, MA, 02111, USA; ^2^Social System Design Lab, Washington University Brown School of Social Work, St. Louis, MO, 63130, USA; ^3^ChildObesity180, Tufts University Friedman School of Nutrition Science and Policy, Boston, MA, 02111, USA; ^4^Nutrition and Food Sciences, University of Rhode Island, Kingston, RI, 02881, USA^5^; Center on Social Dynamics and Policy, Brookings Institution, Washington, DC, 20036, USA

###### **Correspondence:** Ariella Korn

**Background**

Interventions during early childhood (EC), birth to 5 years, can help establish healthy behaviors that may contribute to healthy weight trajectories throughout life. Despite known effectiveness of existing interventions, communities struggle to implement them within naturalistic settings, and with sufficient reach and scale to achieve population level impact. Group model building (GMB), a participatory method grounded in system dynamics, has been cited as a promising approach for designing and adapting intervention strategies that take into account the inherent complexities of implementation.

**Methods**

This study used GMB within a community-based participatory research (CBPR) context with a multisector steering committee of 16 community and EC providers. The committee was mobilized in October 2015 to guide the design and implementation of a pilot intervention in Somerville, MA. During Year 1, committee members attended eight meetings that included structured GMB activities or ‘scripts’ combined with other CBPR methods (e.g., engagement of committee members in all aspects of intervention development). Research staff planned and facilitated each meeting based on committee feedback, evolving group cohesion, and GMB best practices to promote insight sharing, consensus, and effective intervention design.

**Findings**

Meeting attendance averaged 11.5 committee members (72% attendance rate). For those absent, 94% attended make-up meetings. During meetings, GMB was used to a) establish group expectations for activities and community impact, b) identify EC health priorities and connections between them, c) assess feasibility and impact of evidence-based interventions, and d) propose key messages, activities and resources to disseminate a cohesive, whole-of-community obesity prevention campaign. These activities facilitated committee members’ awareness of their unique roles in the community, connection between roles, and enhanced collaborative multisector efforts to prevent EC obesity. The committee plans to implement locally- and culturally-appropriate interventions (i.e., programs, practices, and policies) during Year 2 that aim to impact healthy weight trajectories of young children.

**Implications for D&I Research**

Using GMB within a CBPR context supports efficient and effective development of implementation strategies. By prioritizing a highly participatory research design with buy-in from key stakeholders, community-based interventions can be equipped to tackle complex and dynamic public health problems and offer sustainable solutions.

**Primary Funding Source**

National Institutes of Health - This work was supported by NHLBI and OBSSR (R01HL115485) and the Brookings Institution.

### S85 Power up for 30 collaboration and implementation strategies

#### Christi Kay^1^, Julie Gazmararian^2^, Emily Vall^3^

##### ^1^HealthMPowers, Norcross, GA, 30092, USA; ^2^Epidemiology, Rollins School of Public Health, Atlanta, GA, 30322, USA; ^3^Georgia Department of Public Health, Atlanta, GA, 30303-3186, USA

###### **Correspondence:** Christi Kay

**Background**

Physical activity (PA) has many benefits for children including improved cardiovascular fitness, muscular fitness, and decreased levels of body fat. The school is an ideal setting to offer PA since the majority of children aged five to seventeen spend up to half of their waking hours in school. Current PA opportunities include in-classroom PA, physical education (PE), recess, and before and after school programs.

**Methods**

We will describe the collaborative approach between academic researchers, a community organization, and governmental agencies to adapt and disseminate the evidence-based Comprehensive School Physical Activity Program for the elementary school setting across the Implementation Stages.

**Findings**

Power Up for 30 (PU30) sought to maximize these PA opportunities by assisting, engaging, and empowering school administrators, PE teachers, and classroom teachers to integrate PA using the Stages of Implementation (exploration, installation, initial implementation, full implementation). The Exploration and Installation Stages occurred during initial PU30 training: The Exploration Stage involved assessment of barriers and facilitators to PA and a baseline assessment of current PA opportunities and environmental characteristics unique to each school. During the Installation Stage, at least one administrator, one grade level chair, and one PE teacher from each school formed a school health team and attended a seven-hour PU30 training. At this training, the implementation team (HealthMPowers) used data from the Exploration Stage to help the school health team explore best practices to overcome barriers to PA specific to each school; draft an action plan; and examine resources to facilitate PA throughout the school day. The Initial Implementation Stage was reinforced by provision of additional resources, ongoing communication, and technical support to school staff to facilitate PA throughout the school day.

**Implications for D&I Research**

The implications for D&I research and the current transition to Full Implementation will be discussed, including: lessons learned from instrument development and process/outcome evaluations; next steps to improve dissemination and ensure sustainability through school-wide trainings, development of a pre-service PU30 certification for PE and early childhood educators, and middle school-focused PU30; and the use of a virtual training to balance of costs and feasibility.

### S86 Impact of Power up for 30 on physical activity opportunity changes: a quasi-experimental study

#### Patricia Cheung^1^, Padra Franks^2^, Shannon Barrett-Williams^2^, Paul Weiss^3^, Christi Kay^2^, Julie Gazmararian^1^

##### ^1^Epidemiology, Rollins School of Public Health, Atlanta, GA, 30322, USA; ^2^HealthMPowers, Norcross, GA, 30092, USA; ^3^Biostatistics and Bioinformatics, Rollins School of Public Health, Atlanta, GA, 30322, USA

###### **Correspondence:** Patricia Cheung

**Background**

To increase physical activity (PA) in the elementary school setting, the Georgia Departments of Public Health and Education along with HealthMPowers adapted the Comprehensive School Physical Activity Program (CSPAP) into the Power Up for 30 (PU30) initiative. This study aimed to ^(1)^ describe the adoption of PU30 across the state and ^(2)^ assess the fidelity of PU30 comparing 79 PU30-trained and 80-untrained schools.

**Methods**

To evaluate adoption of PU30, number of trained schools was compared to number of eligible schools in the state. To evaluate fidelity, a quasi-experimental study was conducted to compare minutes of PA in a subsample of 79 PU30-trained and 80 untrained schools. Survey items indicating frequency and duration of PA opportunities (PE, recess, and classroom-based PA, and before and after school PA) were converted into continuous variables of weekly PA time. Unadjusted analyses and analyses adjusted for baseline PA and school demographics compared PA at follow-up.

**Findings**

As of July 2016, 876 (66%) elementary schools have pledged to integrate 30 minutes of PA into the school day and of these, 726 (54%) are PU30-trained. Within the study sample of 159 schools, trained schools provided 44 more minutes of PA opportunities weekly compared with untrained schools (99% Empirical Interval (EI): 40-48 minutes): Increased PA among trained schools at follow-up was due to trained schools having 16 additional PA minutes in class (99% EI: 16-17), 11 additional minutes before school (99% EI: 10-11) and 9 additional minutes after school (99% EI: 8-9) each week compared with untrained schools. After adjusting for baseline PA and school characteristics, trained schools provided 37 more minutes of PA opportunities weekly compared with untrained schools (99% EI: 32-41), mostly due to in-class PA (mean: 11 additional minutes; 99% EI: 10-11), before school PA (mean: 9 additional minutes, 99% EI: 8-9) and recess (mean: 9 additional minutes, 99% EI: 6-12).

**Implications for D&I Research**

We advance the D&I field by reporting the successful adoption and implementation of PU30, an adaptation of the CSPAP model. One of the first to assess CSPAP in the elementary school, we report increased time for PA during recess, in class, and before school, which will inform other states adapting the CSPAP model.

### S87 The exploration of barriers and facilitators of Power up for 30 initiative: a qualitative evaluation

#### Erica Hamilton^1^, Patricia Cheung^2^, Christi Kay^3^, Emily Vall^4^, Julie Gazmararian^2^

##### ^1^Behavioral Science and Health Education, Rollins School of Public Health, Atlanta, GA, 30322, USA; ^2^Epidemiology, Rollins School of Public Health, Atlanta, GA, 30322, USA; ^3^HealthMPowers, Norcross, GA, 30092, USA; ^4^Georgia Department of Public Health, Atlanta, GA, 30303-3186, USA

###### **Correspondence:** Erica Hamilton

**Background**

The CDC’s evidence based Comprehensive School Physical Activity Program (CSPAP) is a multi-component approach to maximize physical activity (PA) opportunities in the school, but few studies have adopted and evaluated the CSPAP model. This study aimed to examine the barriers and facilitators impacting acceptability, reach, feasibility, and appropriateness of the CSPAP-adapted Power Up for 30 (PU30) initiative among elementary schools across the Atlanta metropolitan area.

**Methods**

Six elementary schools in the metro Atlanta area were recruited from 79 PU30 trained and 80 untrained schools participating in a quasi-experimental study. Principals, physical education (PE) teachers and grade-level chairs were recruited to participate in 30 to 45-minute in-depth individual interviews. Thematic analysis was used to identify and examine patterns related to the acceptability, reach, feasibility and appropriateness of the PU30 initiative. Line-by-line coding, using a combined inductive and deductive approach allowed a comparison of barriers and facilitators between schools. Coding rounds were completed independently by a two-person coding team to ensure inter-coder reliability.

**Findings**

Several barriers and facilitators were identified through the 16 interviews. Barriers impacting acceptability, reach, feasibility and appropriateness included ^(1)^ lack of communication and dissemination of training information between trained and untrained school staff, ^(2)^ difficulty creating “buy-in” from enough teachers to properly sustain the initiative, ^(3)^ limited time for physical activity before, during and after school, and ^(4)^ competing priorities, such as academic standards placed on teachers. Facilitators impacting acceptability, reach, feasibility and appropriateness included ^(1)^ administrative and district-level support, ^(2)^ importance of the students’ health and wellbeing, and ^(3)^ appropriateness of PU30 resources.

**Implications for D&I Research**

This study improves our understanding and knowledge of developing and implementing appropriate and feasible physical activity initiatives in the school system. These findings have directly improved the PU30 initiative and will inform other schools adapting CSPAP by identifying the need for a school-wide training, the importance of continued administrative and district-level support and the significance of prioritizing time for physical activity at school. More research needs to be conducted to better understand how to reduce the barriers and reinforce the facilitators to continue to increase physical activity at school.

## Promoting Health Equity and Eliminating Disparities

### S88 Going off-script: modifications to Cognitive Processing Therapy (CPT) in a community mental health clinic

#### Luana Marques^1^, Louise Dixon^2^, Emily Ahles^3^, Sarah Valentine^4^, Candice Monson^5^, Derri Shtasel^4^, Shannon Wiltsey Stirman^6^

##### ^1^Psychiatry, Massachusetts General Hospital, Chelsea, MA, 2150, USA; ^2^Psychology, UCLA, Los Angeles, CA, 90095-1563, USA; ^3^Massachusetts General Hospital, Chelsea, MA, 02150, USA; ^4^Wang Ambulatory Care Center, Boston, MA, 02114-3117, USA; ^5^Psychology, Ryerson University, Toronto, ON, MBC 1B5, Canada; ^6^Dissemination and Training Division, National Center for PTSD and Stanford University, Menlo Park, CA, 94025, USA

###### **Correspondence:** Shannon Wiltsey Stirman

**Background**

In community settings, evidence-based treatments (EBTs) often face implementation challenges. One such challenge is fidelity to the treatment protocol; yet, fidelity alone is not sufficient to understand the effects of changes made during treatment implementation. Recent research has focused on systematically identifying modifications providers make when delivering EBTs. The present study aimed to utilize this coding framework to characterize provider modifications to an EBT for PTSD (Cognitive Processing Therapy; CPT) in a community health setting.

**Methods**

Using a modification coding framework, we rated audio-recorded CPT therapy sessions for modifications to the treatment protocol. After achieving reliability, coders rated modifications as absent or present, and described the nature of these modifications (e.g., removing/skipping elements).

**Findings**

Participants included providers (n = 27), who were 78.1% female, 76.9% white, and 12.3% Hispanic. These providers treated clients (n = 77), who were 71.3% female, 31.9% white, and 64.9% Hispanic. Across 174 sessions, 1012 modifications were identified. The most common modification was tailoring/tweaking/refining (n = 254, 25%), followed by loosening the session structure (n = 174, 17%) and shortening time spent during therapy visit (n = 111, 11%). These results differed significantly from adaptations found in a Canadian, English-speaking community mental health sample (n = 15 therapists, n = 32 clients).

**Implications for D&I Research**

Modifications appear to differ across populations. They might often increase provider satisfaction (i.e., acceptability) with an intervention, which may increase sustainability of CPT and improve patient outcomes. Additional research is needed to understand how EBTs are modified to fit clients’ immediate needs, and how they influence long-term implementation and client outcomes.

**Primary Funding Source**

National Institutes of Health

### S89 Differential cultural adaptation designs: a relevant methodological approach to empirically test the differential implementation feasibility and efficacy of cultural adapted interventions

#### Ruben Parra-Cardona

##### Department of Human Development and Family Studies, Michigan State University, East Lansing, MI, 48824, USA

**Background**

The cultural adaptation of evidence-based parenting interventions constitutes a promising alternative to reduce mental health disparities in the US. Implementation scholars have also emphasized the need to integrate implementation science and cultural adaptation studies. In this study, we aimed to examine whether a culturally-enhanced adapted parenting intervention with culture-specific sessions, had a significantly higher effect on feasibility and efficacy outcomes, compared to a culturally adapted intervention focused exclusively on parenting components.

**Methods**

This NIMH-funded investigation compared and contrasted the impact of two differentially culturally adapted versions of the evidence-based parenting intervention known as Parent Management Training, the Oregon Model (PMTO^TM^). Participants were allocated to one of three conditions: (a) a culturally adapted version of PMTO (only included PMTO core components), (b) a culturally-enhanced version of PMTO (core PMTO components and culturally-focused themes were included in this intervention), and (c) a wait-list control condition. Measurements were implemented at baseline (T1), treatment completion (T2) and 6-month follow up (T3). Initial efficacy of the adapted interventions was examined by analyzing quantitative outcome data from 190 parents. A multilevel modeling approach was utilized to analyze parenting (i.e., quality of parenting skills) and child outcomes (i.e. children’s externalizing and internalizing behaviors).

**Findings**

Findings indicate high implementation feasibility of both interventions, with an overall 86% retention rate of families, including 84% of fathers. Multilevel modeling findings indicated contrasting findings with regards to initial efficacy. Specifically, whereas parents in both adapted interventions showed statistically significant improvements on their quality of parenting skills when compared to parents in the wait-list control condition, only mothers in the culturally-enhanced intervention had statistically significant improvements on children’s internalizing symptoms when compared to the two alternative intervention conditions. Similarly, only fathers allocated to the culturally-enhanced intervention had statistically significant reductions on children internalizing and externalizing symptomatology when compared to the original adapted intervention and the wait-list control condition.

**Implications for D&I Research**

Data illustrate the benefits of implementing differential cultural adaptation designs. Furthermore, contrasting findings according to level of adaptation, indicates possibilities for relevant lines of research focused on integrating cultural adaptation and implementation science.

**Primary Funding Source**

National Institutes of Health

### S90 Implementation and dissemination of the Sikh American families oral health promotion program

#### Mary Northridge^1^, Rucha Kavathe^2^, Jennifer Zanowiak^3^, Laura Wyatt^3^, Hardayal Singh^4^, Nadia Islam^3^

##### ^1^Epidemiology & Health Promotion, New York University College of Dentistry and College of Global Public Health, New York, NY, 10010, USA; ^2^Community Health Projects, UNITED SIKHS, New York, NY, 10036, USA; ^3^Population Health, NYU School of Medicine, New York, NY, 10016, USA; ^4^Director, UNITED SIKHS, New York, NY, 10036, USA

###### **Correspondence:** Mary Northridge

**Background**

The *Sikh American Families Oral Health Promotion Program* used a community-based participatory approach to develop, implement, evaluate, and disseminate a culturally-tailored oral health/healthy living curriculum for the Sikh—South Asian community. Here we examine the impact of community engagement throughout the process of program implementation in five Gurdwaras (places of worship) in New York and New Jersey, and dissemination of the findings through targeted venues and the curriculum via e-Health resources.

**Methods**

An interactive curriculum was developed (consisting of four core and three special topics) based upon a community-led needs assessment, adaptation of evidence-based oral health curricula, guidance from professional dental and medical associations, and input from Community Advisory Board (CAB) members. The Consolidated Framework for Implementation Research guided a mixed methods evaluation, consisting of both process and outcome measures.

**Findings**

Five trained community educators delivered a total of 42 educational sessions. Improved oral hygiene behaviors and self-efficacy were found among program participants. For participants with no dental insurance prior to program enrollment (n = 58), 81.0% credited the program with helping them obtain insurance for themselves or their children. Further, for participants with no dentist prior to program enrollment (n = 68), 92.6% credited the program with helping them or their children find a local dentist. Short videos in Punjabi were created in response to feedback received from community educators and CAB members to reach men, especially.

**Implications for D&I Research**

Community engagement was key to successful program implementation and dissemination, from the implementation leaders (community educators) to the opinion leaders and champions (CAB members). Demonstrations of brushing and flossing techniques delivered by trusted community educators in familiar settings may be effective in promoting oral health for individuals, families, and communities. The expansion of Medicaid under the Affordable Care Act provided a mechanism for Sikh—South Asian program participants to obtain dental insurance for themselves and their children, and program resources helped link families to local dentists who accepted their dental insurance. Integration of community-based participatory research and implementation science approaches may prove effective in translating evidence-based practices into culturally-tailored programs that are delivered by trusted community leaders in local settings.

**Primary Funding Source**

Dr. Northridge was partially supported by NIDCR and OBSSR of NIH for the project titled, Integrating Social and Systems Science Approaches to Promote Oral Health Equity (grant R01-DE023072).

### S91 Mapping the social context of food procurement: identifying leverage points for disseminating healthy eating messages among a low-income population

#### Madalena Monteban^1^, Darcy Freedman^1,2^, Kimberly Bess^3^, Colleen Walsh^4^, Kristen Matlack^2^, Susan Flocke^1^, Heather Baily^5^

##### ^1^Epidemiology and Biostatistics, Case Western Reserve University, Cleveland, OH, 44106, USA; ^2^Prevention Research Center for Healthy Neighborhoods, Case Western Reserve University, Cleveland, OH, 44106, USA; ^3^Human and Organizational Development, Vanderbilt University, Nashville, TN, 37203, USA; ^4^Health Sciences, Cleveland State University, Cleveland, OH, 44115, USA; ^5^Anthropology, Case Western Reserve University, Cleveland, OH, 44116, USA

###### **Correspondence:** Madalena Monteban

**Background**

Diet-related behaviors are structured by both the physical and social food environment. Within the social food environment, social capital - resources, support, and information attained through social connections or ties - has important implications for health behaviors and outcomes. Little is known about the nature of social connections at food procurement places among low-income populations. Our analysis focused on social connections supporting food procurement behaviors among parents/caregivers receiving Supplemental Nutrition Assistance Program (SNAP) benefits.

**Methods**

A mixed-methods approach was used including participatory social network mapping and semi-structured interviews conducted with 30 parents/caregivers receiving SNAP in Cleveland, Ohio in 2015-16. Data collection focused on food procurement places and staff with whom participants hold a social connection. Two-mode social network analysis was used to examine the ties between procurement places and participants and core-periphery analysis to identify the most common food procurement places among the sample.

**Findings**

In all, 27 types of food procurement places were identified by the 30 participants. Ten of these places were central indicating they are most frequented by participants. Most participants (70%) held a social connection with a staff person at one or more procurement place. Convenience stores and food pantries were the most socially connected food procurement places with 60% and 55% of participants, respectively, holding a relationship with staff in these venues while none of the participants held a connection with farmer’s market staff. Qualitative analysis focused on three types of social connections: purely social, information exchange, and material benefit. Purely social connections included social interactions not related to food procurement. Information exchange included identifying deals at the store or indicating how to prepare a new food item. A few social connections offered material benefits in the form of setting aside sale items or price reductions for repeat customers.

**Implications for D&I Research**

Findings highlight that social connections with staff at food procurement places may be leveraged to disseminate healthy eating messages among SNAP recipients. Changes to the physical food environment may yield limited benefit without implementation of complementary interventions to either create new or catalyze existing social capital within these food procurement spaces.

**Primary Funding Source**

Centers for Disease Control and Prevention

### S92 Building system-wide capacity to reach those in need of intervention: a collaborative partnership approach to understanding uptake and sustainability of physical activity promotion in a statewide and national system

#### Samantha Harden^1^, NithyaPriya Ramalingam^2^, VCE Physical Activity Leadership Team^3^

##### ^1^Human Nutrition Foods and Exercise, Virginia Polytechnic Institute, Blacksburg, VA, 24060, USA; ^2^Translational Biology, Medicine, and Health, Virginia Polytechnic Institute, Blacksburg, VA, 24060, USA; ^3^ Virginia Cooperative Extension, Blacksburg, VA, 24060, USA

###### **Correspondence:** Samantha Harden

**Background**

Cooperative Extension (CE), which is available in every state and territory in the U.S., is a trusted source for education on food, agriculture, and youth development in the communities they serve. CE programming reaches a variety of underserved populations including, but not limited to, rural residents, older adults, families from low-income households, and immigrants. In 2014, physical activity was added as an objective to the CE strategic plan. Therefore, the system is now primed to translate the broad reach and impact CE professionals have had in agriculture to disseminate and implement physical activity promotion programs.

**Methods**

A collaborative partnership—defined as those in which researchers and community stakeholders work together on projects designed, initiated, and managed by the research team—was developed to train, support, and evaluate physical activity efforts in the state of Virginia. The partnership is founded on the meta-volition model constructs that 1) behavior change positively impacts the organization and 2) the shift from focusing on individual-level targets (self-efficacy, effectiveness) to population-level shifts (i.e., meta-volition).

**Findings**

Two years after the development of the partnership, there are eight active community health educators who represent both Family and Consumer Sciences and 4-H. Members are predominantly female (87%) and Caucasian (75%). Two physical activity promotion programs were chosen, adapted, and delivered across the state: 1) a statewide walking and fruit/vegetable consumption program and 2) a strength-training program for older adults. A strategic planning meeting will occur in August of 2016 to continue to guide structure, goals, and feedback among members of the partnership.

**Implications for D&I Research**

Identification of organizational culture, needs, and resources is imperative to ensure program uptake and sustainability. Building a collaborative partnership allows community health workers to contribute to the development and design of interventions that may be tailored for the audiences the serve; without the added time and resource burden of leading the efforts. Future research is needed on the collective impact the partnership and its efforts will have on population-level physical activity changes.

### S93 Using formative evaluation to improve the quality of a support system for healthy community coalitions

#### Kassy Alia^1^, Jonathan Scaccia^2^, Victoria Scott^3^, Rohit Ramaswamy^4^, Abraham Wandersman^1^

##### ^1^Psychology, University of South Carolina, Columbia, SC, 29208, USA; ^2^Institute for Healthcare Improvement, Reading, PA, 19606, USA; ^3^Psychology, University of North Carolina-Charlotte, Charlotte, NC, 28223, USA; ^4^Gillings School of Public Health, University of North Carolina, Chapel Hill, NC, 27599, USA

###### **Correspondence:** Kassy Alia

**Background**

There is an increasing interest in capacity-building interventions for improving community health. In the Interactive Systems Framework for Dissemination and Implementation, the support system is primarily responsible for building capacity in the delivery system (e.g., hospitals, FQHCs, community coalitions) using strategies such as tools, training, technical assistance (TA) and quality improvement. Formative evaluation of capacity-building interventions can help determine the effectiveness of support strategies so that they can be more efficient. In this presentation, we present support system results from the formative evaluation of a multi-site, national initiative designed to build community coalitions’ capacity to apply quality improvement principles to community health.

**Methods**

A mixed method, multi-informant approach was used to assess quality implementation (i.e. responsiveness, dosage, fidelity, adaptation, etc.) of support system activities. Funded by the Robert Wood Johnson Foundation to the Institute for Healthcare Improvement (IHI), twenty U.S. community coalitions were selected to participate based on levels of readiness for conducting community health improvement work. The support system included bi-annual trainings (Community Health Improvement Leadership Academies (CHILAs)), regular coaching from quality improvement specialists, regular monthly webinars, and regular meetings with peer groups (groups of peer communities matched with a community identified as having high readiness called “mentors”).

**Findings**

The majority of communities (77.3%) reported that the CHILA events and specific tools (e.g. “Switch Thinking”) were very useful for developing community health plans to structure their improvement projects. Individual coaching was also rated highly, with over half (54.6%) of communities indicating that coaching support was very useful for developing plans. Only 18.2% of communities rated mentor communities as very useful. In terms of their community health plans, the majority (65.2%) had completed aims/driver diagrams (i.e. their theory of what changes will lead to an improvement) in comparison to 17.3% who had completed plans to measure the results of their tests of change. Examples for how evaluation findings were used by the support system to improve implementation of capacity-building strategies will be shared.

**Implications for D&I Research**

We demonstrate how a formative evaluation approach may be used to strengthen the support system by providing ongoing feedback for improving delivery of capacity-building activities.

**Primary Funding Source**

The Robert Wood Johnson Foundation - This project is funded in a Robert Wood Johnson Foundation grant to the Institute for Healthcare Improvement

### S94 Designing EHR tools for collecting, summarizing, and acting on patient-reported social determinants of health data, in community health centers: results of a stakeholder-driven process

#### Rachel Gold^1,4^, Erika Cottrell^2^, Celine Hollombe^1^, Katie Dambrun^4^, Arwen Bunce^1^, Mary Middendorf^3^, Marla Dearing^4^, Stuart Cowburn^4^, Ned Mossman^4^, Gerry Melgar^5^

##### ^1^Science Program, Kaiser Permanente Center for Health Research, Portland, OR, 97227, USA; ^2^Family Medicine, Oregon Health and Science University, Portland, OR, 97202, USA; ^3^Epic, OCHIN, Inc., Portland, OR, 97201, USA; ^4^Practice Based Research Network, OCHIN, Inc., Portland, OR, 97207, USA; ^5^Medicine, Cowlitz Family Health Center, Long View, WA, 98632, USA

###### **Correspondence:** Rachel Gold

**Background**

Social determinants of health (SDH) are environmental / social factors that influence patients’ ability to act on recommended care, and thus directly impact the implementation of healthcare interventions and care guidelines. Collecting and presenting patients’ SDH data in electronic health records (EHRs) could help community health center (CHC) teams systematically identify and attempt to address SDH-related barriers to the delivery of guideline-concordant care. As approaches to capturing and presenting SDH data in CHCs’ EHRs have rarely been developed or tested, we developed EHR tools for collecting, presenting and acting on patient-reported SDH data in CHCs.

**Methods**

We built on the Institute of Medicine’s recommendations for collecting SDH into EHRs, and a protocol for asking SDH questions developed by a national CHC coalition. We then partnered with stakeholders from a national network of CHCs to design SDH-related EHR tools, via a six-month, iterative design process. Stakeholders included CHC staff (Community Health Workers, Behavioral Health Specialists, Social Workers, Care Coordinators, Referral Staff, Medical Assistants, Nurses, Primary Care Providers) and clinicians from the CHC network’s Clinical Operations Research Committee (total n ≈ 40 CHC staff).

**Findings**

We developed four EHR-based SDH data tools. The tools were integrated into existing EHR structures, and designed to enable care teams to collect and summarize patient-reported SDH data, act on identified SDH needs, and track referrals made to address these needs. We will describe the process used to develop these SDH data tools (data collection flowsheets, a summary report, social service referral preference lists, and a referral tracker) and present preliminary results on their adoption between 7/1/16-12/1/16; weekly data collection is underway.

**Implications for D&I Research**

Systematic identification of the SDH that impact primary care patients’ health, and automated methods for care teams to act on these SDH, could mitigate an important barrier to the implementation and dissemination of proven interventions and care guidelines. We will describe the process used to develop these tools, and present the tools themselves, to inform others seeking to address SDH-related barriers to implementation of diverse healthcare innovations in CHCs.

**Primary Funding Source**

National Institutes of Health - National Institute of Diabetes and Digestive and Kidney Diseases, 1R18HL095481-01

### S95 Feasibility of implementing a community clinic based interactive health kiosk about HPV vaccination targeting African American young adult women attending Planned Parenthood

#### Suellen Hopfer^1^, Michael Hecht^2^, Anne Ray^3^, Michelle Miller-Day^4^, Rhonda BeLue^5^, Greg Zimet^6^

##### ^1^Public Health, University of California, Irvine (UCI), Irvine, CA, 92697, USA; ^2^Communication, REAL Prevention LLC, Clifton, NJ, 07013, USA; ^3^Human Development, REAL Prevention LLC, New Jersey, NJ, 07013, USA; ^4^Communication Studies, Chapman University, Orange, CA, 92866, USA; ^5^Health Policy and Administration, Pennsylvania State University, University Park, PA, 16820, USA; ^6^Pediatrics, IUPUI, Indianapolis, IN, 46202, USA

###### **Correspondence:** Suellen Hopfer

**Background**

This study aimed to adapt a research tested intervention program (RTIP) promoting HPV vaccination for a community clinic setting (Planned Parenthood; PP) targeting African-American women ages 18-26. The intervention, Women’s Stories: The HPV Project (WS), was a narrative video intervention embedded within an interactive health kiosk to be used for waiting or exam rooms that aimed to increase HPV vaccine uptake in the target population.

**Methods**

Key informant interviews (*N* = 26) were conducted to identify culturally grounded vaccine decision narratives to inform video scripts. Participants (*N* = 12) offered feedback on scripts and adjustments were made prior to video production. After the kiosk was built, a usability study was conducted consisting of: (a) use of the kiosk by participants from the clinic population (*N* = 16) who provided feedback and (b) a waiting room observational study to see how patients interacted with the available health kiosk unprompted.

**Findings**

Four scripts were developed that emerged from interviews: general HPV information, doctor-patient interactions, a conversation between female peers on consequences of HPV, and male-female dialogue on male HPV transmission. Pilot testing revealed women found the scripts to be realistic, practical, and helpful. Edits to script language were made to be consistent with the target population vernacular. In part A (the usability study), participants rated the kiosk videos as engaging and interesting, with overall positive feedback about the health kiosk. During part B (waiting room observational study), no women used the kiosk in the waiting room unless prompted by PP staff.

**Implications for D&I Research**

Although WS was developed in collaboration with the target audience and well-received when use of the kiosk was directed, results from the observational study suggested placement in the waiting room would not result in use of the intervention. This critical information led to discussion with PP staff and determination that WS needs to be better incorporated into PP procedures. Next steps include a large scale randomized control trial in which we will compare two modes of delivery: incorporating WS into tablet-based check-in procedures vs. tablet-based intake conducted in the exam rooms (both compared to control).

**Primary Funding Source**

National Institutes of Health - R43CA192437

### S96 Telehospice 2.0: Implementation lessons from rural hospice care with mobile tablets

#### Eve-Lynn Nelson^1^, Sandy Kuhlman^2^, Gary Doolittle^3^, Hope Krebill^4^, Ashley Spaulding^4^

##### ^1^University of Kansas Medical Center, KU Center for Telemedicine & Telehealth, Fairway, KS, 66205, USA; ^2^Hospice Services and Palliative Care of Northwest Kansas, Inc., Phillipsburg, KS, 67661, USA; ^3^University of Kansas Cancer Center, Westwood, KS, 66205, USA; ^4^Midwest Cancer Alliance, Fairway, KS, 66205, USA

###### **Correspondence:** Eve-Lynn Nelson

**Background**

In underserved rural communities, hospice personnel travel great distances to reach patients, resulting in unique challenges to maintain access, quality, cost-effectiveness, and safety. To address these geographic disparities, the University of Kansas Medical Center piloted the country’s first telehospice (TH) service in 1998. A number of implementation barriers limited widespread adoption, including technology limitations, attitudes towards technology, and costs/reimbursement. An updated academic-community project utilizes secure mobile videoconferencing to support TH implementation in Kansas’ rural/frontier communities.

**Methods**

Leveraging lessons learned from this early work, a secure cloud-based videoconferencing solution was chosen for ease of use. To maximize limited resources, the selection of hospice partners was guided by Gustafson’s Organizational Change Manager (OCM). The OCM also informed implementation gaps. Mixed methods evaluation, including cost, continues as more hospices join over the next year.

**Findings**

Across the first six months of the pilot (February-July 2016), 50 rural TH encounters have occurred, encompassing 331 attendees and reflecting 31,591 minutes of videoconferencing. The most frequent TH uses to date have been: 1. Administrative (e.g., connecting hospice staff across a 16 county region); 2. Professional-to-professional (e.g., connecting hospice nurses at the home to additional TH professionals); and 3. Family support (e.g., connecting adult children outside the community with loved ones). Initial use of videoconferencing for administrative purposes developed a comfort level in videoconferencing for clinical and family support purposes. For staff meetings alone, the rural hospices have saved approximately $2,500/month in travel, with TH staff noting increased morale driven by the increased team communication. Next steps include videoconferencing between palliative medicine and other specialists directly to the rural homes to enhance symptom management and care coordination, as well as bereavement services over videoconferencing.

**Implications for D&I Research**

Compared with early work, technology advances and a community-engaged approach have increased TH adoption. With decreasing budgets as well as rural hospice closures, innovative, cost-effective, and community-driven approaches such as TH are needed to decrease rural disparities. As dissemination occurs within national hospice organizations, continued research is needed to understand best fit within rural/frontier hospices, to inform future urban applications, and to address reimbursement barriers.

### S97 Tailored small-media message intervention versus physician letters for addressing colorectal cancer screening disparities within an integrated healthcare system: a comparative effectiveness study

#### Theodore Levin^1^, Michael Sanchez^2^, Molly Landau^3^, Patricia Escobar^2^

##### ^1^Gastroenterology, The Permanente Medical Group, Walnut Creek, CA, 94596, USA; ^2^Regional Health Education, The Permanente Medical Group, Oakland, CA, 94612, USA; ^3^Quality and Operations Support, The Permanente Medical Group, Oakland, CA, 94612, USA

###### **Correspondence:** Theodore Levin

**Background**

Colorectal cancer (CRC) is the third leading cause of cancer death in the U.S. About 90% of CRC are treatable, when detected early through routine screening. However, significant CRC screening disparities exist, especially among Hispanics in California. The purpose of this study was to examine the effectiveness of a tailored small-media message intervention compared with a physician letter usual care intervention for increasing CRC screening rates among a non-adherent Hispanic patient population, within an integrated healthcare system client reminder program.

**Methods**

Both client reminders and small media are evidence-based strategies recommended by the Community Preventive Services Task Force, The Community Guide. Tailored small-media client reminders were mailed to eligible patients (n = 21,690), and compared to a retrospective group of patients (n = 21,671) whom received the physician letter usual care intervention. Tailored small-media client reminders included 2 postcards, 1 fecal immunochemical test (FIT) kit with a fotonota, and an automated outreach phone call. The tailored small-media materials was developed in the fotonota-style to illustrate a patient dialogue and address health literacy from the original FIT kit outreach. These materials were tested with 42 Latino men and women, ages 50-75, Spanish-speaking and English-speaking, to help refine the end-product before disseminating broadly to patients. Both intervention groups were matched based on the same eligibility criteria for CRC screening, using Healthcare Effectiveness Data and Information Set (HEDIS) criteria. Differences in difference analysis was used to mitigate the average effect change over time between the two groups.

**Findings**

Preliminary results suggest there was a significant difference in FIT screening rates at follow-up assessment between the tailored small-media message intervention (70.6%) and the usual care physician letter intervention (66.9%) using a 2-sided test, the difference from 0 = .0306 with a p-value < 0.01.

**Implications for D&I Research**

A tailored small-media message intervention designed to increase CRC screening rates among Hispanic patients in an integrated healthcare system client reminder program was significantly effective compared to usual physician letter client reminders.

**Primary Funding Source**

Internal operating funds

## Poster Slam

### S98 Tailoring knowledge in evidence based medicine: incorporating patient values and preferences into knowledge tools

#### Nadia Minian^1^, Peter Selby^2,3,4,5,6^, Aliya Noormohamed^1^, Laurie Zawertailo^3,7^, Dolly Baliunas^3^, Norman Giesbrecht^8^, Bernard Le Foll^3,7^, Andriy Samokhvalov^3^

##### ^1^Addictions Medicine, Centre for Addiction and Mental Health, Toronto, ON, M5S 3E3, Canada; ^2^Nicotine Dependence Service, Centre for Addiction and Mental Health, Toronto, ON, M5T1P7, Canada; ^3^Addictions Program, Centre for Addiction and Mental Health, Toronto, ON, M5T1P7, Canada; ^4^Family and Community Medicine, University of Toronto, Toronto, ON, M5G1V7, Canada; ^5^Psychiatry, University of Toronto, Toronto, ON, M5T1R8, Canada; ^6^Dalla Lana School of Public Health, University of Toronto, Toronto, ON, M5T3M7, Canada; ^7^Pharmacology and Toxicology, University of Toronto, Toronto, ON, M5T1P7, Canada; ^8^Social, Prevention and Health Policy Research Department, Centre for Addiction and Mental Health, Toronto, ON, M5S 2S1, Canada

###### **Correspondence:** Nadia Minian

**Background**

Evidence based medicine (EBM) is the integration of best research evidence with clinical expertise and patient values. There are few methodologies on how to design evidence based programs and resources to include patient values. The latter is an important aspect of patient centred care, and is essential for patients to trust the recommendations and empower them as consumers to make informed choices. The challenge is to create a dynamic process that integrates the best research evidence with patient values to create recommendations that will be widely adopted by the population and will achieve the best outcomes. This is especially difficult for changing health behaviours such as helping smokers attempting to quit to simultaneously reduce or stop their consumption of alcohol.

**Methods**

We utilized the Canadian Institute of Cultural Affairs snow card consensus method (a process designed to enable all participants to listen to each other, identify important values and work toward a common resource) to design a program (along with resources) that integrates Screening, Brief Intervention and Referral to Treatment (SBIRT) for alcohol into an existing clinical program for smoking cessation, so that they would be both evidence informed as well as culturally sensitive/patient centered. The process consisted of five steps: 1. setting the stage; 2. brainstorming; 3. clustering ideas; 4. naming the cluster; 5. naming the resources.

**Findings**

Engagement event participants (n = 18) designed an evidence informed and community sensitive resource consisting of five sections: 1. immediate crisis supports; 2. internal/external supports; 3. self-awareness strategies; 4. strategies for success; and 5. risks of drinking and smoking. In April 2016, 221 Ontario primary care clinics started offering this resource to eligible patients. The adoption rates are much higher than those reported in other studies which offered evidence based (but not community sensitive) resources for reducing alcohol (15% vs, 3%).

**Implications for D&I Research**

EBM has a well-developed method to summarize existing knowledge through the development of guidelines, meta-analyses and systematic reviews. This model describes a method to include patient values with preliminary evidence that such adaptation increases the adoption of such resources.

**Primary Funding Source**

Canadian Cancer Society

### S99 "Meet them where they are at" improving knowledge translation of health economics research on substance use disorder, HIV and HCV for policy

#### Zachary Meisel^1^, Daniel Polsky^2^, Bruce Schackman^3^, Julia Mitchell^2^

##### ^1^Emergency Medicine, Perelman School of Medicine at the University of Pennsylvania, University of Pennsylvania, Philadelphia, PA, 19104, USA; ^2^Department of Health Economics, Leonard Davis Institute of Health Economics, University of Pennsylvania, Philadelphia, PA, 19104, USA; ^3^Department of Healthcare Policy and Research, Weill Cornell Medical College, New York, NY, 10065, USA

###### **Correspondence:** Zachary Meisel

**Background**

Economic evidence to inform substance use disorder (SUD), hepatitis C (HCV), and HIV treatment coverage and investment decisions has grown in importance in today’s rapidly evolving healthcare system. With expanded opportunities to access evidence-based treatment for SUD, HCV, and HIV through insurance coverage expansions, parity laws, and system reforms, targeted and informed dissemination of research evidence to decision makers is critical for implementation of best-practice policy.

**Methods**

As part of the research program embedded within the policy and dissemination core for a NIDA center of excellence on the health economics of SUD, HCV and HIV treatment, investigators conducted an in-depth assessment of research end-users focused on identifying the needs for economic research to make informed decisions for these conditions. Taking advantage of the center’s policy board, snowball sampling was used to enroll 18 informants. An interview guide, informed by Mitton’s knowledge transfer framework of evidence based policy, was designed and piloted to explore the barriers and facilitators to the adoption and use of economic research for the treatment of these conditions within the context of the evolving broader healthcare system. 18 semi-structured interviews of end-users (from sectors including government, integrated health systems, private health insurance, pharmaceutical industry, advocacy, and clinical care) were conducted by interviewers trained in qualitative research methods. Interviews were recorded, transcribed, and analyzed using qualitative analysis software (NVivo 11, QSR International).

**Findings**

7 nodes were identified, through iteration, to emerge from the interviews: 1) process for economic research to reach policy makers, 2) the impact of health reform, 3) cost effectiveness of various treatments, 4) terminology and challenges with framing results, 5) differences between various clinical conditions, 6) structural barriers to knowledge transfer and use, 7) structural facilitators to knowledge transfer and use. Investigators have deemed the interviews to have reached near thematic saturation. Additional interviews and full thematic analyses are underway and will be completed by Sept 2016.

**Implications for D&I Research**

Producers and users of health economics research can engage in specific “push” and “pull” activities to respectively translate and use research evidence to improve policies related to the treatment of SUD, HCV and HIV.

**Primary Funding Source**

National Institutes of Health - Funded by a National Institute of Drug Abuse Center of Excellence Grant (P30DA040500)

### S100 Orthoguidelines: innovative dissemination to incorporate evidence-based recommendations into the episode of care

#### Kaitlyn Sevarino

##### Research, Quality, and Scientific Affairs, American Academy of Orthopaedic Surgeons (AAOS), Rosemont, IL, 60118, USA

**Background**

In orthopaedics, mobile technologies were not being optimized to disseminate evidence-based initiatives. Clinical practice guidelines (CPGs) were only accessible as reports; recommendations could not be searched, and multiple guidelines could not be accessed concurrently. These large PDFs were not easily viewed on mobile devices or during the episode of care. An AHRQ grant funded project created a new dissemination strategy for CPGs that allows users to access the recommendations and evidence-based information in a quicker, more concise manner.

**Methods**

A new platform, OrthoGuidelines, was created. The AAOS membership was surveyed prior to and following the platform launch to gauge the receptiveness of the orthopaedic community to mobile technologies, as well as web and smart-phone use, CPG use, and perceptions of CPG utility. The platform was also evaluated for utility via surveying and focus group; improvements were made based on member, leadership, and resident feedback.

**Findings**

We developed OrthoGuidelines to house all AAOS quality products. Users can navigate all guideline recommendations by orthopaedic disease, strength of evidence, and stage of care. The platform is laid out to enable quick navigation, comprehension, and clinical use. Users can also search all AAOS recommendations, rationales, and appropriate use criteria with a single keyword search. There have been over 5,000 app installs and over 62,000 website hits since launching in March of 2015. The membership survey results showed improvement across multiple metrics. Respondents were more likely to find what they were looking for in CPGs (41.5% to 48.5%), more likely to have accessed a CPG via smart-phone app (33% to 16.1%), more likely to find smart-phone apps for CPGs useful (79.6% to 93.6%), and more likely to have accessed a CPG online within the last month (28.7% to 33.3%).

**Implications for D&I Research**

OrthoGuidelines enables quicker and more widespread dissemination while also offering data collection opportunities as to where, when, and how often the recommendations are being accessed. The survey results demonstrate a change in behavior among AAOS members after the implementation of a new dissemination strategy. The results suggest that dissemination and implementation strategies influence not only how often CPGs are accessed, but also how useful they are to AAOS members.

**Primary Funding Source**

Agency for Healthcare Research and Quality - Development and testing of the tool discussed were funded by AHRQ Grant # 1R18HS021954-01

### S101 Improving data quality across 3 sub-Saharan African countries using the Consolidated Framework for Implementation Research (CFIR): results from the African Health Initiative

#### Sarah Gimbel^1^, Moses Mwanza^2^, Marie Paul Nisingizwe^3^, Catherine Michel^4^, Lisa Hirschhorn^5^

##### ^1^Family Child Nursing, University of Washington, Seattle, WA, 98112, USA; Global Health, University of Washington, Seattle, WA, 98112, USA; Implementation Science, Health Alliance International, Seattle, WA, 98112, USA; ^2^Primary Health Care, Centre of Infectious Diseases in Zambia, Lusaka, 00000, Zambia; ^3^Monitoring and Evaluation, Partners in Health, Butaro, 0000, Rwanda; ^4^Mozambique, Health Alliance International, Maputo, 0000, Mozambique; ^5^Implementation and Improvement Science Platform, Ariadne Labs, Boston, MA, 02215, USA; Global Health and Social Medicine, Harvard Medical School, Boston, MA, 02115, USA

###### **Correspondence:** Sarah Gimbel

**Background**

High-quality data are critical to inform, monitor and manage health programs. Over the seven-year Doris Duke Charitable Foundation’s African Health Initiative, three of the five country Partnerships supported by the initiative introduced strategies to improve the quality and evaluation of routinely-collected data at the primary health care level, and stimulate its use in evidence-based decision-making.

**Methods**

Using the Consolidated Framework for Implementation Research (CFIR) as a guide, this paper 1) describes and categorizes data quality assessment and improvement activities of the Partnerships in three sub-Saharan African countries (Mozambique, Rwanda, Zambia), and 2) identifies core intervention components and implementation strategy adaptations introduced to improve data quality in each setting.

**Findings**

Across the three countries, data quality audits and mentorship/supportive supervision were identified as core intervention components. The projects were introduced in different contexts with unique data quality challenges and priorities so implemented activities were classified along a continuum from core to peripheral, reflecting the degree of applicability across the three settings. Activities that both assessed and improved data quality (including data quality audits, mentorship and supportive supervision, establishment and/or strengthening of electronic medical record systems), received higher ranking scores from respondents. These higher ranking activities were also identified as effectively engaging MOH partners to varying degrees in their design, data collection and analysis.

**Implications for D&I Research**

This study, which applied the CFIR in three sub-Saharan African settings, enabled extraction of cross-intervention learnings which can inform implementers working to improve health information systems in resource constrained environments as a critical step to increasing data utilization for decision making, and ultimately the effectiveness of health care delivery.

**Primary Funding Source**

DDCF African Health Initiative

### S102 Barriers and facilitators of adherence to a standardized handoff intervention according to different clinician types: findings from a hybrid effectiveness-implementation study of operating room to intensive care unit handoffs

#### Meghan Lane-Fall^1^, Rinad Beidas^2,3^, Laura Di Taranti^1^, Mahrukh Choudhary^1^, Della Thonduparambil^1^, Lee Fleisher^1,3^, Frances Barg^1,4^

##### ^1^Anesthesiology and Critical Care, Perelman School of Medicine, University of Pennsylvania, Philadelphia, PA, 19104-4865, USA; ^2^Psychiatry, University of Pennsylvania, Philadelphia, PA, 19104, USA; ^3^Leonard Davis Institute of Health Economics, University of Pennsylvania, Philadelphia, PA, 19104, USA; ^4^Family Medicine and Community Health, University of Pennsylvania, Philadelphia, PA, 19104, USA

###### **Correspondence:** Meghan Lane-Fall

**Background**

Hospitalized patients whose care is transferred from one team to another experience a “handoff”, during which patient information and accountability are transferred. Despite the Joint Commission’s assertion that handoffs should be standardized to decrease harm, standardized handoffs remain uncommon. We sought to understand facilitators and barriers to adhering to a standardized handoff for patients being transferred from a surgical operating room (OR) to the intensive care unit (ICU).

**Methods**

In 2014, as part of the Handoffs and Transitions in Critical Care (HATRICC) study, we conducted interviews, focus groups, and collected surveys to elicit barriers and facilitators of adhering to a standardized handoff process. We analyzed qualitative data according to grounded theory and characterized quantitative data with descriptive statistics.

**Findings**

We conducted 63 interviews, 3 focus groups (19 participants), and collected 133 surveys from clinicians participating in OR to ICU handoffs, including physicians, nurses, and advanced practitioners. All of these clinicians agreed that checklists or protocols could be useful for improving handoffs, but concern about the difficulty of achieving change was common. Surgeons and anesthesia clinicians identified checklist/protocol simplicity, relevance and flexibility as facilitators and excessive length as a barrier to checklist or protocol adherence. Nurses expressed skepticism that physicians would adhere to a protocol or checklist. For all clinicians, competing priorities were perceived to threaten adherence to a candidate protocol or checklist. For nurses, these priorities included direct patient care and monitoring. For OR physicians and nurse anesthetists, there was perceived time pressure to leave the handoff episode to care for other patients. When asked what type of data would prompt them to change their handoff practice, the greatest number of respondents selected evidence supporting improved outcomes with a different process (117/133, 88.0%), followed by data showing improved communication (95/133, 71.4%) and data showing that patients in our health system had been harmed during handoffs (84/133, 63.2%).

**Implications for D&I Research**

Different clinician types perceive distinct barriers and facilitators of adherence to standardized handoffs. Evidence linking handoffs to outcomes was most valued as a catalyst to spur change. Handoff process development should include different clinician perspectives and address clinicians’ desire for supporting evidence.

**Primary Funding Source**

Safety Scientist Career Development Award

### S103 The Standards for Reporting Implementation Studies (StaRI)

#### Paul Meissner^1^, Hilary Pinnock^2^, Melanie Barwick^3^, Christopher Carpenter^4^, Sandra Eldridge^5^, Gonzalo Grandes-Odriozola^6^, Chris Griffiths^5^, Jo Rycroft-Malone^7^, Elizabeth Murray^8^, Anita Patel^9^, Aziz Sheikh^2,10^, Stephanie JC Taylor^5^, Brian Mittman^11^, Martin Guilliford^12^, Gemma Pearce^13^

##### ^1^Family and Social Medicine, Montefiore Medical Center, Bronx, NY, 10467, USA; ^2^Asthma UK Centre for Applied Research, Allergy and Respiratory Research Group, Usher Institute of Population Health Sciences and Informatics, University of Edinburgh, Edinburgh, EH8 9AG, UK; ^3^Research Institute, The Hospital for Sick Children, Toronto, ON, M5G 1X8, Canada; ^4^Division of Emergency Medicine, Washington University, St. Louis, MO, 63110, USA; ^5^Centre for Primary Care and Public Health, Queen Mary University of London, London, E1 2AT, UK; ^6^Primary Care Research Unit of Bizkaia, Basque Health Service, Bilbao, Spain; ^7^ School of Healthcare Sciences, Bangor University, Bangor, Gwynedd, LL57 2EF, UK; ^8^ Research Department of Primary Care and Population Health, University College London, London, NW3 2PF, UK; ^9^ Centre for Primary Care and Public Health, Barts and The London School of Medicine and Dentistry, London, E1 2AT, UK; ^10^ Centre for Population Health Sciences, Brigham and Women's Hospital, Boston, MA, 02120-1613, USA; ^11^ Research and Evaluation, Kaiser Permanente Southern Calif / US Dept of Veterans Affairs, Pasadena, CA, 91101, USA; ^12^ Division of Health and Social Care Research, King's College London, London, WC2R 2LS, UK; ^13^ Centre for Technology Enabled Health Research, Coventry University, Coventry, CV1 5FB, UK

###### **Correspondence:** Paul Meissner

**Background**

Implementation studies are often poorly reported making it difficult to identify relevant studies and to synthesize the evidence base to inform practice.

**Methods**

A systematic literature review and international eDelphi exercise identified candidate items for inclusion in the StaRI reporting standards. A consensus workshop convened with15 international multidisciplinary delegates reviewed candidate items and produced a draft checklist. Discussions were informed by the outcome of the e-Delphi exercise, other published reporting standards, the wider literature, and the panel’s IS expertise. The initial draft statement and documents were subsequently discussed and developed iteratively by e-mail.

**Findings**

The StaRI checklist identifies standards for reporting implementation studies that evaluate implementation efforts to enhance the adoption and sustainability of evidence-based interventions across the range of study designs used in implementation science (IS). Two defining concepts underpin the StaRI reporting standards: (i) the dual strands of describing the implementation strategy and the clinical, healthcare, or public health intervention

being implemented; and (ii) unlike most reporting standards that apply to a specific research methodology applied in range of contexts, StaRI standards pertain to the broad range of study designs employed in implementation science. The presentation will review the 27checklist items.

**Implications for D&I Research**

Implementation science is a rapidly evolving field with no clear consensus on quality standards for reporting. The StaRI is an evolving document and potentially a catalyst for discussing and defining how implementation studies are planned and reported.

**Primary Funding Source**

Contributions from the Asthma UK Centre for Applied Research [AC-2012-01]; Chief Scientist Office, Scottish Government Health and Social Care Directorates [PCRCA_08_01]; the Centre for Primary Care and Public Health, Queen Mary University of London

### S104 Clinician-stakeholders’ perspectives on using patient portals to return genetic screening results

#### Diane Korngiebel^1^, Kathleen West^2^, Wylie Burke^3^

##### ^1^Biomedical Informatics and Medical Education, University of Washington, Seattle, WA, 98195, USA; ^2^Institute of Public Health Genetics, University of Washington, Seattle, WA, 98195, USA; ^3^Bioethics and Humanities, University of Washington, Seattle, WA, 98195, USA

###### **Correspondence:** Diane Korngiebel

**Background**

Genetic tests have traditionally been returned by genetic counselors who can explain their implications. Electronic Health Record (EHR) patient portals may increase the efficiency of results return, and could activate patient follow-up for conditions such as Lynch Syndrome (LS), an inherited colorectal cancer syndrome. Universal colorectal cancer tumor screening (UTS) for LS is recommended, but patients who screen positive and their at-risk relatives exhibit low uptake of genetic services. Stakeholder input is necessary to determine acceptability and appropriate implementation of patient portals to improve follow-up outcomes of UTS.

**Methods**

Twenty interviews were conducted with clinicians from six specialties. Participants were recruited using purposive and snowball sampling and represented urban and rural settings ranging from academic medical centers to local clinics and hospitals. Data were analyzed using directed content analysis and thematic analysis across content categories.

**Findings**

Genetic specialists felt that genetic-related testing (screen or sequencing), should not be returned electronically. Others (gastrointestinal clinicians, oncologists, pathologists, primary care providers) felt results could be returned through portals if supplemented by a conversation, because genetic results are “sensitive.” Several mentioned that routine screening information or a consent process would enable patients to anticipate the possibility of positive screen results. Some stated that patient preferences for communication modes should be respected, whether in-person, phone, or electronic, and that the timing of the return of results in the context of a cancer diagnosis was critical. Portal results should include tailored content including links to reputable information, an explanation of the limitations of a screen, the need for confirmatory genetic testing, and contact information for a genetics specialist or notice of a referral.

**Implications for D&I Research**

Often patient portals are designed without patient or clinician input. With precision medicine, opportunities increase for patients to manage their health information electronically and for clinicians and healthcare organizations to leverage patient-centered information technology. LS screening is a well-evidenced application of genomic medicine: using patient portals to communicate screening results can inform electronic return of results for other clinical implementations of genomic medicine. Next steps include exploring patient views, and evaluating their likelihood of acting based on results received through the patient portal.

**Primary Funding Source**

National Institutes of Health

### S105 Healthlinks: increasing small, low-wage worksites' implementation of evidence-based interventions

#### Peggy Hannon^1^, Jeffrey Harris^1^, Kristen Hammerback^1^, Marlana Kohn^1^, Gary KC Chan^2^, Riki Mafune^3^, Amanda Parrish^1^, Christian Helfrich^1,4,5^, Shirley Beresford^6^, K. Joanne Pike^7^

##### ^1^Department of Health Services, University of Washington, Seattle, WA, 98105, USA; ^2^Department of Biostatistics, School of Public Health, University of Washington, Seattle, WA, 98199, USA; ^3^Health Promotion Research Center, University of Washington, Seattle, WA, 98105, USA; ^4^Health Services Research and Development, VA Puget Sound Health Care System, Seattle, WA, 98101, USA; ^5^VA Health Services Center of Innovation for Veteran-Centered and Value-Driven Care, Department of Veterans Affairs, Seattle, WA, 98108, USA; ^6^Epidemiology, University of Washington, Seattle, WA, 98195, USA; ^7^American Cancer Society, Preventive Health Partnership, Atlanta, GA, 30303, USA

###### **Correspondence:** Peggy Hannon

**Background**

The worksite is a powerful venue for reaching adults with evidence-based interventions (EBIs) to prevent chronic disease. Small worksites are less likely to have wellness programs with EBIs than larger worksites. The University of Washington and the American Cancer Society developed HealthLinks to disseminate EBIs to small worksites in low-wage industries. The core components of HealthLinks include an assessment of wellness EBIs, a tailored recommendations report, a set of toolkits for recommended EBIs, and monthly check-ins and technical assistance. We conducted a three-arm, site-randomized trial to test whether HealthLinks increased EBI adoption at small worksites, and whether having a wellness committee facilitated EBI adoption.

**Methods**

We recruited 78 worksites and retained 72 through the 15-month follow-up. Worksites were randomly assigned to one of three arms after completing baseline data collection: standard HealthLinks (n = 26), HealthLinks plus wellness committee (n = 25), or delayed control (n = 21). Worksites in the two HealthLinks arms received the HealthLinks intervention from a trained interventionist. The interventionist delivered HealthLinks to a key contact at the worksite, usually the human resources manager. In the HealthLinks plus wellness committee arm, worksites also received toolkits and interventionist support to create a wellness committee. The assessment of wellness EBIs was administered again at 15 months; these assessments are the source of the baseline and follow-up data. We scored worksites’ implementation of policy, program, and communication EBIs on a 0-100% scale.

**Findings**

EBI implementation increased in both HealthLinks arms compared to the delayed control arm, p < .001. Worksites in the standard HealthLinks arm increased from 17% at baseline to 49% at follow-up, worksites in the HealthLinks plus wellness committees arm increased from 19% at baseline to 48% at follow-up, and worksites in the delayed control arm had 20% EBI implementation at baseline and 23% at follow-up. The two HealthLinks arms did not differ in EBI implementation at baseline or follow-up.

**Implications for D&I Research**

Small worksites that participated in HealthLinks more than doubled their EBI implementation. Future HealthLinks research will focus on sustainability of EBI implementation after worksites complete HealthLinks, as well as work with the American Cancer Society to study different methods of taking HealthLinks to scale.

**Primary Funding Source**

National Institutes of Health - R01 funded by the National Cancer Institute

### S106 A qualitative investigation of a nationally disseminated, evidence-based Lay Health Advisor (LHA) Program for African American women: advancing understanding of the implementation and sustainability of LHA programs in community settings

#### Rachel Shelton^1^, Lina Jandorf^2^, Deborah Erwin^3^, Thana-Ashley Charles^1^

##### ^1^Department of Sociomedical Sciences, Columbia's Mailman School of Public Health, New York, NY, 10032, USA; ^2^Oncological Sciences, Mount Sinai School of Medicine, New York, NY, 10029, USA; ^3^Division of Cancer Prevention and Population Sciences, Roswell Park Cancer Institute, Buffalo, NY, 14263, USA

###### **Correspondence:** Rachel Shelton

**Background**

Lay Health Advisor (LHA) programs have made strong contributions towards the elimination of health disparities, and are increasingly being implemented to promote health and prevent disease. Developed in collaboration with African American survivors, the National Witness Project (NWP) is an evidence-based, community-led LHA program that improves cancer screening among African American women. Over the past twenty years, NWP has been successfully disseminated, replicated, and implemented nationally in over 40 sites in 22 states in diverse community settings, reaching over 15,000 women annually. We sought to advance understanding of barriers and facilitators to the implementation and sustainability of LHA programs in community settings from the viewpoint of the LHAs, as well as the broader impact of the program on African American communities and LHAs.

**Methods**

In-depth telephone interviews were conducted among 76 African-American LHAs at eight NWP sites at baseline and 12-18 months later, between 2010 and 2013. Transcripts were analyzed and coded independently by two coders using Dedoose software for qualitative analysis. A thematic content analysis was conducted to identify key themes and illustrative quotes.

**Findings**

Qualitative data provides insight into inner and outer contextual factors (e.g., community and organizational partnerships, site leadership, program champions, funding), implementation processes (e.g., initial and ongoing training), as well as characteristics of the intervention (e.g., perceived need and fit in African American community) and characteristics of LHAs (e.g., motivations, burnout) that are perceived to impact the continued implementation and sustainability of NWP.

**Implications for D&I Research**

We highlight the implications of findings to: 1) refine theoretical frameworks in Dissemination & Implementation Science; and 2) inform strategies to support the continued implementation and sustainability of evidence-based LHA interventions in community settings. In addition, building off of these qualitative findings and our prior quantitative research from this study, we present the Lay Health Advisor Sustainability Framework, a multi-level framework proposed for researchers to study the long-term implementation and sustainability of LHA programs in community settings.

**Primary Funding Source**

National Institutes of Health - R03 study funded by the National Cancer Institute

### S107 De-implementing prescription of opioid medication for chronic pain in rural health clinics

#### Michael Parchman^1^, Laura-Mae Baldwin^2^, Brooke Ike^2^

##### ^1^MacColl Center for Health Care Innovation, Group Health Research Institute, Seattle, WA, 98101, USA; ^2^Department of Family Medicine, University of Washington, Institute of Translational Health Sciences, Seattle, WA, 98195, USA

###### **Correspondence:** Michael Parchman

**Background**

Prescription opioid abuse, overdose and death are epidemic in rural settings. Addressing this epidemic requires “de-implementation” of the longstanding strategy of prescribing opioids for chronic pain. Asking providers and patients to reduce or eliminate an existing treatment is a challenge. The Team-Based Opioid Management Project developed a “de-implementation framework” of Six Building Blocks to safer opioid prescribing to create a structure for rural clinic organizations in changing their opioid prescription practices. We describe the Six Building Blocks, and how organizations’ self-assessment of their performance in these six domains informed their de-implementation strategies.

**Methods**

The Building Blocks were based on opioid prescription management system changes identified during visits to thirty exemplar U.S. primary care clinics that had implemented team-based workforce innovations. We developed a 20-item survey to assess organizations on the Building Blocks; asked providers, staff and administrators from the six organizations to complete the survey at baseline; and calculated survey measure frequencies by organization.

**Findings**

The Six Building Blocks include: 1) leadership support, 2) registry use to proactively manage patients, 3) revision of policies, treatment agreements, and workflows, 4) patient-centered visits, 5) resources and support for complex patients, and 6) measuring and monitoring success. In two thirds or more of the organizations, either leaders or clinical staff ranked their sites as most challenged in: 1) using data for care improvement, 2) formal policies and standard work related to opioid prescribing and prescription monitoring program use, 3) patient education materials, 4) patient involvement in decision-making, 5) care plans for chronic pain management, and 6) behavioral health services. All organizations began de-implementation by revising their opioid management policies, workflows, and treatment agreements. Next, organizations simultaneously began planning to use data for care improvement, to educate patients about opioid risks and clinic efforts to reduce these, and to integrate care plans into patient visits. Improving behavioral health services was a key gap but a hurdle too big for early stages of de-implementation.

**Implications for D&I Research**

A structured de-implementation framework with a self-assessment survey helped clinic organizations prioritize system changes as they worked to reduce opioid prescribing for chronic pain.

**Primary Funding Source**

Agency for Healthcare Research and Quality

### S108 Improving methods for implementing computer-based mapping in VA home-based primary care practices nation-wide

#### Jacqueline Fickel^1^, Jason Lind^2^, Diane Cowper^3^, Marguerite Fleming^4^, Amy Sadler^5^, Melinda Dye^6^, Judith Katzburg^7^, Michael Ong^8^, Sarah Tubbesing^9^

##### ^1^Center for the Study of Healthcare Innovation, Implementation & Policy, VA Greater Los Angeles Healthcare System, Sepulveda, CA, 91343, USA; ^2^HSR&D, Dept. of Veterans Affairs, Tampa, FL, 33637, USA; ^3^Department of Veterans Affairs, Gainesville, FL, 32608, USA; ^4^OIA, VHA, Washington, DC, 20240, USA; ^5^OIA, Department of Veterans Affairs, Kansas City, MO, 64105, USA; ^6^CHE, Dept. of Veterans Affairs, Sioux City, IA, 87108, USA; ^7^HSR&D, VHA, Los Angeles, CA, 91343, USA; ^8^HSR&D Center for the Study of Healthcare Innovation, Implementation, and Policy, VA Greater Los Angeles Healthcare System, Los Angeles, CA, 90073, USA; ^9^GEC, Dept. of Veterans Affairs, Los Angeles, CA, 90073, USA; David Geffen School of Medicine at UCLA, Los Angeles, CA, USA

###### **Correspondence:** Jacqueline Fickel

**Background**

This work develops and evaluates implementation of Geographic Information System (GIS) mapping for the Veterans Affairs Home-Based Primary Care (HBPC) program. HBPC provides comprehensive primary care to Veterans who have complex, chronic conditions and face significant barriers to accessing clinic-based services. HBPC practices can benefit from secure, shareable maps to improve efficiency of practice management. While initial pilot work showed GIS can be feasible and useful, further work was needed to develop implementation strategies that fit contexts of diverse local sites.

**Methods**

This quality improvement study uses several methods in combination to support local implementation. The multi-disciplinary coordination team works with selected HBPC sites to learn and use GIS mapping. Implementation facilitation is used to adapt activities to specific site needs. Evaluation combines quantitative, survey, and interview data to assess extent of adoption, facilitators and barriers. Formative feedback is used to refine a toolkit that includes training, technical support, and other materials. HBPC sites participate voluntarily. Selection included pre-implementation assessment of readiness. Participation spread from a single site in 2012 to 17 sites in 2015, including small, medium, and large practices, in various geographic areas nationwide.

**Findings**

It is feasible for local HBPC sites to learn and use GIS maps in practice management, given adequate staff skills, training, and time. By October 2015, 15 of the 17 sites were making maps, and using them in practice management; the other two sites were still training. The most common uses included assigning patients to providers, managing territories and day-to-day travel, program expansion, and emergency preparedness. Sites were generally satisfied, and perceived that the maps helped improve patient assignment, providers’ travel time, and number of visits to patients’ homes. They also reported challenges related to staff turnover in key mapping-related positions, as well as competing demands on time. The majority strongly recommended ongoing training and technical support, to hone their map-making skills and carry out more complex tasks.

**Implications for D&I Research**

The combination of implementation methods that could be adapted to specific needs of sites was an asset. Various organizational stakeholders at the national level will need to collaborate for further spread and ongoing sustainability in regular operations.

**Primary Funding Source**

Department of Veterans Affairs - Operational special project

### S109 Re-conceptualizing facilitation in the implementation of mission-vet in VA homeless services

#### Megan McCullough^1,2^, Molly Simmons^1,2^, Vera Yakovchenko^1,2^, Autumn Harnish^1^, Sonya Gabrielian^3,4^, Keith McInnes^1,2^, Jeffrey Smith^5^, David Smelson^1,6^

##### ^1^Center for Healthcare Organization and Implementation Research (CHOIR), VA Health Services Research & Development, Department of Veterans Affairs, Bedford, MA, 01730, USA; ^2^Department of Health Law, Policy and Management, Boston University School of Public Health, Boston, MA, 02118, USA; ^3^Department of Veterans Affairs, Los Angeles, CA, 90073, USA; ^4^Department of Psychiatry, UCLA, Los Angeles, CA, 90073, USA; ^5^VA Quality Enhancement Research Initiative (QUERI) for Team-Based Behavioral Health, North Little Rock, AR, 72114, USA; ^6^Department of Psychiatry, University of Massachusetts Medical School, Worcester, MA, 01655, USA

###### **Correspondence:** Megan McCullough

**Background**

Maintaining Independence and Sobriety through Systems Integration, Outreach, and Networking- (MISSION) is an evidence-based intervention that uses peer and case managers to engage homeless Veterans with co-occurring mental health and substance use disorders in care. MISSION is being implemented in VA Greater Los Angeles’ (GLA) patient-centered medical home for homeless Veterans (Homeless Patient Aligned Care Team, HPACT), comparing implementation as usual (IU) to implementation enhanced with Facilitation. Using a mixed-methods formative evaluation (FE) to identify perceived barriers to implementation, we developed an innovative Facilitation strategy to overcome organizational barriers.

**Methods**

We administered an organizational readiness to change (ORCA) and implementation climate survey to staff (N = 42) trained in MISSION. ORCA measures were analyzed and mapped to the Consolidated Framework for Implementation Research (CFIR) constructs. We also conducted a purposive sample (N = 14) of semi-structured qualitative interviews of staff receiving MISSION training. Deductive content analysis was guided by the CFIR and triangulated with quantitative findings.

**Findings**

Survey and interviews identified potential barriers and facilitators to integrating MISSION into practice. The most salient CFIR constructs were ‘outer setting’ and ‘characteristics of individuals’; staff felt responsible for improving patients’ treatment engagement. Staff believed there was leadership support for MISSION, but sought more guidance on implementation expectations. Survey data on ‘inner setting’ indicates it was not immediately amenable to change. When mapped with interview data, it emerged that HPACT case managers and peers had no organizational pathways to work with each other on MISSION since peers are not on GLA’s HPACT Teams. Consequently, we re-conceptualized our planned external + internal facilitation strategy. Typically, there is one Internal Facilitator (IF) per team; our re-designed enhancement of Facilitation will involve the external facilitator working with IFs from each stakeholder group to bridge workflow and organizational/disciplinary silos.

**Implications for D&I Research**

This project illustrates how FE can identify organizational barriers to adoption of complex clinical interventions and inform innovative adaptations to facilitation strategies to support implementation. This project will test this enhanced Facilitation approach, contributing substantively to a growing literature on facilitation’s value in supporting implementation of evidence-based practices in the presence of complex organizational barriers.

**Primary Funding Source**

Department of Veterans Affairs

### S110 Sustainment leadership predicts more positive attitudes towards evidence based practice over time

#### John Ferrand, Elisa Torres, Amy Green, Gregory Aarons

##### Psychiatry, UC San Diego, La Jolla, CA, 92093, USA

###### **Correspondence:** John Ferrand

**Background**

Leadership is crucial in shaping organizational climates and worker’s perceptions and responses to organizational change. Implementation science frameworks posit leadership as a key component in the implementation and sustainment of evidence-based practices (EBPs). Measures have recently been developed and validated to examine implementation leadership and implementation climate. These measures have also been modified to measure leadership and climate during the sustainment phase of the EPIS (Exploration, Preparation, Implementation, Sustainment) framework. This prospective study examines a mediational model wherein sustainment leadership and sustainment climate predict future attitudes towards EBP. We hypothesized that more positive sustainment leadership would be associated with more positive sustainment climate, which, in turn, would predict more positive attitudes towards EBPs.

**Methods**

Survey data were collected from 112 allied health service providers in a child welfare system in two states across a two year time period. Providers were nested in 28 teams within 21 organizations. Sustainment Leadership Scale and Sustainment Climate Scale scores in 2014 were used to predict Evidence-Based Practice Attitude Scale scores in 2015. Structural equation modeling (SEM), accounting for the nested data structure was used to examine the impact of sustainment leadership on sustainment climate and their associations with provider attitudes toward EBP. “Rmediation” analysis was used to examine indirect effects. A competing partial mediation model was also examined and compared to the hypothesized model using the Satorra-Bentler (S-B) chi-squared difference test.

**Findings**

Results provided support for positive relationships linking sustainment leadership to sustainment climate and attitudes toward EBPs. Significant positive relationships were found between sustainment leadership and sustainment climate (β = .63, *p* < .001), and between sustainment climate and provider attitudes toward EBP one year later (β = .56, *p* < .001). Results also supported the presence of a mediational relationship (indirect effect = .362, 95% CI [.169, .604]). Result of the S-B chi-squared difference test also supported full mediation (S-B χ^2^Δ = 1.71, *p* > .05).

**Implications for D&I Research**

Organizations implementing EBPs may benefit from improving leadership and climate for EBP implementation and sustainment. Training to improve sustainment leadership may improve the efficiency and effectiveness of implementation efforts in allied health organizations and to subsequently increase the public health impact of EBPs.

**Primary Funding Source**

National Institutes of Health - This study was supported by NIMH Grants R01MH072961

### S111 Extended follow-up of patient outcomes in the multi-center randomized cogent study of in-person versus telephone disclosure of cancer genetic test results

#### Angela R Bradbury^1^, Linda J. Patrick-Miller^2^, Brian L. Egleston^3^, Susan M. Domchek^4^, Olufunmilayo I Olopade^2^, Michael J Hall^5^, Mary B Daly^6^, Linda Fleisher^7^, Generosa Grana^8^, Pamela Ganschow^9^, Dominique Fetzer^4^, Amanda Brandt^4^, Rachelle Chambers^10^, Dana F. Clark^8^, Andrea Forman^6^, Rikki S Gaber^11^, Cassandra Gulden^2^, Janice Horte^12^, Jessica Long^4^, Terra Lucas^13^, Shreshtha Madaan^14^, Kristin Mattie^12^, Danielle McKenna^4^, Susan Montgomery^6^, Sarah Nielsen^2^, Jacquelyn Powers^4^, Kim Rainey^6^, Christina Rybak^6^, Christina Seelaus^13^, Jessica Stoll^2^, Jill Stopfer^4^, Xinxin (Shirley) Yao^12^, Michelle Savage^6^

##### ^1^Department of Medicine, Hematology/Oncology / Department of Medical Ethics and Health Policy, The University of Pennsylvania, Philadelphia, PA, 19104, USA; ^2^Center for Clinical Cancer Genetics, The University of Chicago, Chicago, IL, 60637, USA; ^3^Biostatistics and Bioinformatics Facility, The Fox Chase Cancer Center, Philadelphia, PA, 19111, USA; ^4^Department of Medicine, Division of Hematology-Oncology, The University of Pennsylvania, Abramson Cancer Center, Philadelphia, PA, 19104, USA; ^5^Clinical Genetics / Gastrointestinal Risk Assessment, The Fox Chase Cancer Center, Philadelphia, PA, 19111, USA; ^6^Department of Clinical Genetics, The Fox Chase Cancer Center, Philadelphia, PA, 19111, USA; ^7^Research Institute, The Children's Hospital of Philadelphia, Philadelphia, PA, 19104, USA; Population Science, Fox Chase Cancer Center, Philadelphia, PA, 19015, USA; ^8^Hematology and Medical Oncology, MD Anderson Cancer Center at Cooper, Camden, NJ, 08043, USA; ^9^Internal Medicine, John H. Stroger, Jr. Hospital, Chicago, IL, 60612, USA; ^10^Department of Medicine, The University of Chicago, Chicago, IL, 60637, USA; ^11^Breast and Cervical Cancer Screening Program, John H. Stroger, Jr. Hospital, Chicago, IL, 60612, USA; ^12^Cancer Genetics Program, MD Anderson Cancer Center at Cooper, Camden, NJ, 08043, USA; ^13^Cancer Screening Program, John H. Stroger, Jr. Hospital, Chicago, IL, 60612, USA; ^14^Comprehensive Cancer Risk and Prevention Clinic, The University of Chicago, Chicago, IL, 60637, USA

###### **Correspondence:** Angela R Bradbury

**Background**

Alternative delivery models are needed in the era of Precision Medicine given a shortage of genetic providers and increasing utilization of genetic testing across medicine. Telephone disclosure (TD) of genetic test results, including multi-gene panel testing, is non-inferior to usual care in-person disclosure (IPD) for short-term distress but failed non-inferiority for knowledge and longitudinal data is needed.

**Methods**

970 patients at 5 centers undergoing clinical cancer genetic testing were randomly assigned to usual care IPD (n = 497) or TD (n = 473) of results in the COGENT Study (NCT01736345). Participants completed surveys at baseline, post-disclosure and at 6 months. Primary outcomes were knowledge, state and general anxiety. We used non-inferiority tests for primary analyses, and T-tests and logistic regressions for secondary analyses.

**Findings**

TD was not worse than IPD for general and state anxiety both post-disclosure and at 6 months, but did not reach the non-inferiority threshold for knowledge at either time point. In secondary analyses, there were no significant differences in anxiety, depression, or cancer worry between arms, but a trend towards lower knowledge gain in the TD arm (-0.40 v. +0.08 in IPD, p = 0.07). Among those disclosed by TD with a genetic counselor, 195 (50%) returned for the recommended clinical follow-up with a physician to discuss medical management. Not returning for follow-up after TD varied by site and was associated with a negative result, *BRCA1/2* testing only, being male and non-white. Those who did not return for follow-up had significantly less gain in knowledge at 6 months (-0.23) compared to those who had TD and returned for follow-up (+0.36, p = 0.05). Those who had TD and returned for follow-up did not have significant differences in change in knowledge compared to IPD.

**Implications for D&I Research**

Patient reported distress is not unacceptably worse with TD of results than IPD, but knowledge failed the test for non-inferiority. Knowledge gains were significantly lower for those who did not return for medical follow-up. Telephone disclosure of genetic test results, even MGPT, may be a reasonable alternative to in-person disclosure for patients who agree to return to meet with a provider for medical management recommendations.

**Primary Funding Source**

National Institutes of Health - NIH R01 CA160847

### S112 The Participant-Reported Implementation Update and Score (PRIUS): a new method for capturing implementation-related developments over time

#### Edward Miech^1^, Teresa Damush^2^, Nicholas Rattray^3^, Jennifer Myers^3^, Barbara Homoya^3^

##### ^1^HSR&D, Richard L. Roudebush VA Medical Center, Indianapolis, IN, 46202, USA; ^2^HSR&D, VA Stroke Queri Center, Indianapolis, IN, 46239, USA; ^3^HSR&D/QUERI, Roudebush VA Medical Center, Indianapolis, IN, 46202, USA

###### **Correspondence:** Edward Miech

**Background**

A new "TeleSleep" quality improvement (QI) program was implemented in 2016 at the Roudebush VA Medical Center in Indianapolis that brought the staff of the local Sleep and Telehealth services together for the first time. The Implementation Core (IC) of the VA Precision Monitoring (PRIS-M) QUERI sought to understand how staff in different services and job positions experienced and perceived ongoing implementation of the QI initiative.

**Methods**

The Participant-Reported Implementation Update and Score (“PRIUS”) is a five-minute check-in method developed and piloted by the VA PRIS-M IC designed to elicit how key participants view implementation at different points in time. PRIUS sessions took place approximately every two or three weeks as an in-person or phone conversation between participant and IC member. Participants responded verbally to the question "What are some things that happened over the past 2-3 weeks that seem relevant from your perspective to the implementation of the TeleSleep project"? Participants then verbally scored each reported "update" with a number ranging from +3 to -3. Positive scores indicated a positive influence on the implementation process; negative scores indicated a negative influence; and zero indicated neutral influence. A "3" indicated a strong influence, "2" moderate, and "1" weak.

**Findings**

Five members of the IC conducted PRIUS sessions with 12 different staff members involved in the Telesleep project over a 6-month period in 2016. There were 62 different PRIUS sessions containing a total of over 225 updates; the average PRIUS session had 3-4 updates. New PRIUS entries were discussed twice a month during IC meetings; major developments and themes were shared with the lead investigator of TeleSleep. PRIUS findings included that Sleep and Telehealth staff reported fundamentally different perspectives on TeleSleep implementation; that the TeleSleep project coordinator played a key boundary-spanning role critical to implementation success; and that a seemingly modest event (a catered "appreciation" lunch for TeleSleep staff) unexpectedly proved to be a turning point in TeleSleep implementation.

**Implications for D&I Research**

The PRIUS method provides an efficient, structured way to elicit and capture perspectives of diverse participants on local implementation-related developments over time, generating new sources of both qualitative and numerical data for analysis.

**Primary Funding Source**

Department of Veterans Affairs - funded by VA QUERI program

### S113 National Institutes of Health Pathways to Prevention program: weighing the evidence, identifying the research gaps, and disseminating to the prevention research community

#### Kate Winseck, Carrie Klabunde, Deb Langer, Avi Aggarwal, Elizabeth Neilson

##### Office of Disease Prevention, National Institutes of Health, Bethesda, MD, 20892, USA

###### **Correspondence:** Kate Winseck

**Background**

Developing informed, comprehensive approaches to disease prevention has many challenges, including conflicting evidence and research gaps. The Pathways to Prevention (P2P) program, sponsored by the NIH Office of Disease Prevention, convenes workshops that help build consensus on a research topic, shape future research agendas, and disseminate action plans for researchers and federal agencies. This process addresses needs identified in the Knowledge Translation for Research Utilization Framework.

**Methods**

The P2P program identifies research needs for a selected topic through an evidence review, a comprehensive workshop featuring diverse expert perspectives, and an unbiased analysis by a panel of scientists external to the field. A federal partners’ meeting is convened to discuss federal agency actions based on workshop recommendations. The identified needs and recommendations are disseminated through published reports: a Systematic Evidence Review, the Workshop Panel’s final report, and Federal Partners Meeting report, and other digital communication tools.

**Findings**

To date, five P2P workshops have been conducted, addressing youth suicide (2016), Total Worker Health® (2015), myalgic encephalomyelitis/chronic fatigue syndrome (2014), opioids and chronic pain (2014), and polycystic ovary syndrome (2012). Workshop findings and recommendations have served as a catalyst to advance these fields. For example, published reports from the P2P workshop on opioids collectively yielded 177 citations and 379 second generation citations (July 2016), and the federal action plan was used by the NIH Pain Consortium to create a new research program addressing the complex challenges of effectively treating chronic pain.

**Implications for D&I Research**

The dissemination of P2P findings draws attention to scientific topics of broad public health importance and their significant research gaps. The P2P program addresses barriers within the Knowledge Translation for Research Utilization Framework^1^, especially within the knowledge creation, knowledge transfer, and research utilization phases. Broader application of the program’s evidence-based process can assist research and policy development.

**Primary Funding Source**

National Institutes of Health

### S114 The good coach: reciprocal influences of outer and inner context on coaching as fidelity support during evidence-based intervention implementation and sustainment

#### Lara Gunderson

##### Behavioral Health Research Center of the Southwest, Pacific Institute for Research and Evaluation, Albuquerque, NM, 87102, USA

**Background**

Evidence-based intervention (EBIs) implementation unfolds within complicated social, political, and economic contexts and is influenced by the attitudes and behaviors of diverse stakeholders situated within these environments. Coaching is commonly regarded as an effective strategy to support service providers in the delivery of EBIs and attainment of high levels of fidelity over time. The purpose of this poster is to address a gap in research concerning factors that influence coaching as a key EBI support strategy.

**Methods**

Data were collected in eleven child welfare service systems as part of a prospective (> 10 year) mixed-method examination of implementation and sustainment of one EBI to prevent child neglect and related health problems. We conducted individual semi-structured interviews (*n =* 166), small group interviews averaging three participants each (*n =* 13), and focus groups averaging six participants (*n =* 80) with a wide range of stakeholders in all eleven service systems. We use the Exploration, Preparation, Implementation, and Sustainment (EPIS) framework to consider inner-context (i.e., within service delivery organizations) and outer-context (i.e., system level) factors affecting coaching over time. Analyses included open and focused coding to derive predominant themes and issues related to coaching implementation and sustainment.

**Findings**

The analysis pointed to six interrelated themes: perceptions of coaches by sustainment status; coach as peer; inner-context coaching capacity; EBI developer requirements versus outer-context needs; outer-context support; and inner-context support. Coaches characterized as successful were those who effectively navigated and negotiated changing inner and outer contexts throughout the EPIS Implementation phase and into the Sustainment phase. Coaches were often described by other stakeholders as operating in a boundary-spanning capacity.

**Implications for D&I Research**

Supportive coaching relationships can be fostered by conscious efforts to integrate and institutionalize the role of coaching in the implementation and sustainment of EBIs. Providers and coaches in the present study identified coaches as boundary spanners who link inner and outer contexts, and facilitate communication pertaining to implementation. However, we caution government and CBO administrators that while it may be valuable for coaches to span the inner and outer context, it may also compromise the integrity of their specific role within EBI implementation and sustainment.

**Primary Funding Source**

National Institutes of Health - R01MH072961 and R01MH092950

### S115 Use of natural language processing to support prevention of non-elective rehospitalization

#### Gabriel J Escobar^1^, Marla Gardner^1^, Liam O’Sulleabhain^1^, Candyce Kroenke^1^, Vincent Liu^2^, Patricia Kipnis^3^

##### ^1^Division of Research, Kaiser Permanente, Oakland, CA, 94612, USA; ^2^Health Care Effectiveness, Kaiser Permanente Division of Research, Oakland, CA, 94612, USA; ^3^Decision Support, Kaiser Permanente Northern California, Oakland, CA, 94612, USA

###### **Correspondence:** Gabriel J Escobar

**Background**

Rehospitalization prevention efforts have been hampered by limitations of existing predictive models. Information on non-clinical factors (e.g., social support, functional status) found in clinicians’ free text notes could enhance prediction. We describe data extraction from free text notes using natural language processing (NLP) and challenges to the use of such data for prediction and prevention of rehospitalization.

**Methods**

Using commercially available NLP software (I2E, Linguamatics), we extracted data from clinician free text notes. Our study cohort consisted of 360,036 adults who experienced 609,393 hospitalizations at 21 Kaiser Permanente Northern California hospitals from 6/1/10-12/31/13. Data were extracted as individual “atoms” (“patient lives with daughter” or “patient lives with spouse”) that we combined into “molecules,” (“patient lives with family OR spouse OR daughter …”) permitting comparisons with groups of patients characterized by other “molecules” (e.g., living alone). We also interviewed 1,152 patients to enable comparison to validated measures of social support and functional status.

**Findings**

We extracted 6,218,897 free text notes written by physicians, nurses, social workers, discharge planners, and physical therapists. Data were formatted into 376 individual “atoms.” Concordance between NLP and interviews was good: 86.4% of patients reporting living with others had confirmatory NLP data. Substantial data collection bias was present: sicker patients were much more likely to have free text information on support or functional status. For example, data indicating that a patient was living with a professional caretaker (nurse, paid helper or caregiver) was found in only 1,740 patients, whose average acute physiology score was 81 ± 36 and whose average comorbidity score was 68 ± 53; in contrast, patients with no information (N = 41,012) had scores of 39 ± 32 and 31 ± 38, respectively. When information was available, indicators of social support did enhance ability to predict readmission, although associations varied by patients’ acute severity of illness, comorbidity burden, and type of diagnosis.

**Implications for D&I Research**

Systematic biases in data availability constitute a major limitation to the use of NLP data for rehospitalization prediction and prevention. It is unlikely that NLP data can be used in isolation – counterfactual methods as well as judicious combination with data from formal interviews may be required.

**Primary Funding Source**

Grant from Gordon and Betty Moore Foundation

